# Supplementary material for: Synthesis and Biological Evaluation of Quercetagetin Derivatives as the Inhibitors of Mcl-1 and Bcl-2 Against Leukemia
Source: Int J Mol Sci. 2025 Mar 18;26(6):2727. doi: 10.3390/ijms26062727 (PMC11943384; doi:10.3390/ijms26062727)
Supplement: Supplementary file 1 [file ijms-26-02727-s001.zip › ijms-3436934-supplementary.pdf]

Synthesis and Biological Evaluation of Quercetagetin Derivatives as the Inhibitors of Mcl-1 and Bcl-2 to Against Leukemia

General information

Column chromatography silica gel (200-300 mesh) and TCL plate were purchased from Qingdao Meijin Chemical Inc (Qingdao; China); HRMS data were obtained in the ESI mode on an Agilent 6530 Q-TOF/MS system. ^{1}H NMR and ^{13}C NMR spectra were recorded on Bruker 400 MHz spectrometer. Coupling constants (J) are expressed in hertz (Hz). Chemical shifts (δ) of NMR are reported in parts per million (ppm) units relative to an internal control (TSM).

General procedure for preparation of compounds 2a and 3a

Quercetagetin (1590 mg, 5 mmol) was dissolved in 25 mL DMF and then K_2CO_3 (3105 mg, 22.5 mmol, 4.5 eq) was added in the solvent, stirred and CH_3I (1401 μL, 22.5 mmol, 4.5 eq) was dropped in the reaction system and stirred 3d at room temperature. The reaction process was monitored by TLC. After the reaction was complete, the reaction mixture was extracted by ethyl acetate and water (50:50 mL) three times, gather organic phase, the organic phase was concentrated by reduced pressure to get the crude product, which was purified by column chromatography on silica gel (2:1 = Hexane:EtOAc and containing 3% volume of CHOOH) to get compounds **2a** and **3a**.

General procedure for preparation of compounds 2b and 3b

Take **2b** for instance, **2a** (374 mg, 1 mmol) was dissolved in 5 mL THF and K_2CO_3 (207 mg, 1.5 mmol, 1.5 eq) was added following, stirred and $BrC_2H_2OOC_2H_5$ (167μL, 1.5 mmol, 1.5 eq) was dropped in reaction mixture and stirred 12 h at 60 °C. The reaction process was monitored by TLC. After the reaction was complete, the reaction mixture was extracted by ethyl acetate and water (20:20 mL) three times, gather organic phase, the organic phase was concentrated by reduced pressure to get the crude product, which was purified by column chromatography on silica gel (1:1 = Hexane:EtOAc) to get **2b**.

General procedure for preparation of compounds 2c-2j and 3c-3j

Take **2c-2j** for instance, **2b** (92 mg, 0.2 mmol) was dissolved in anhydrous DCM (3~5 mL) and EDCI (114 mg, 0.6 mmol, 3 eq) was added in reaction system and amino acid ester (0.4 mmol, 2 eq) was added in reaction mixture, stirred 24 h at room temperature. The reaction process was monitored by TLC. After the reaction was complete, the reaction mixture was concentrated by reduced pressure and re-dissolved in 5 mL 1N NaOH H_2O/acetone, stirred for another 4h at 40 °C, after that, the reaction mixture was extracted by ethyl acetate and 3% HCl (20:20 mL) three times, gather organic phase, the organic phase was concentrated by reduced pressure to get compound **2c-2j**.

General procedure for preparation of compounds 2k-2t

2a (74.8 mg, 0.2 mmol) was dissolved in 3~5 mL DMF and K_2CO_3 (33.1 mg, 0.24 mmol, 1.2 eq) was added in this solvent, stirred and halohydrocarbon (0.24 mmol, 1.2 eq) was dropped in the reaction system stirred and reacted at 60 °C for 24 h. The

reaction process was monitored by TLC. After the reaction was complete, the reaction mixture was extracted by ethyl acetate and water (20:20 mL) three times, gather organic phase, the organic phase was concentrated by reduced pressure to get the crude product, which was purified by column chromatography on silica gel (2:1 ~ 5:1= Hexane:EtOAc) to get **2k-2t**.

General procedure for preparation of compounds 4a-4g

Quercetagetin (159 mg, 0.5 mmol) was dissolved in 10 mL DMF and then K_2CO_3 (310.5 mg, 2.25 mmol, 4.5 eq) was added in the solvent, stirred and the halohydrocarbon (2.25 mmol, 4.5 eq) was dropped in the reaction system stirred and reacted at room temperature for 72 h. After the reaction was complete, the reaction mixture was extracted by ethyl acetate and water (25:25 mL) three times, gather organic phase, the organic phase was concentrated by reduced pressure to get the crude product, which was purified by column chromatography on silica gel (3:1 ~ 15:1= Hexane:EtOAc and containing 3% volume of CHOOH) to get **4a-4g**.

^{1}H NMR and ^{13}C NMR spectra of compounds 2a-2t, 3a-3j and 4a-4g

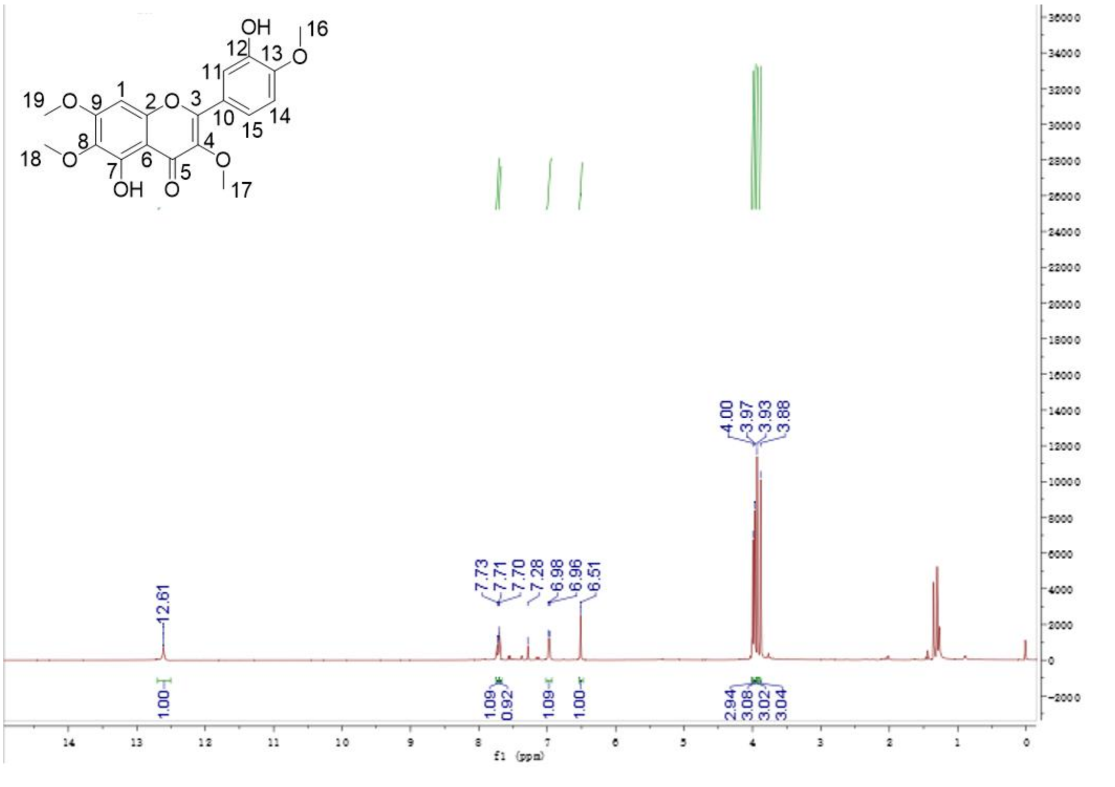

Figure S1 ^{1}H NMR spectra of compound **2a**

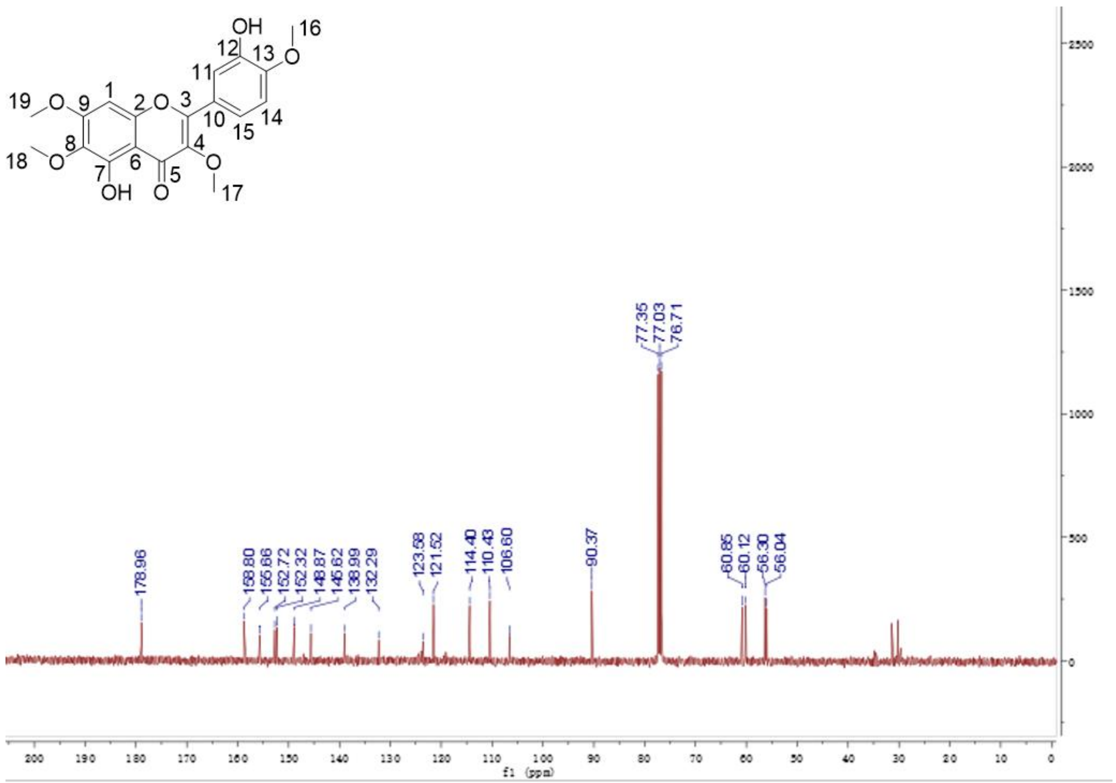

Figure S2 ^{13}C NMR spectra of compound **2a**

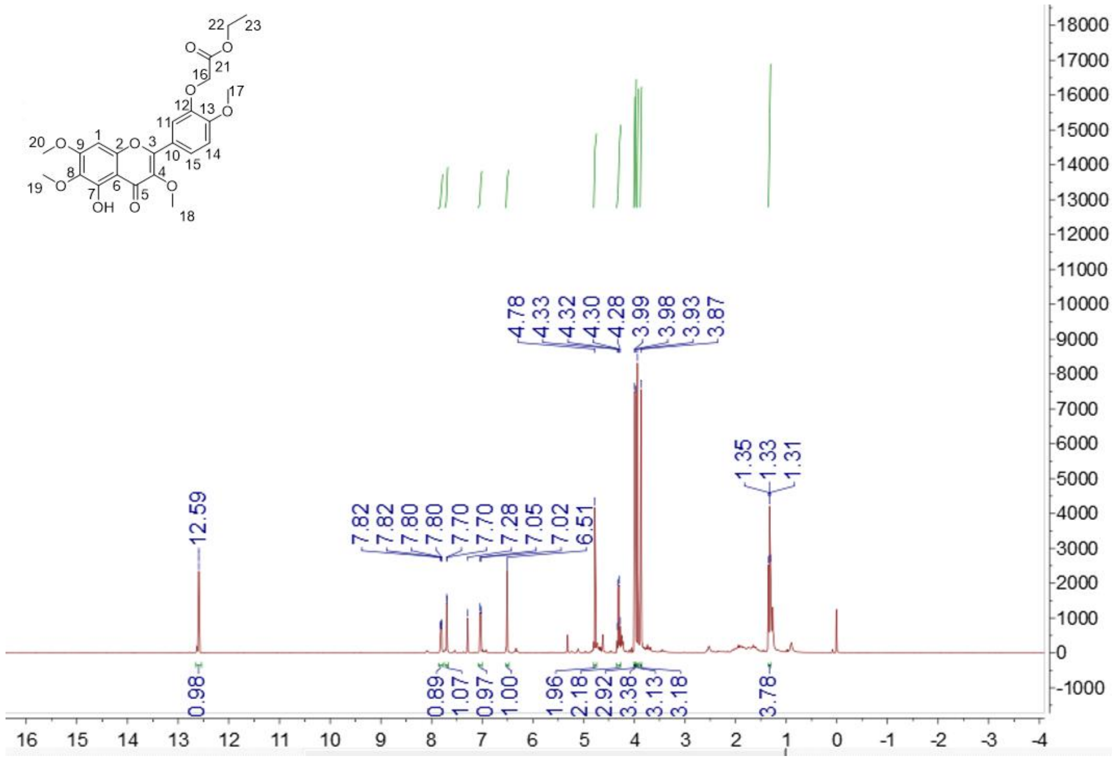

Figure S3 ^{1}H NMR spectra of compound **2b**

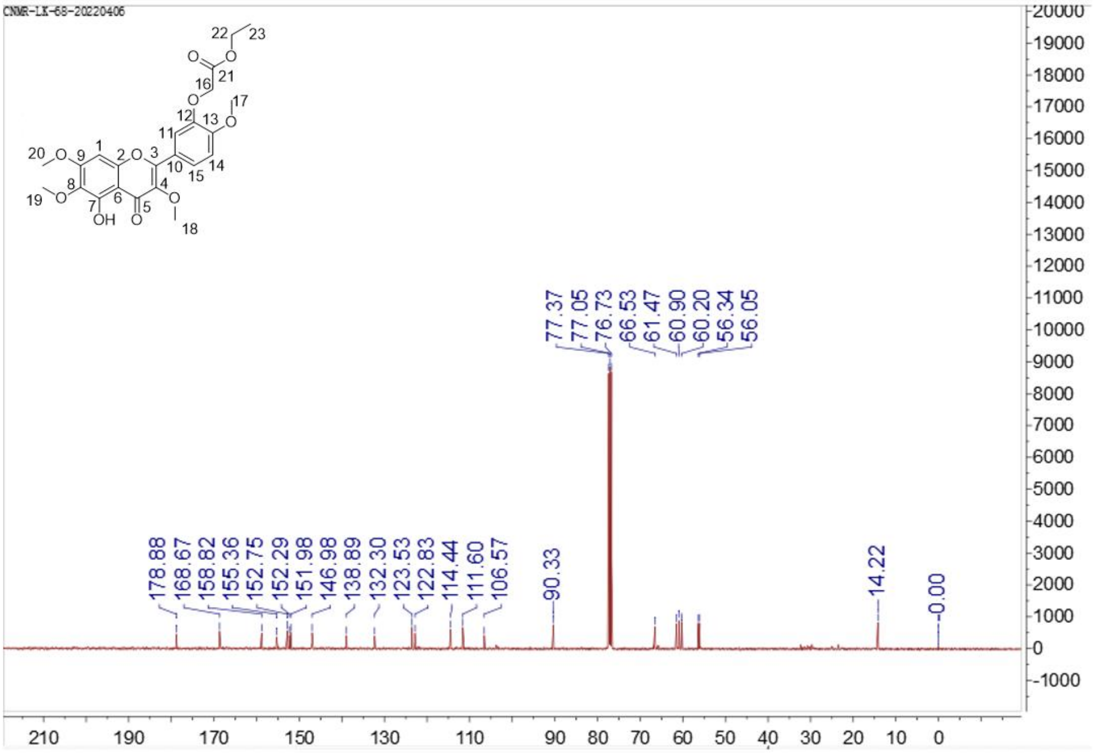

Figure S4 ^{13}C NMR spectra of compound **2b**

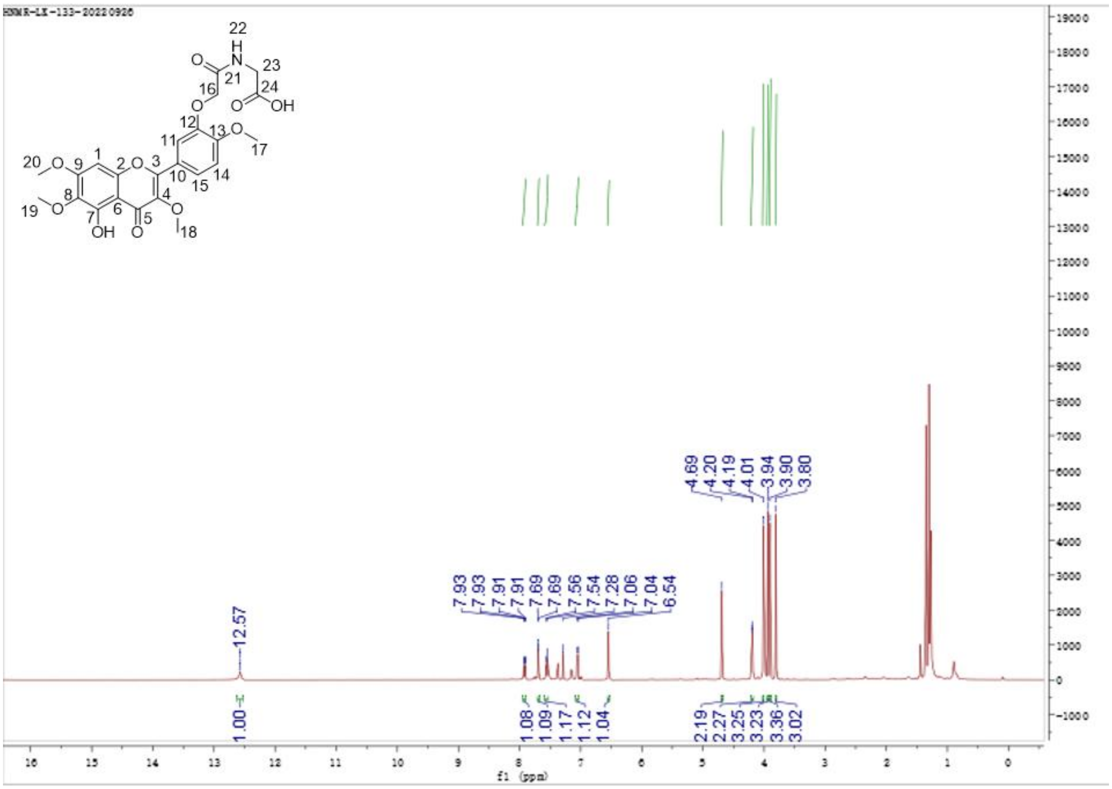

Figure S5 ^{1}H NMR spectra of compound **2c**

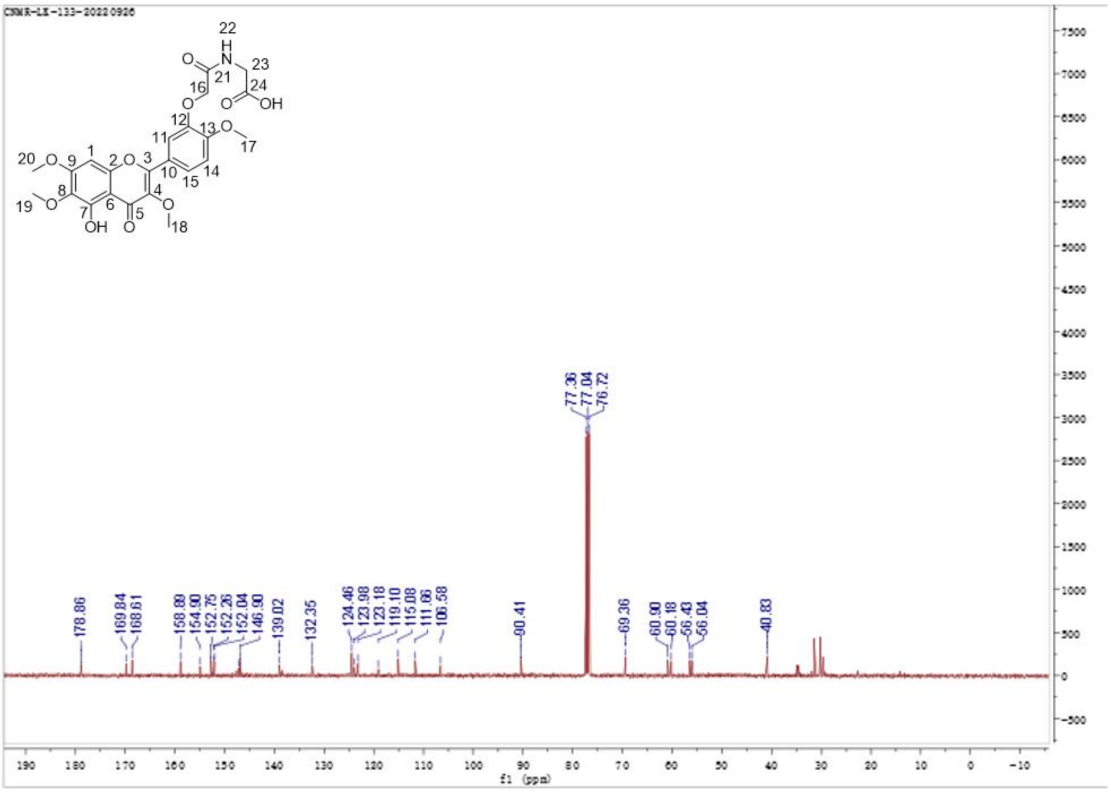

Figure S6 ^{13}C NMR spectra of compound **2c**

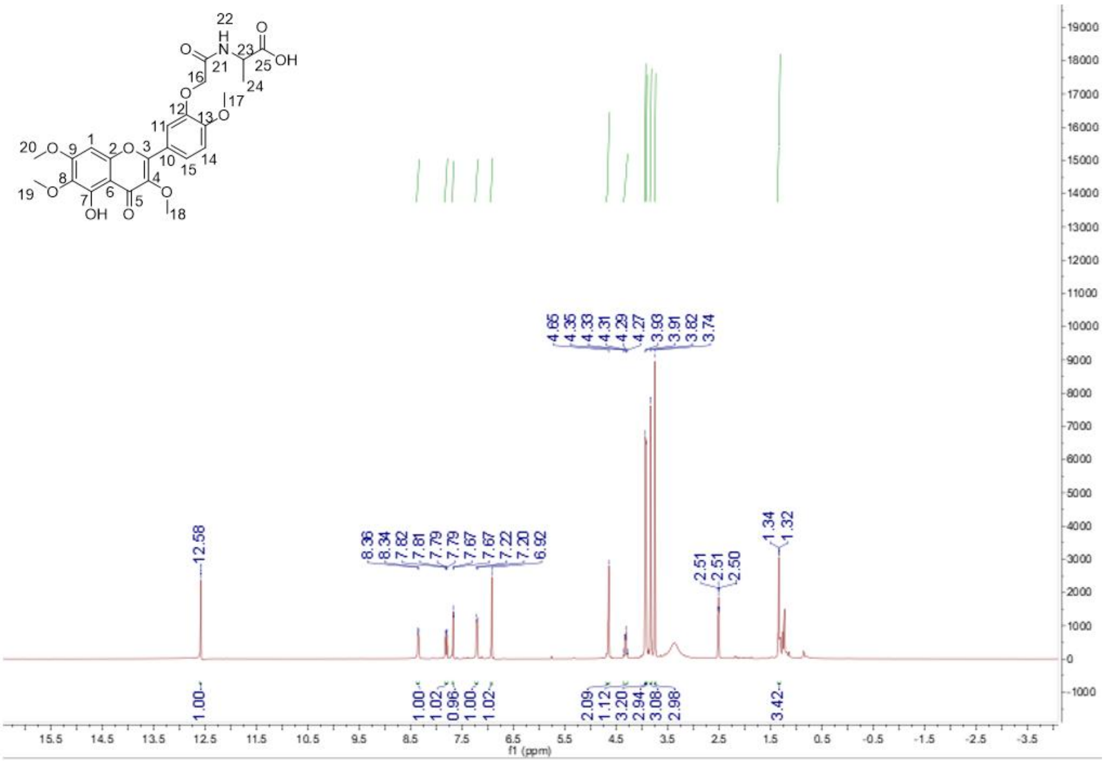

Figure S7 ^{1}H NMR spectra of compound **2d**

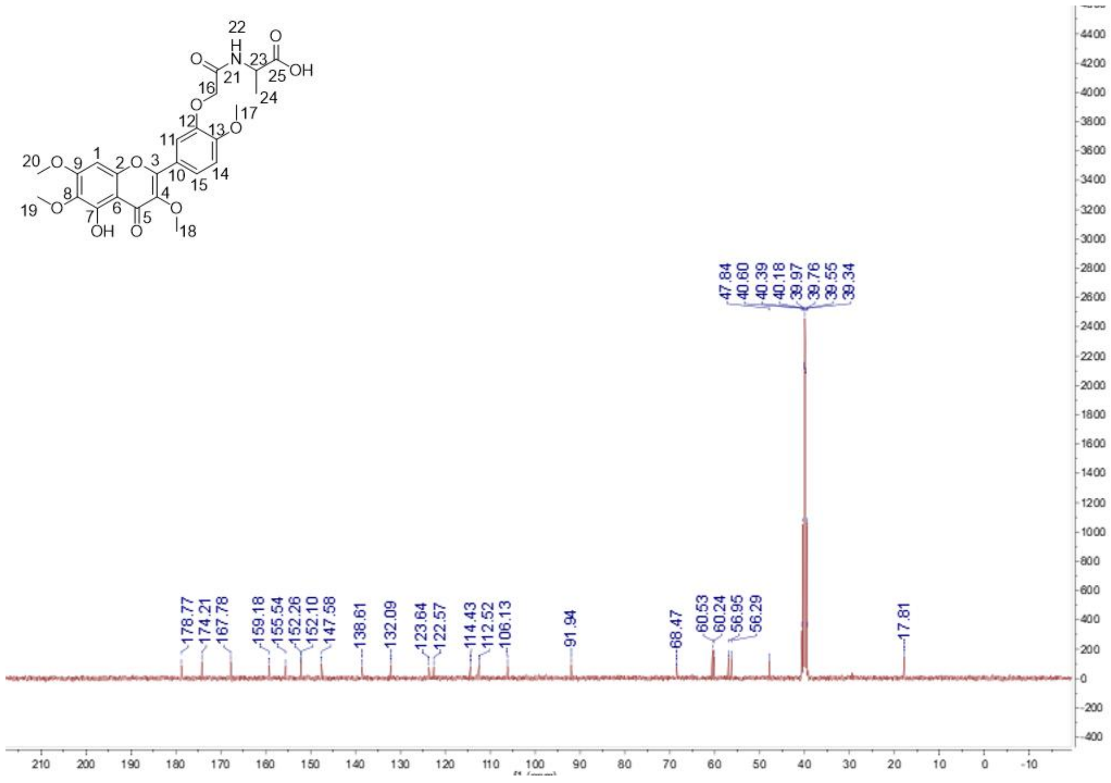

Figure S8 ^{13}C NMR spectra of compound **2d**

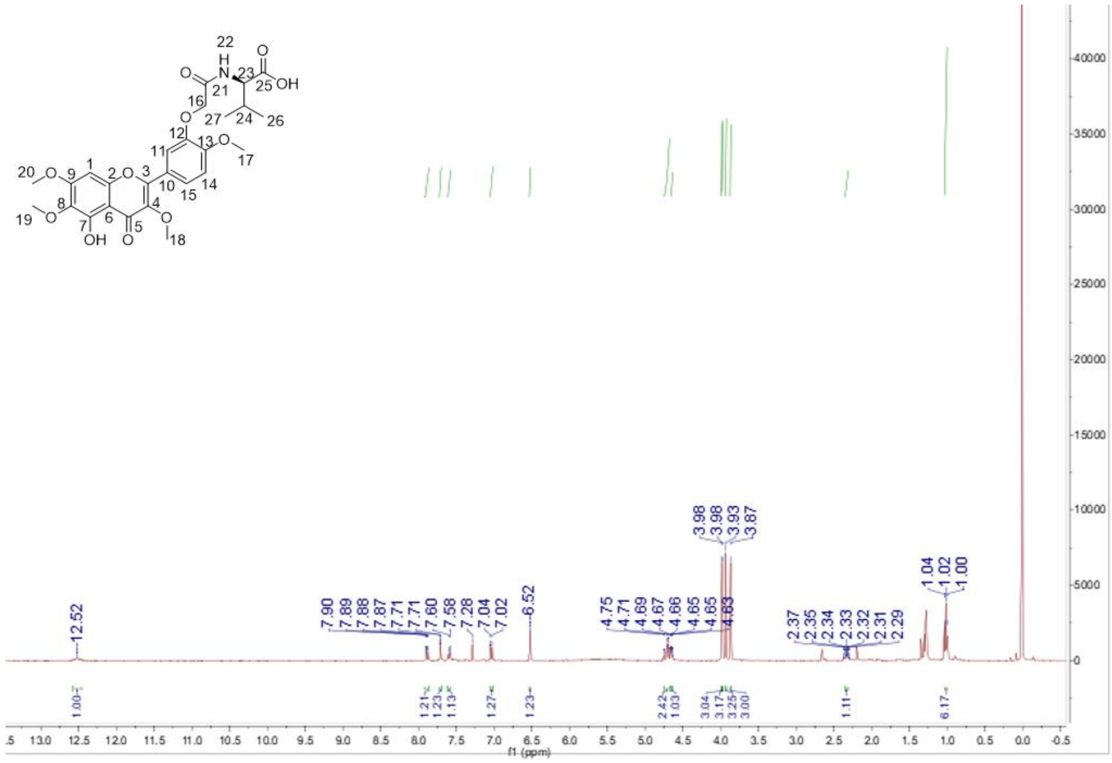

Figure S9 ^{1}H NMR spectra of compound **2e**

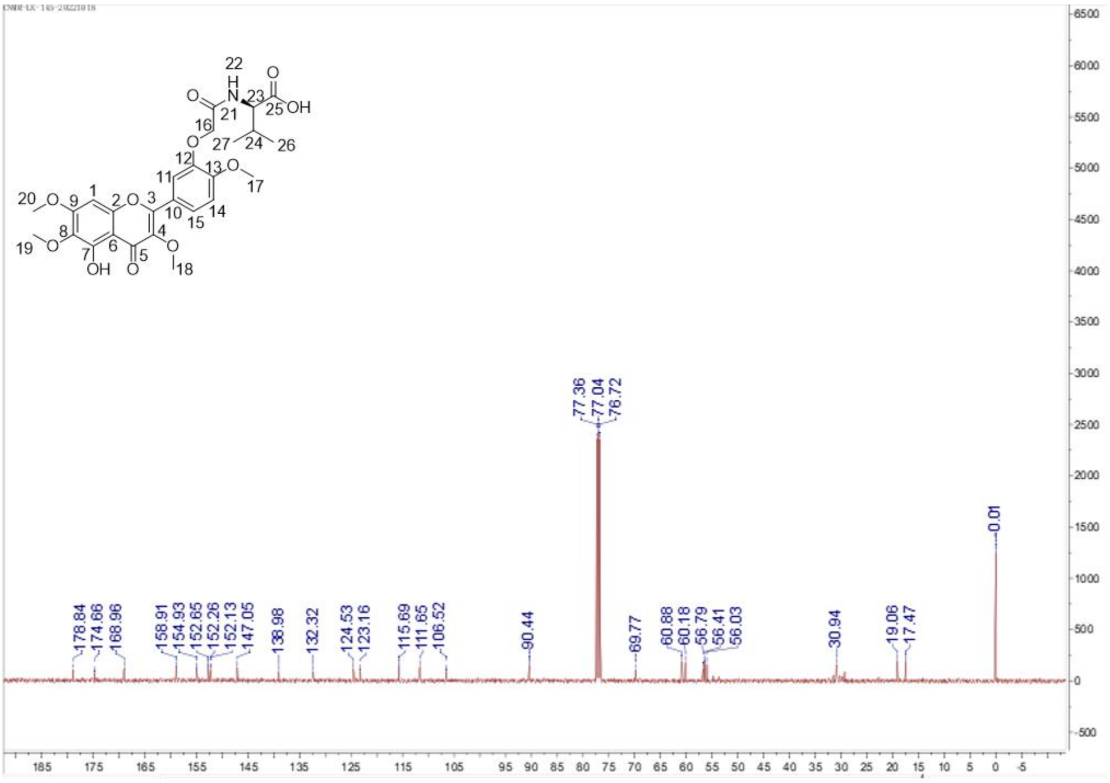

Figure S10 ^{13}C NMR spectra of compound **2e**

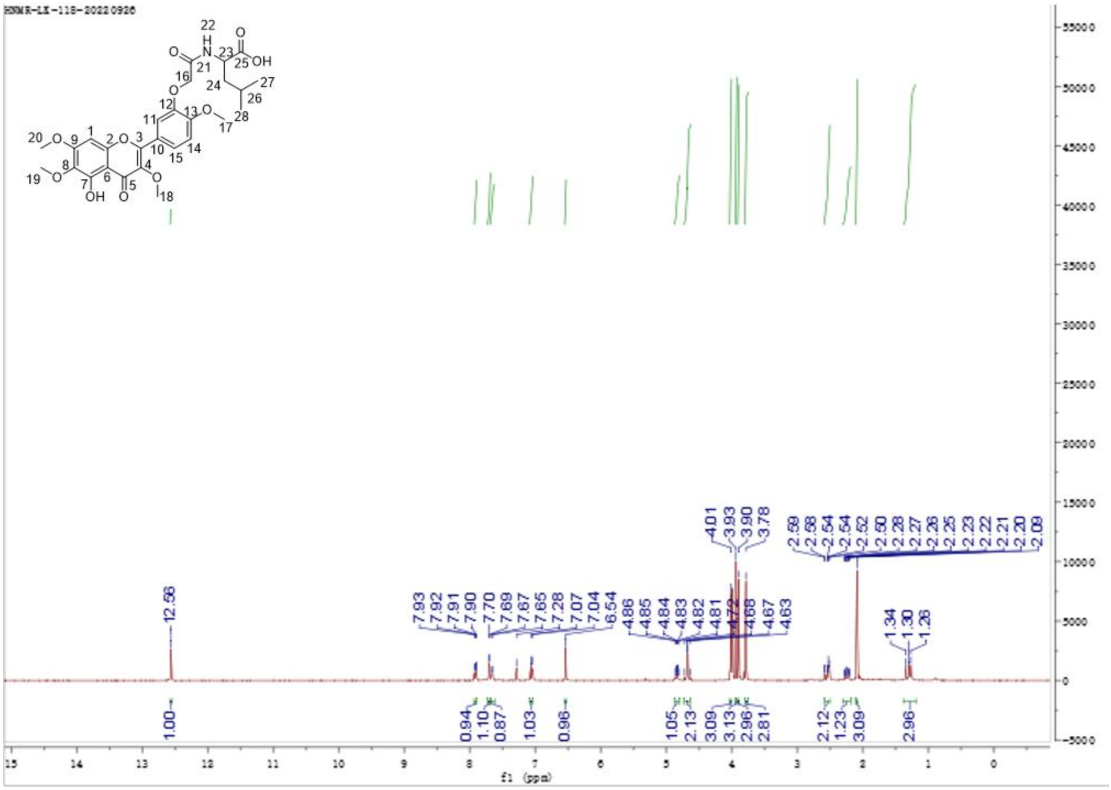

Figure S11 ^{1}H NMR spectra of compound **2f**

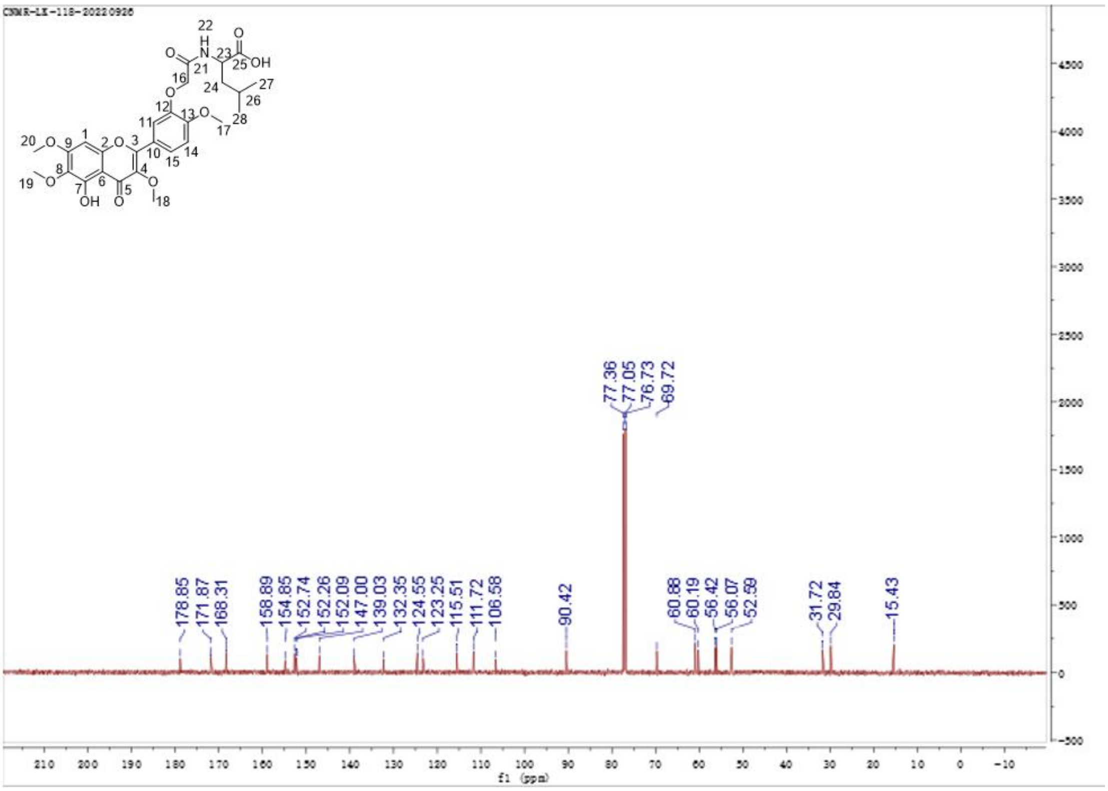

Figure S12 ^{13}C NMR spectra of compound **2f**

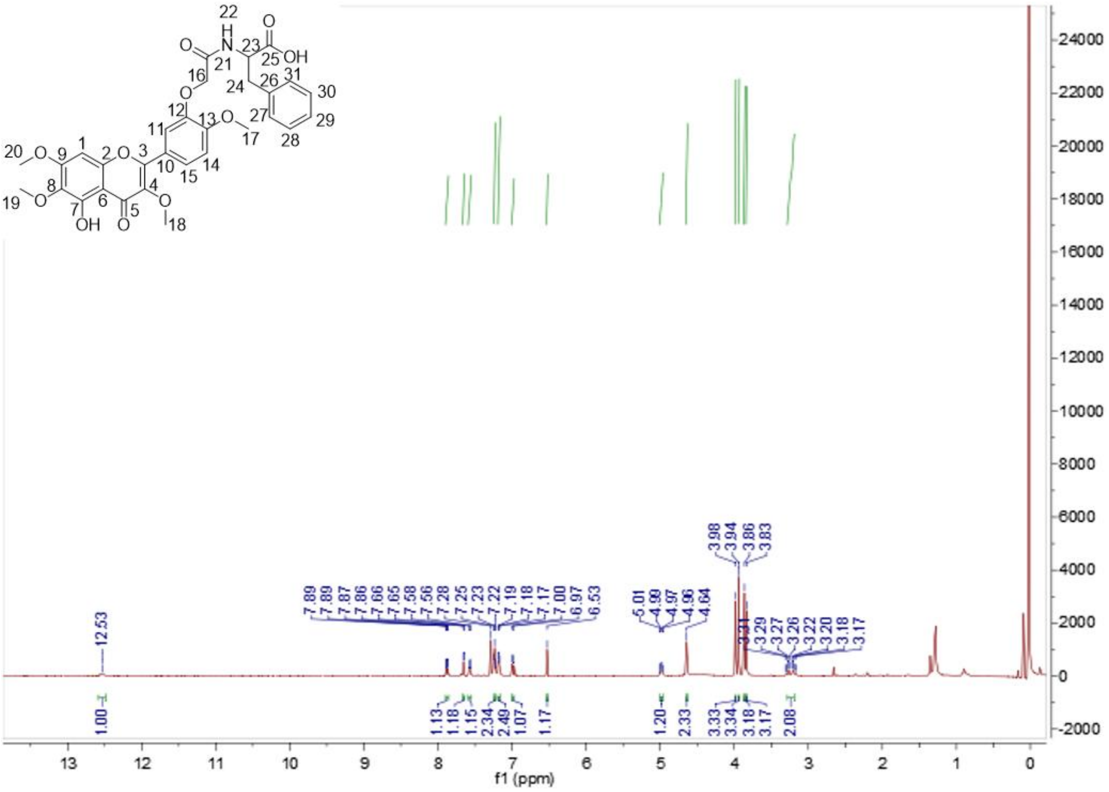

Figure S13 ^{1}H NMR spectra of compound **2g**

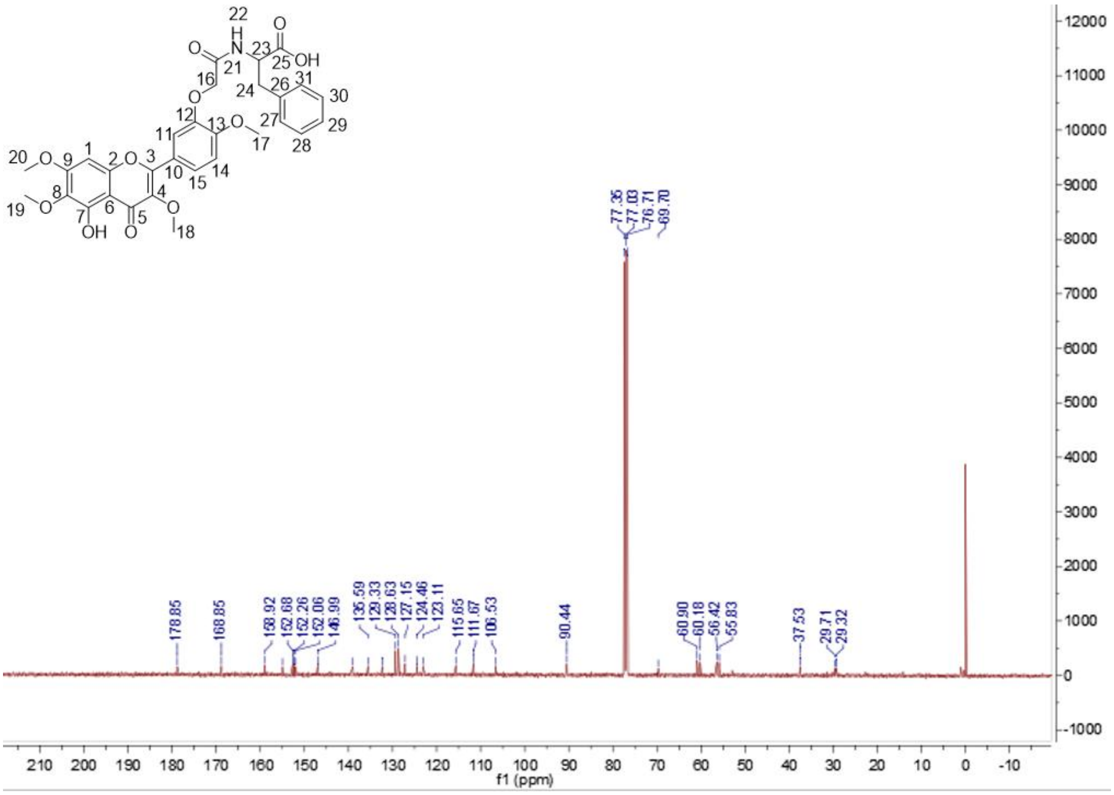

Figure S14 ^{13}C NMR spectra of compound **2g**

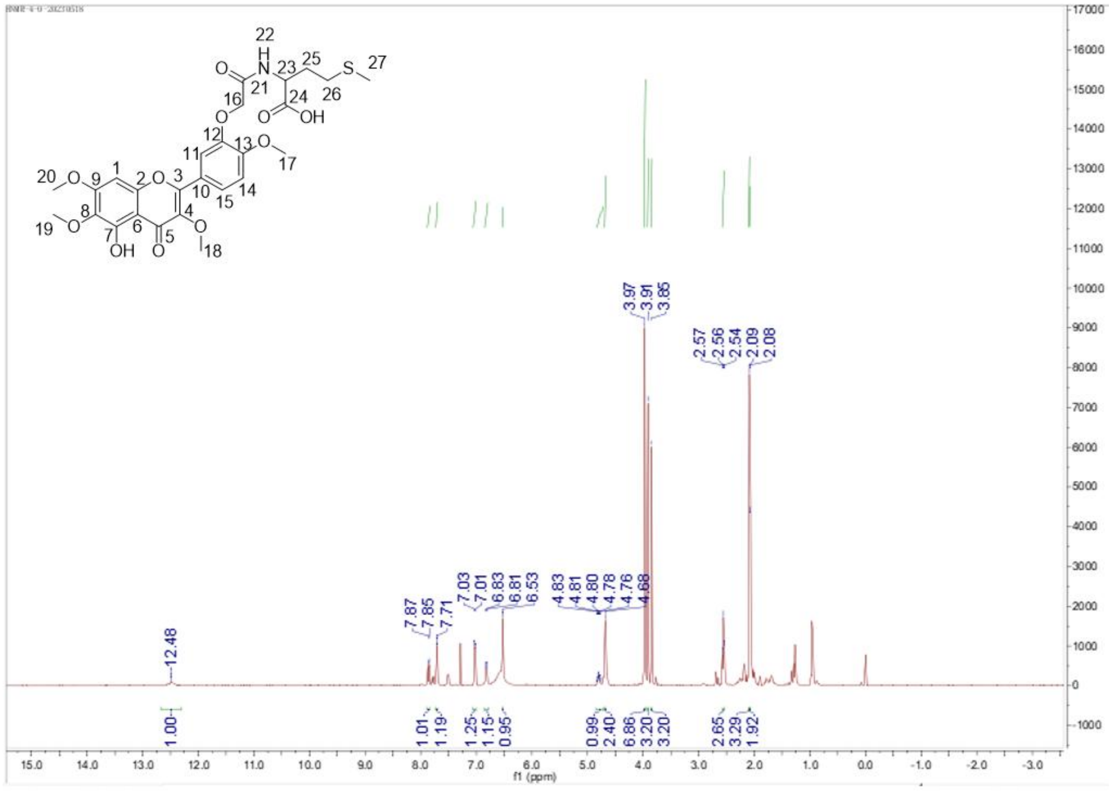

Figure S15 ^{1}H NMR spectra of compound **2h**

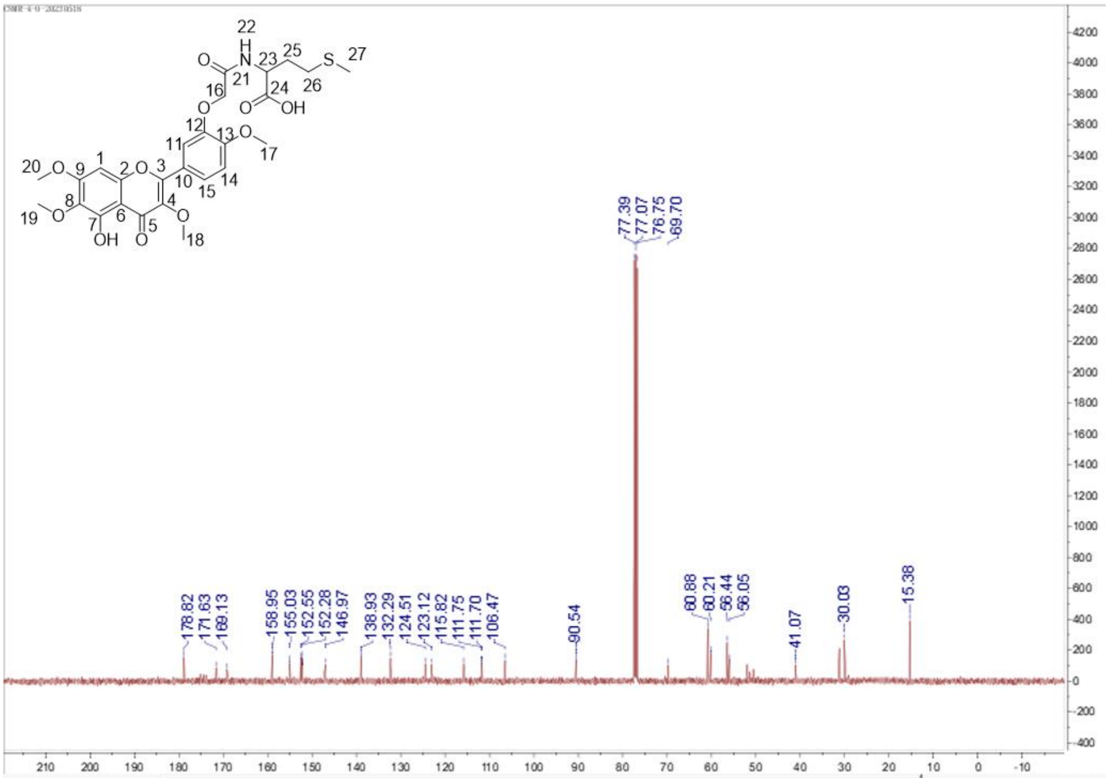

Figure S16 ^{13}C NMR spectra of compound **2h**

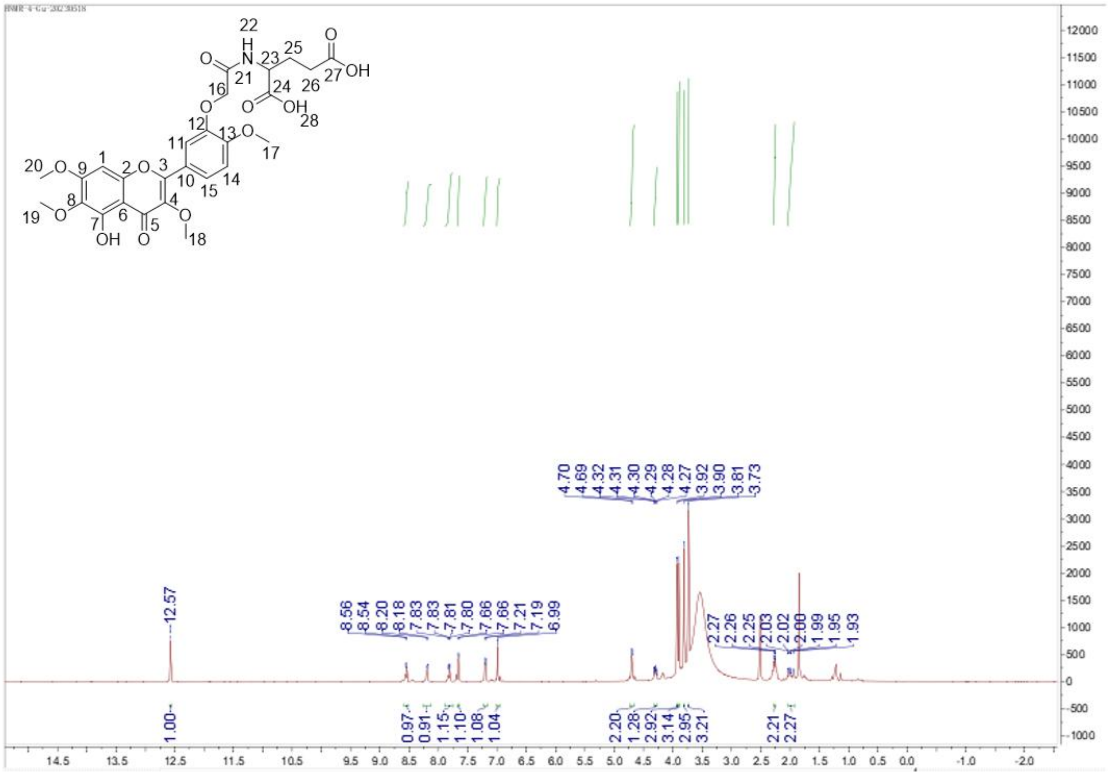

Figure S17 ^{1}H NMR spectra of compound **2i**

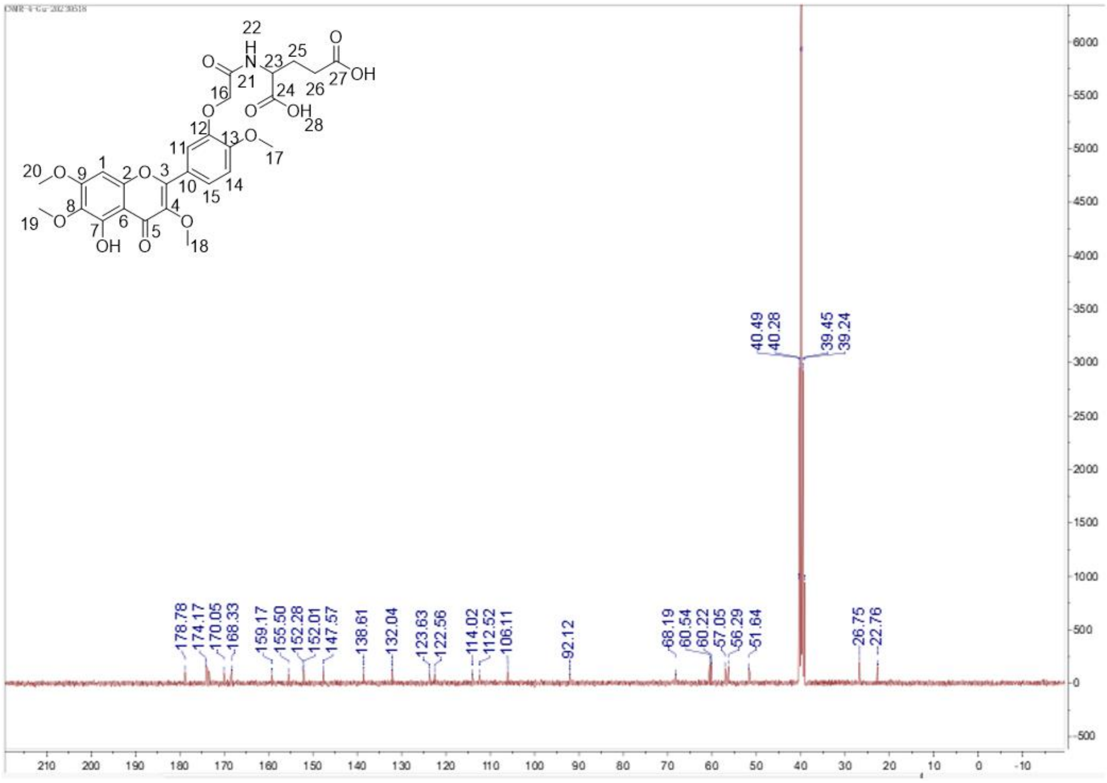

Figure S18 ^{13}C NMR spectra of compound **2i**

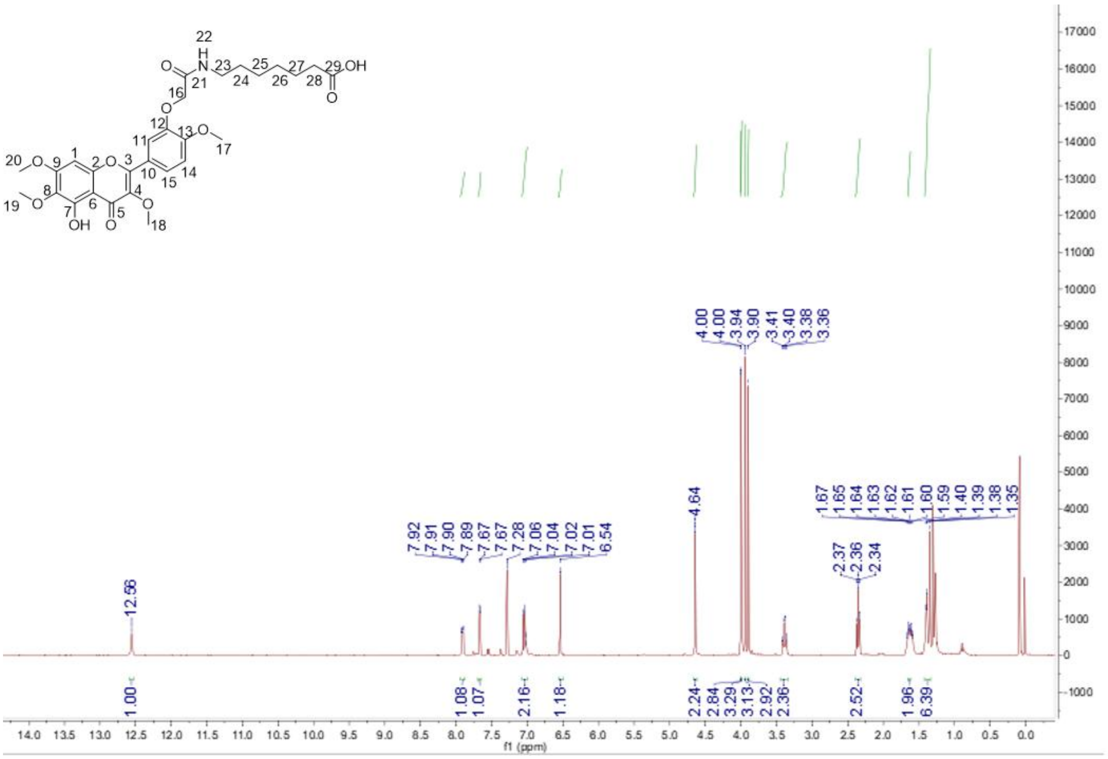

Figure S19 ^{1}H NMR spectra of compound **2j**

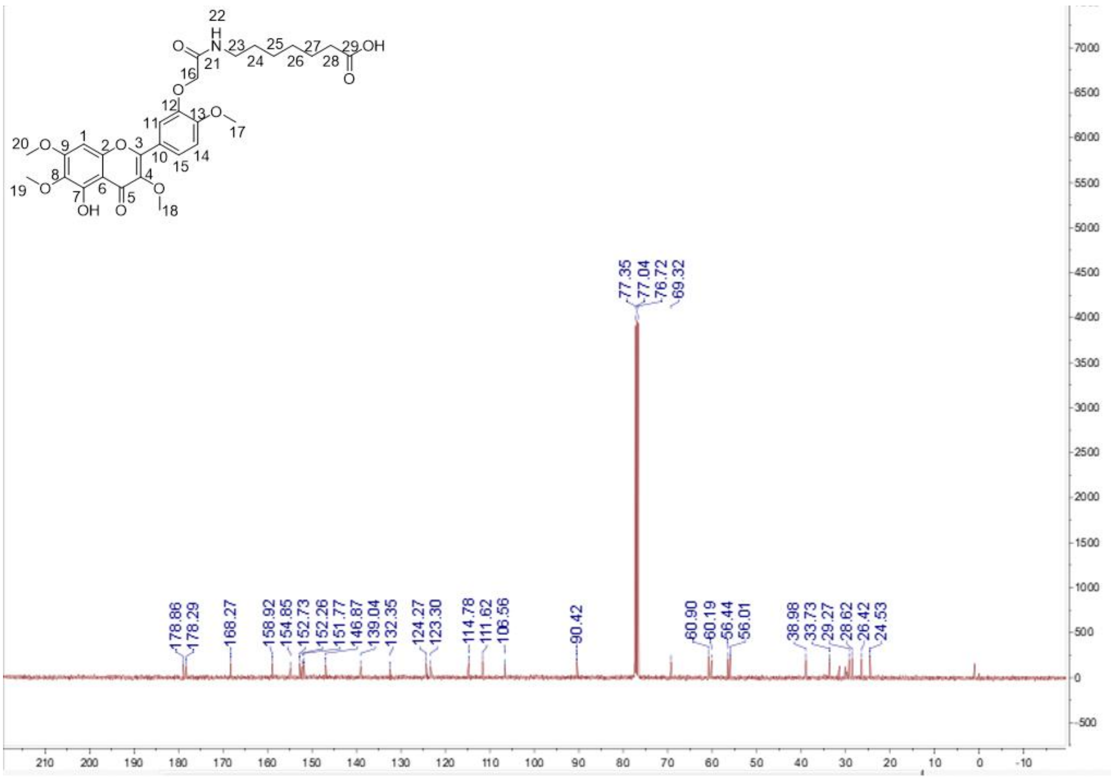

Figure S20 ^{13}C NMR spectra of compound **2j**

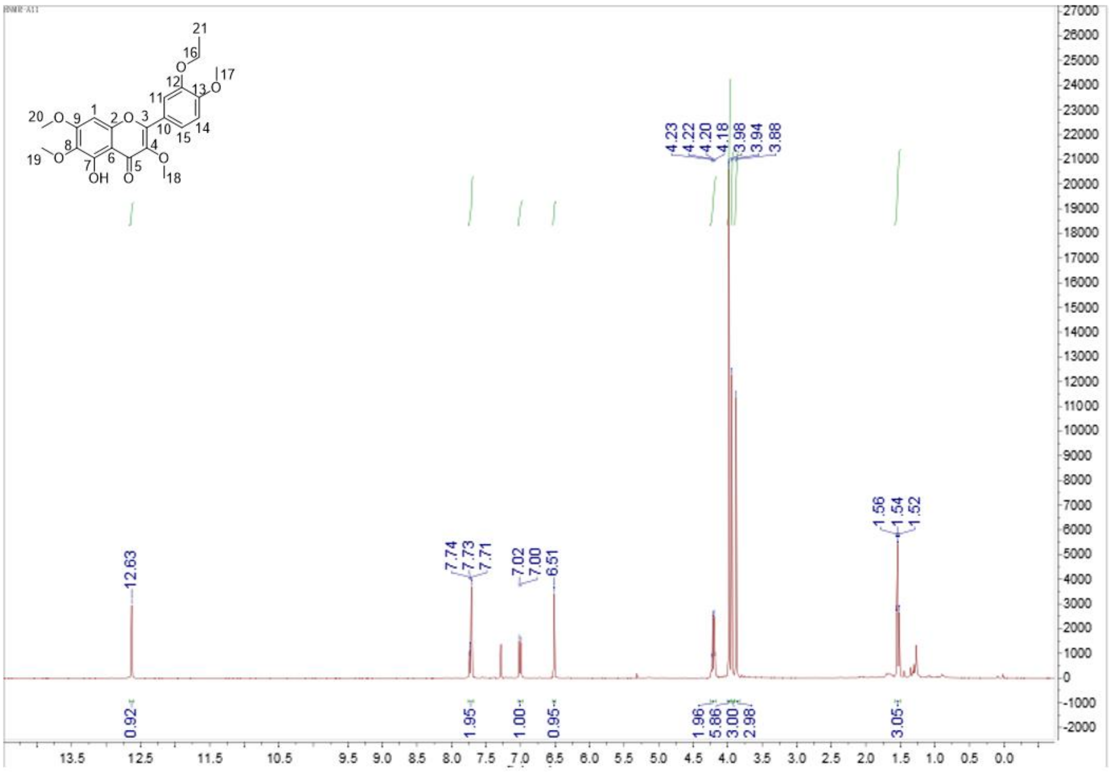

Figure S21 ^{1}H NMR spectra of compound **2k**

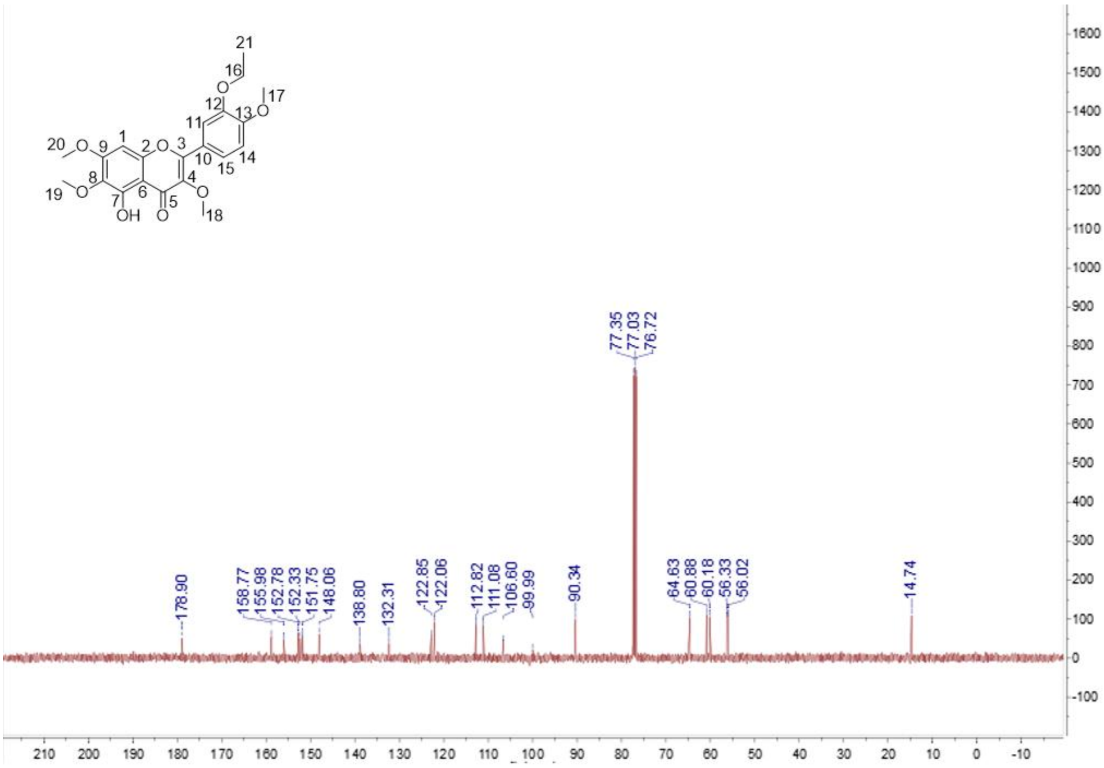

Figure S22 ^{13}C NMR spectra of compound **2k**

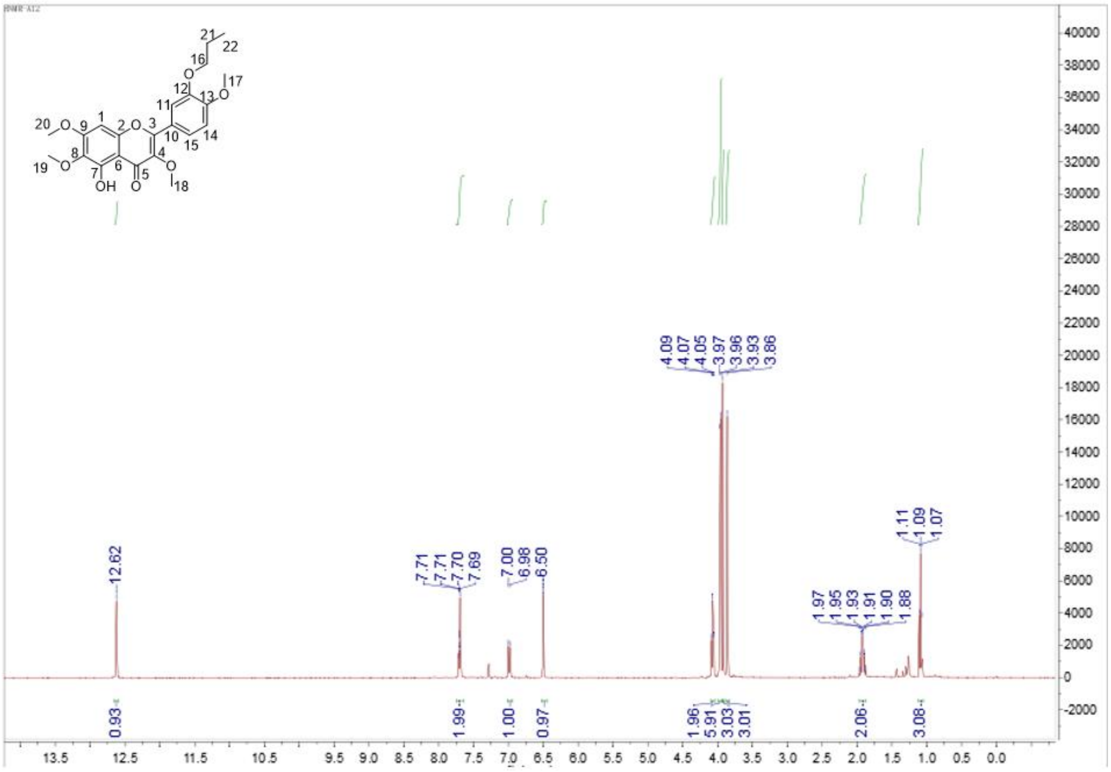

Figure S23 ^{1}H NMR spectra of compound **2l**

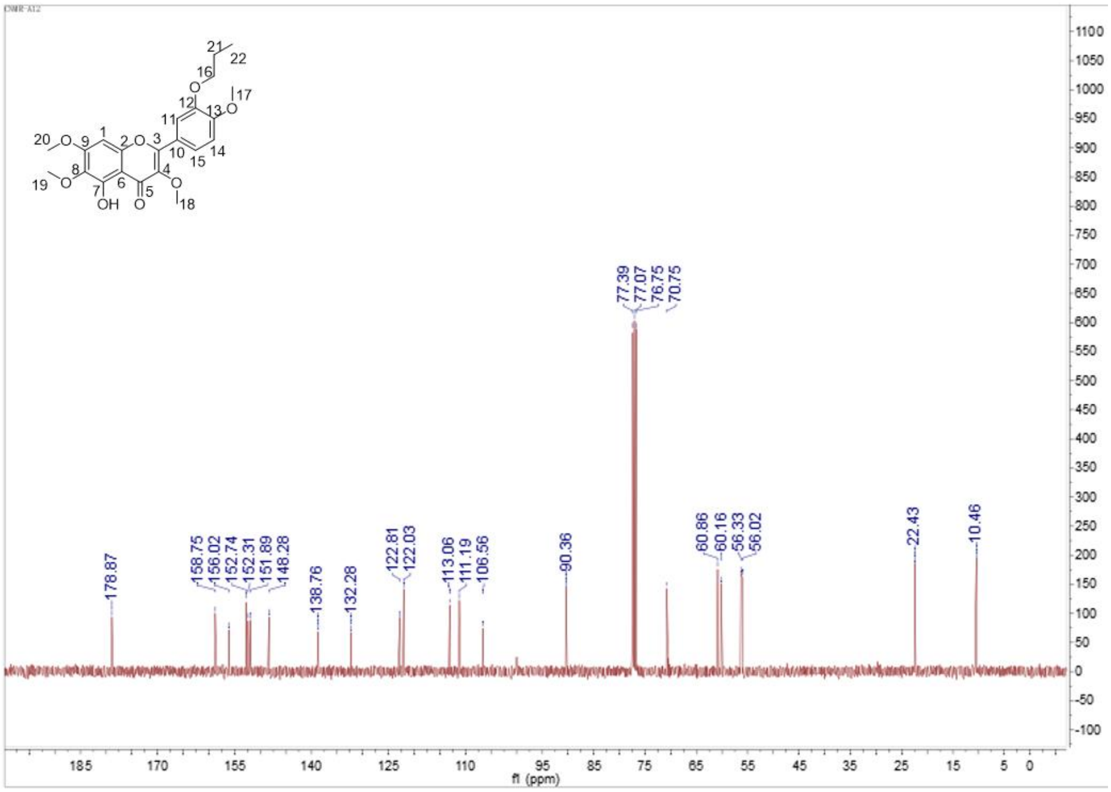

Figure S24 ^{13}C NMR spectra of compound **2l**

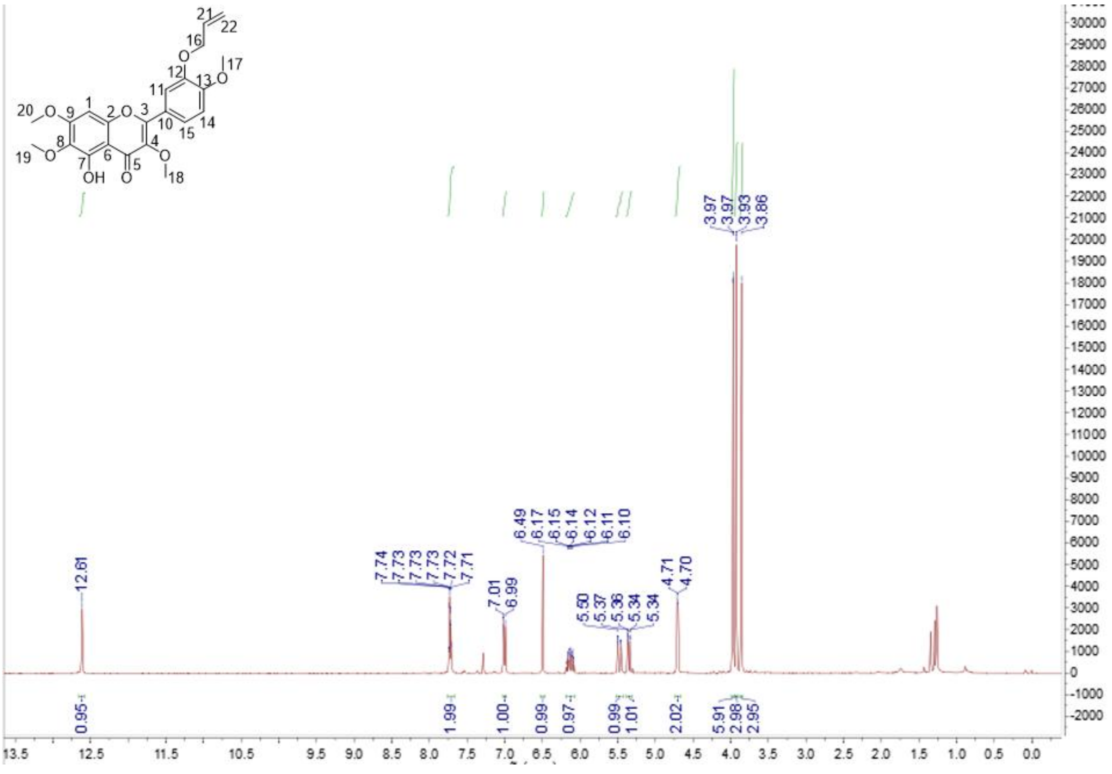

Figure S25 ^{1}H NMR spectra of compound **2m**

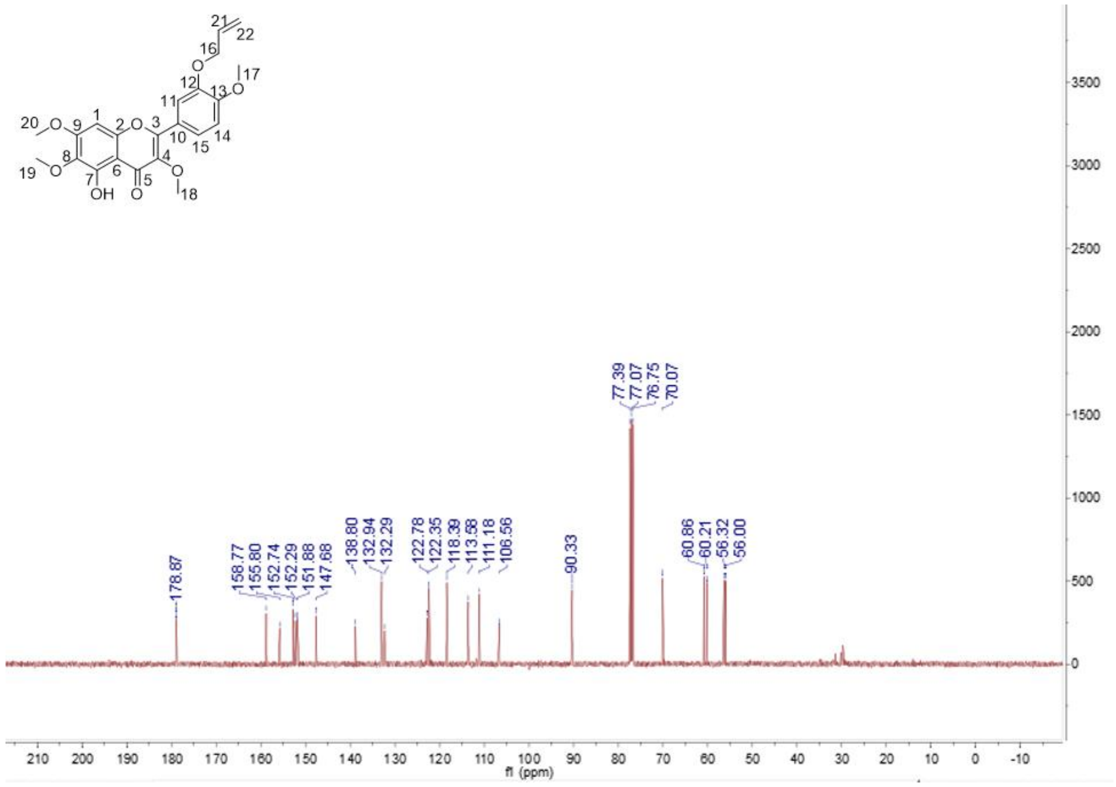

Figure S26 ^{13}C NMR spectra of compound **2m**

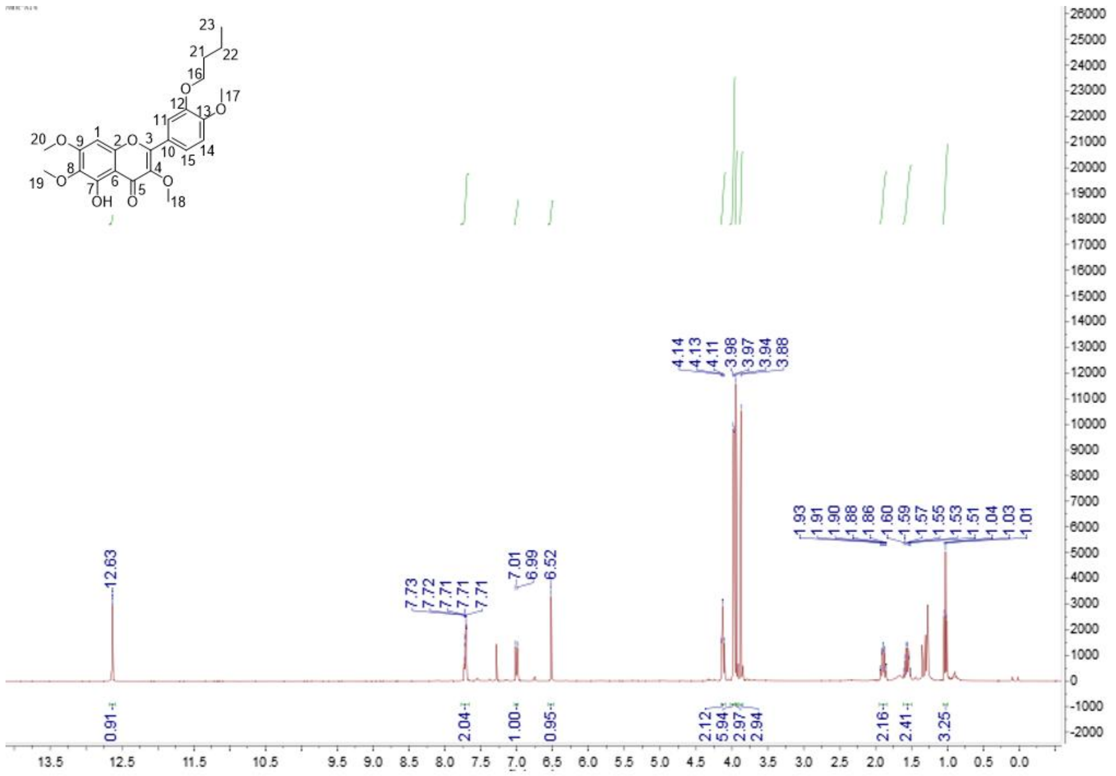

Figure S27 ^{1}H NMR spectra of compound **2n**

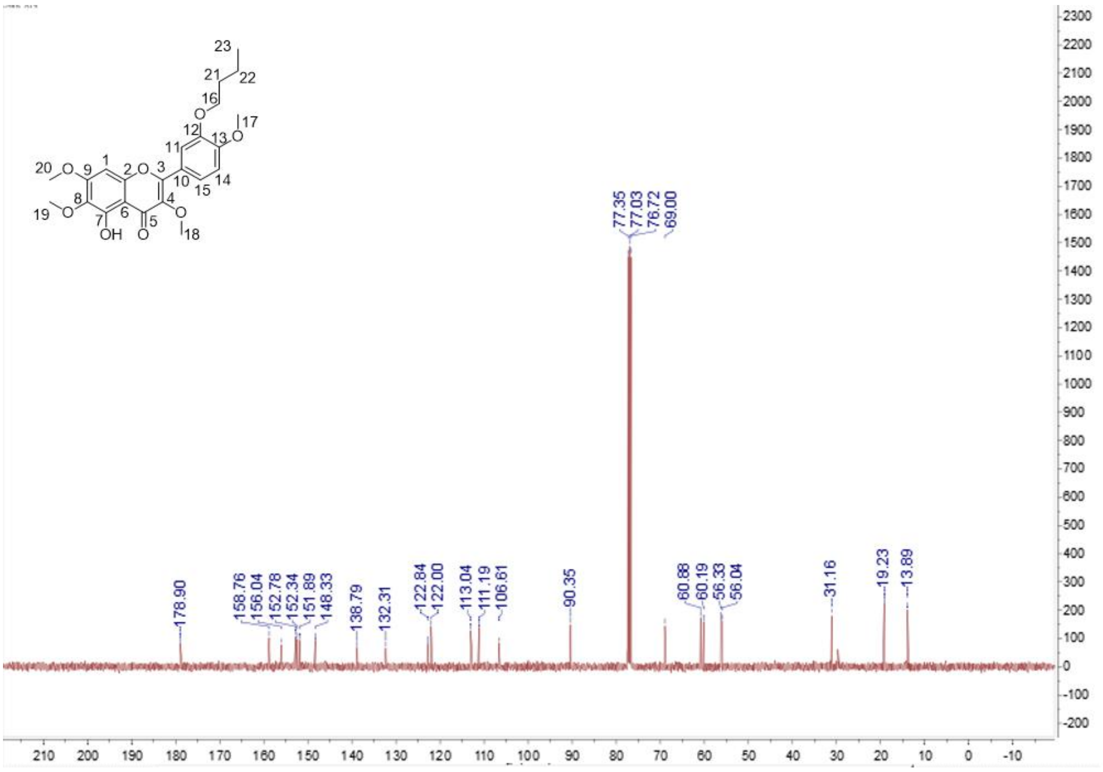

Figure S28 ^{13}C NMR spectra of compound **2n**

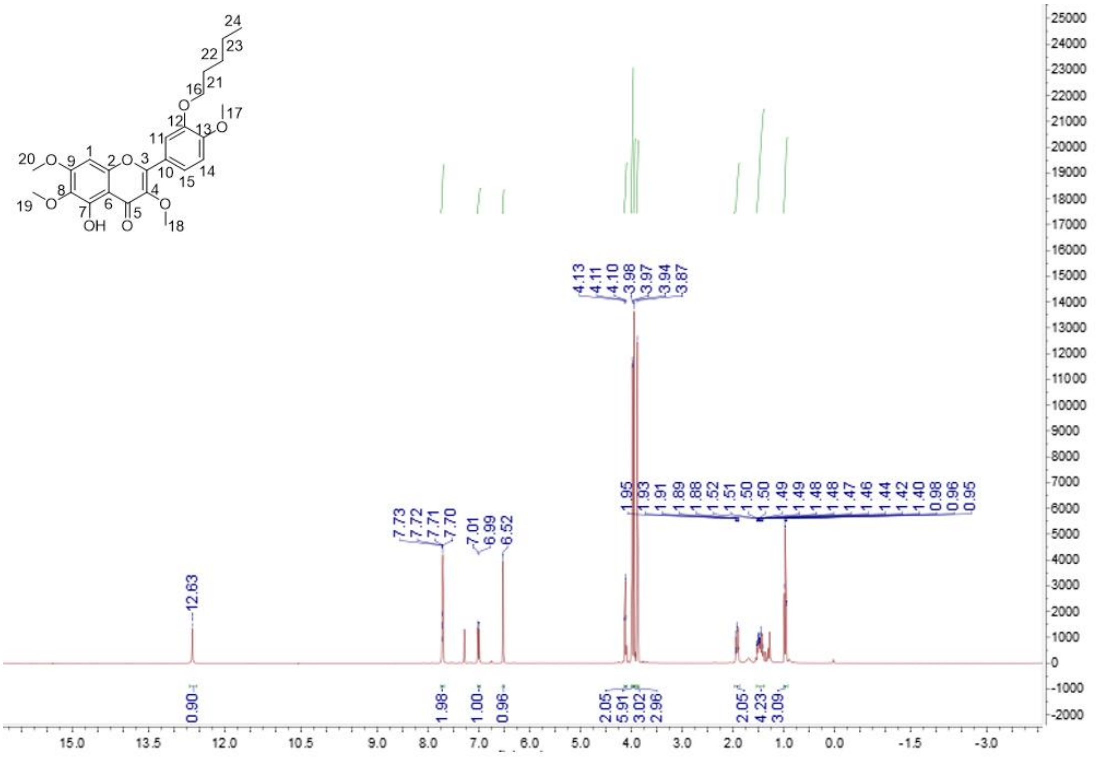

Figure S29 ^{1}H NMR spectra of compound **2o**

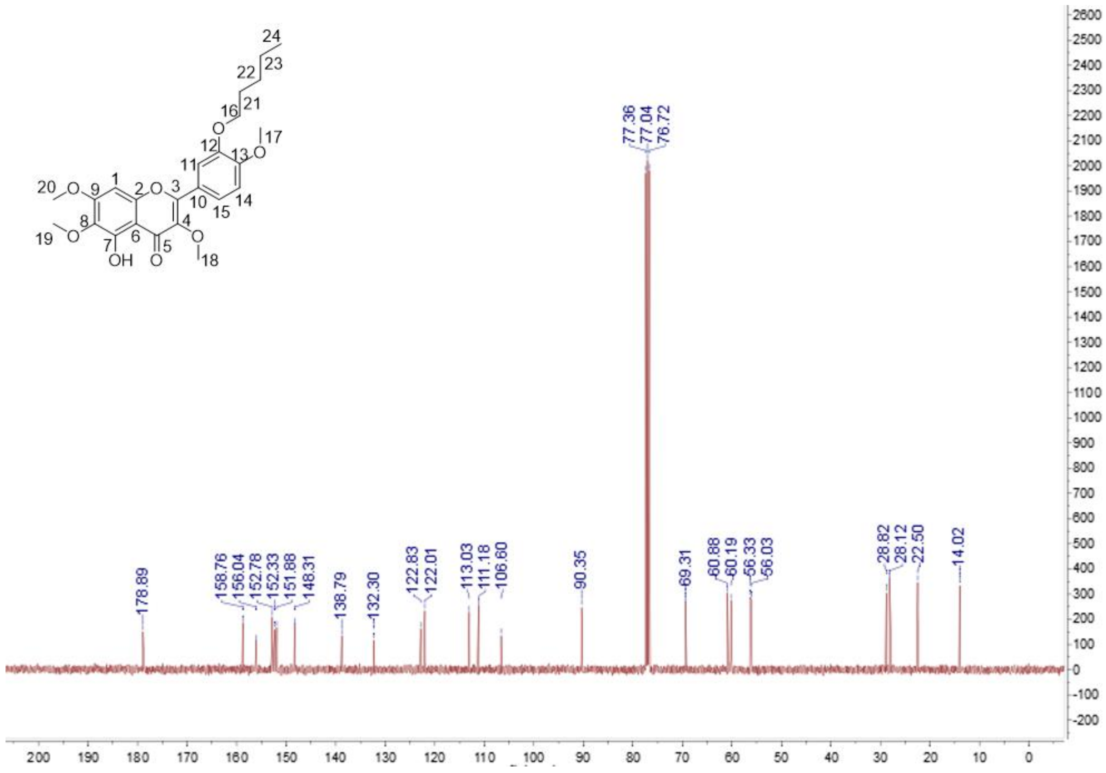

Figure S30 ^{13}C NMR spectra of compound **2o**

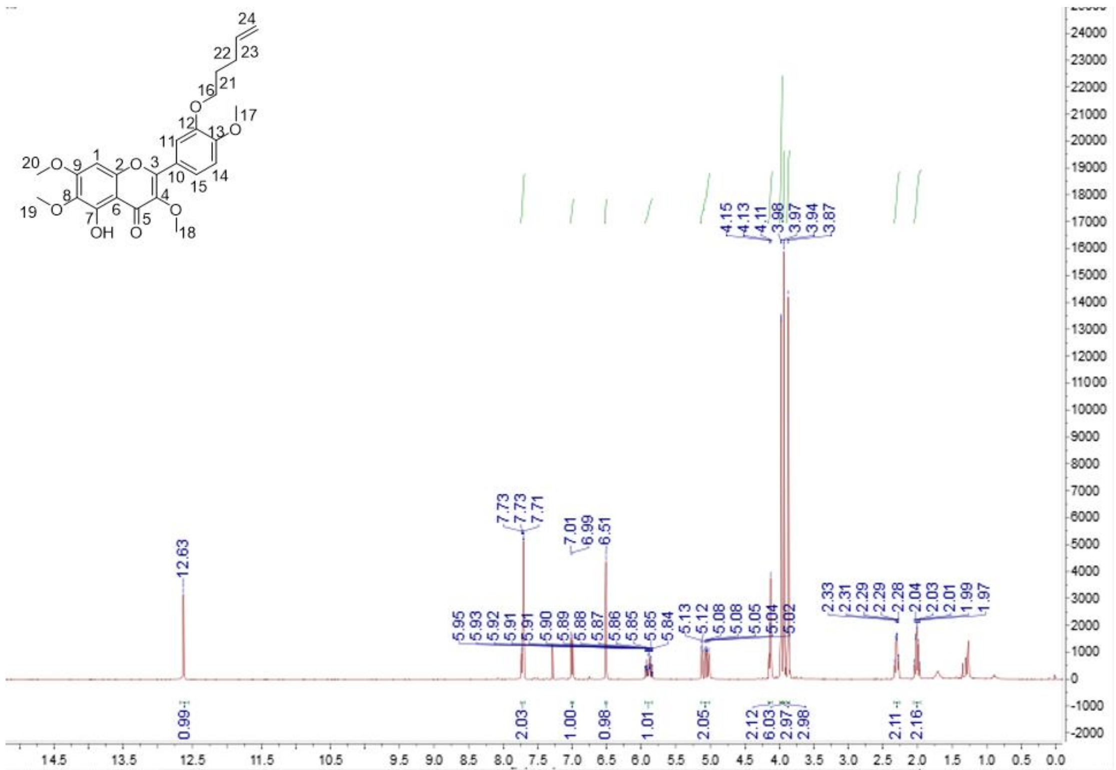

Figure S31 ^{1}H NMR spectra of compound **2p**

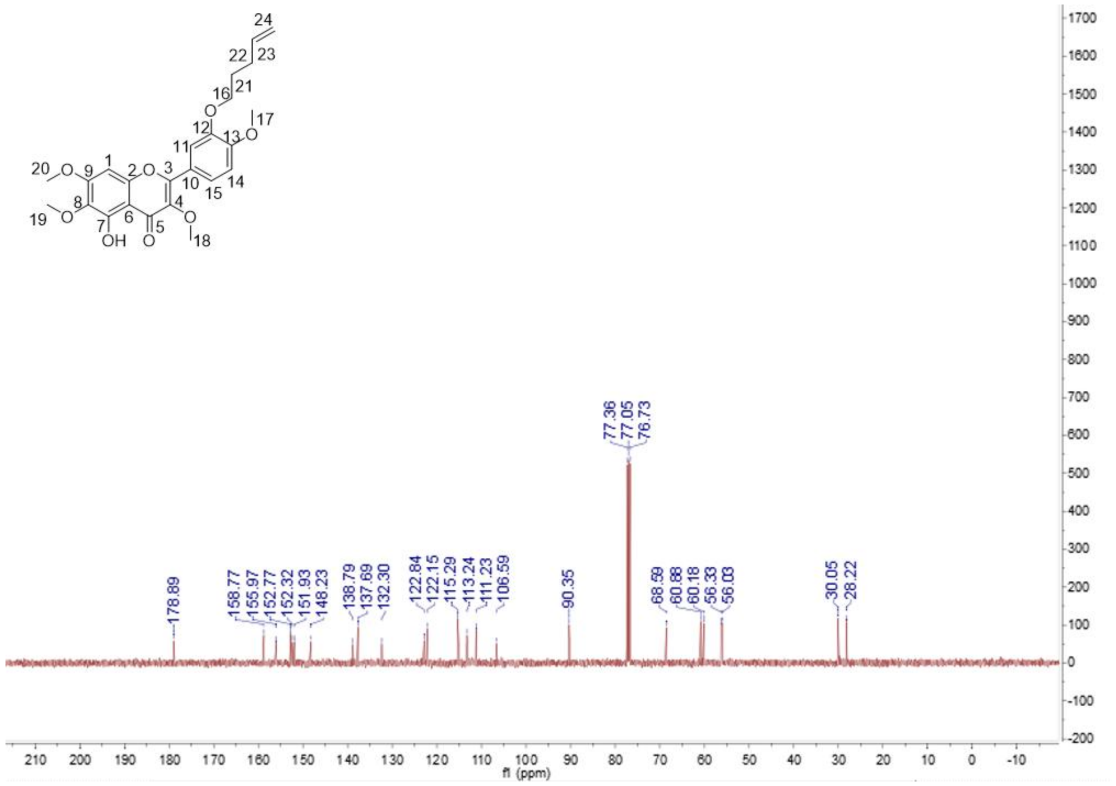

Figure S32 ^{13}C NMR spectra of compound **2p**

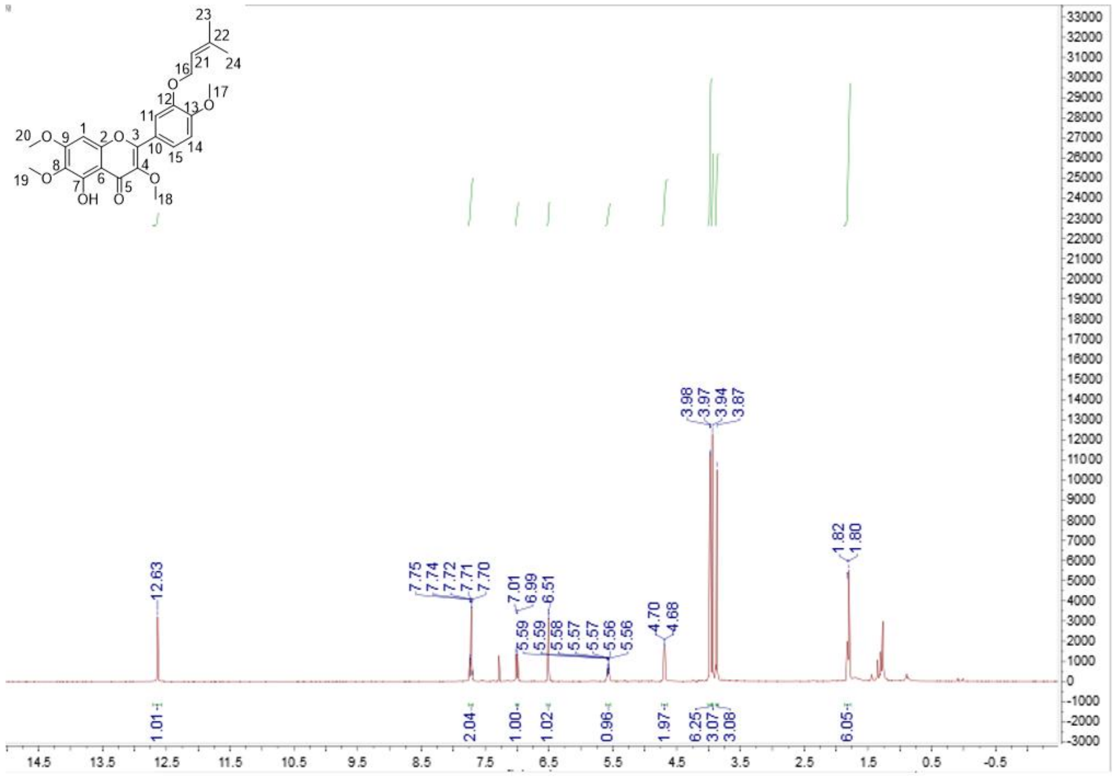

Figure S33 ^{1}H NMR spectra of compound **2q**

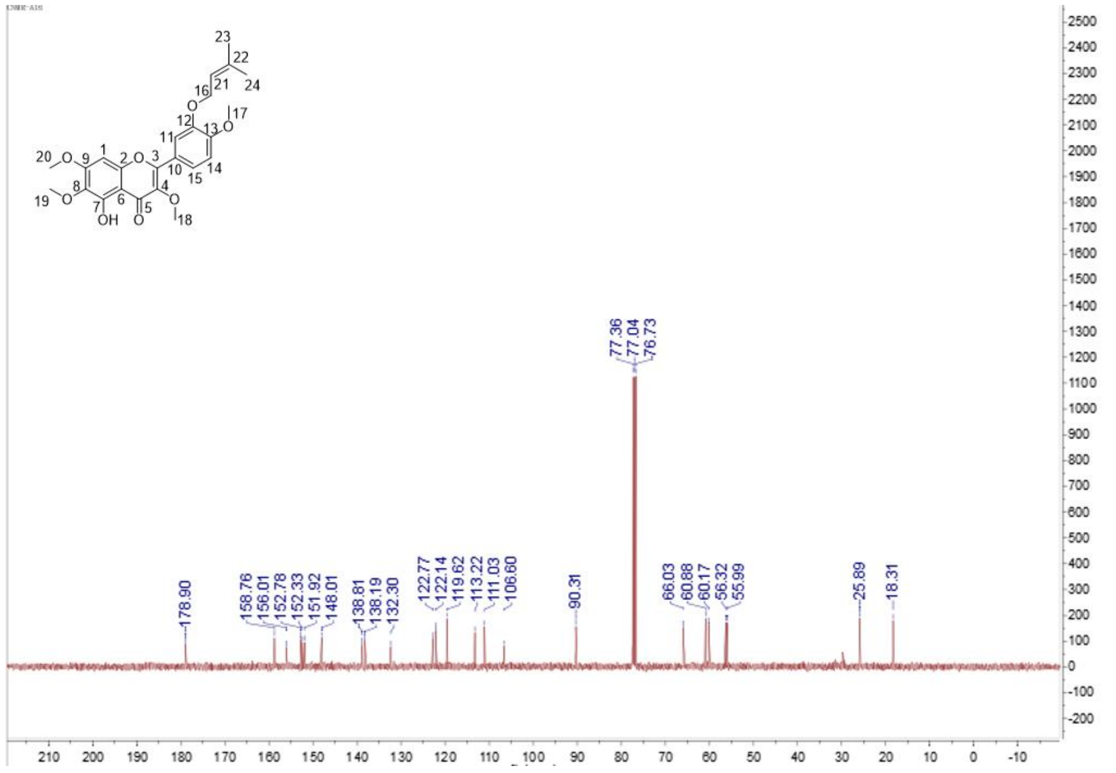

Figure S34 ^{13}C NMR spectra of compound **2q**

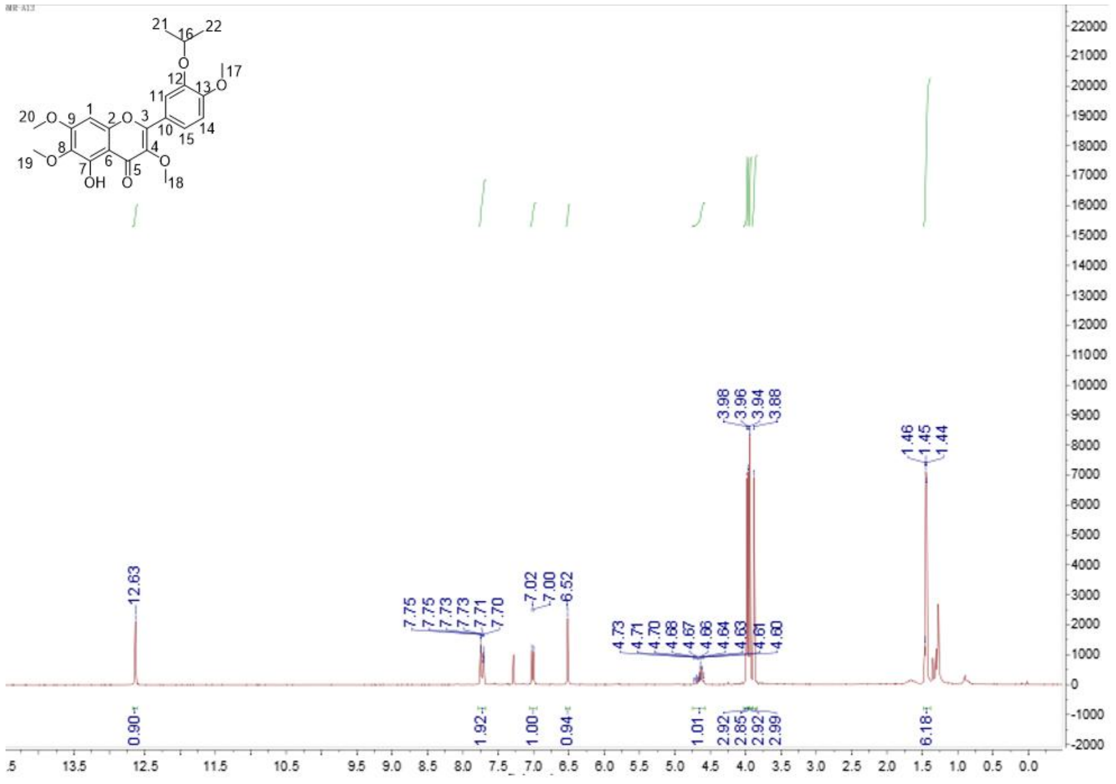

Figure S35 ^{1}H NMR spectra of compound **2r**

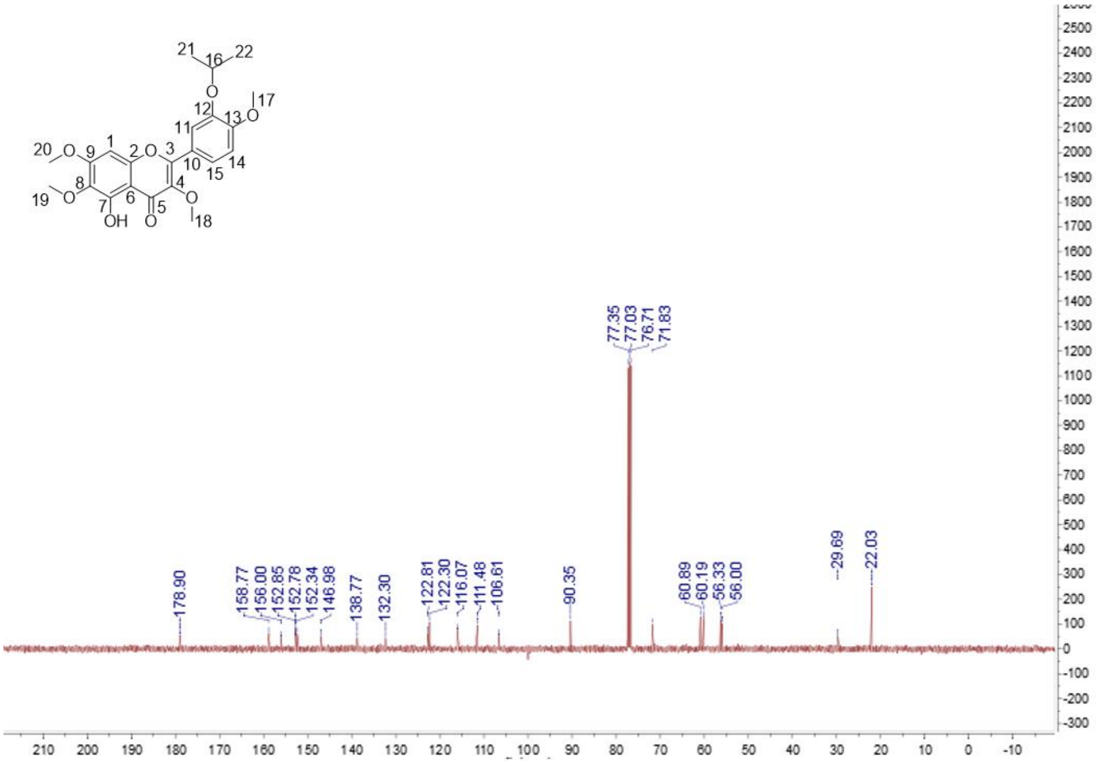

Figure S36 ^{13}C NMR spectra of compound **2r**

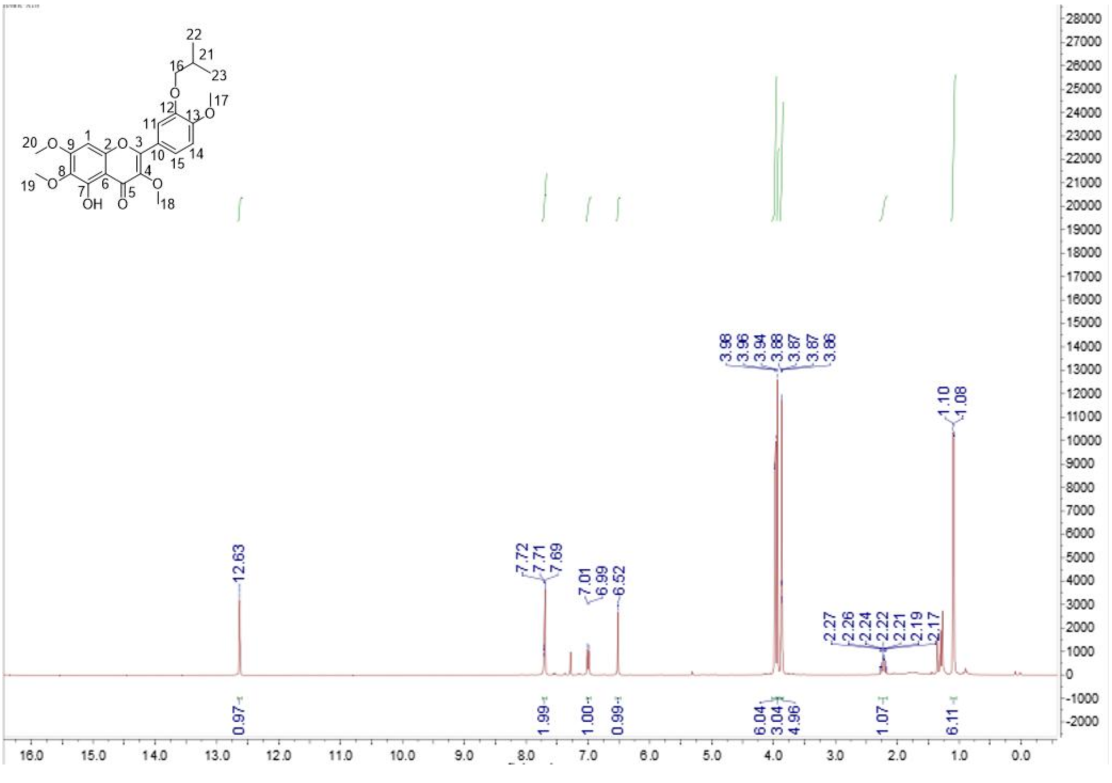

Figure S37 ^{1}H NMR spectra of compound **2s**

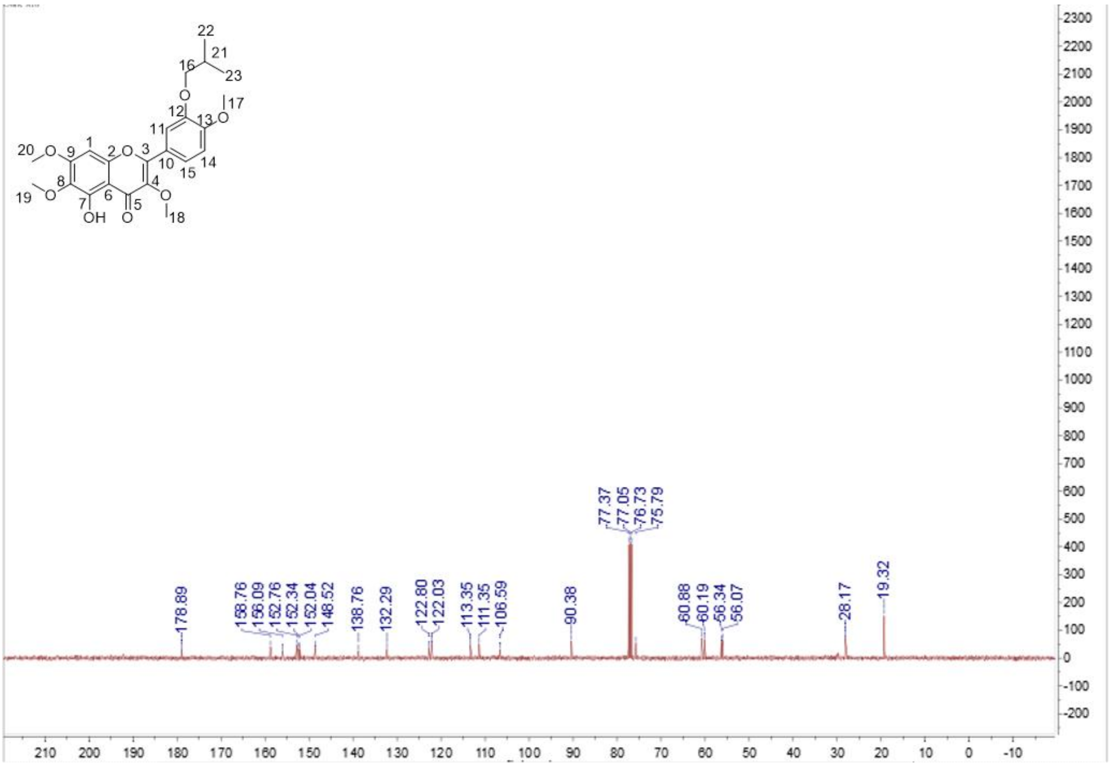

Figure S38 ^{13}C NMR spectra of compound **2s**

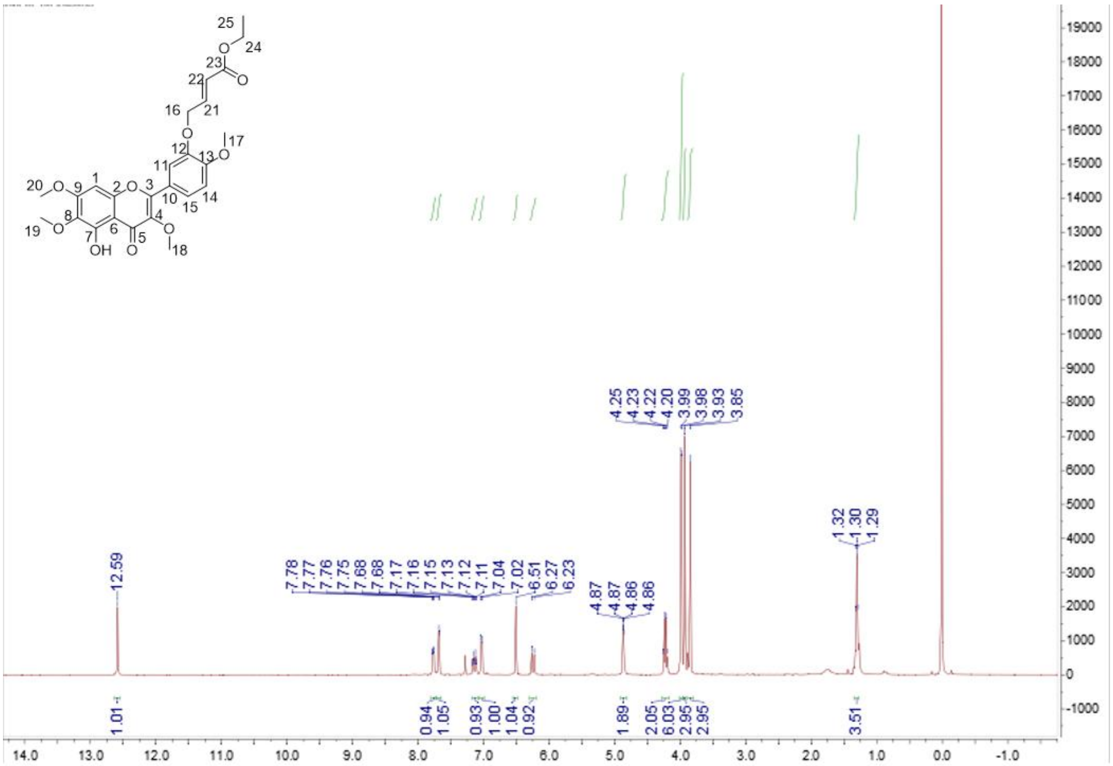

Figure S39 ^{1}H NMR spectra of compound **2t**

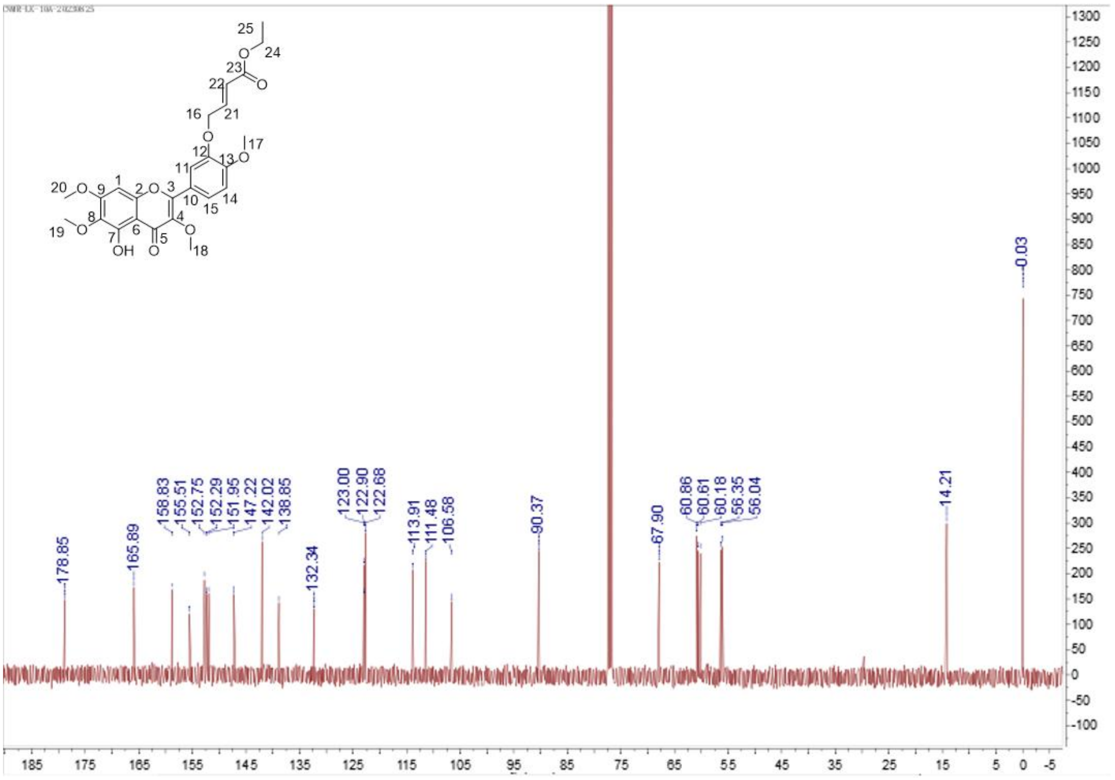

Figure S40 ^{13}C NMR spectra of compound **2t**

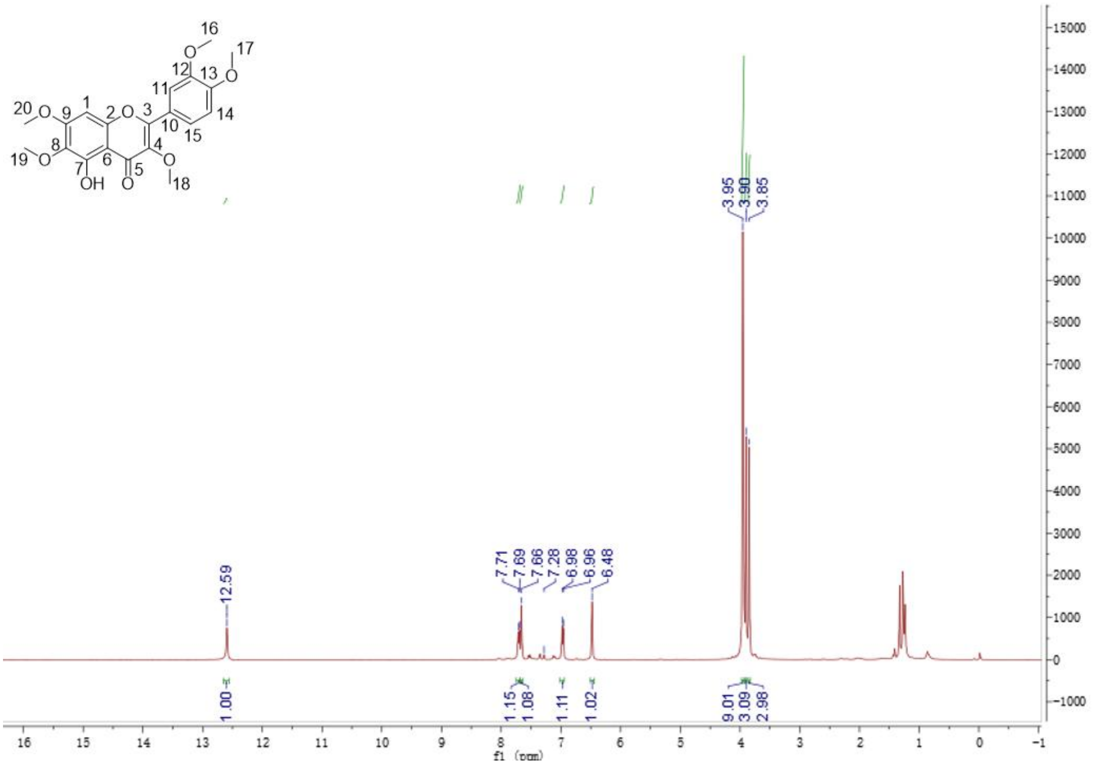

Figure S41 ^{1}H NMR spectra of compound **3a**

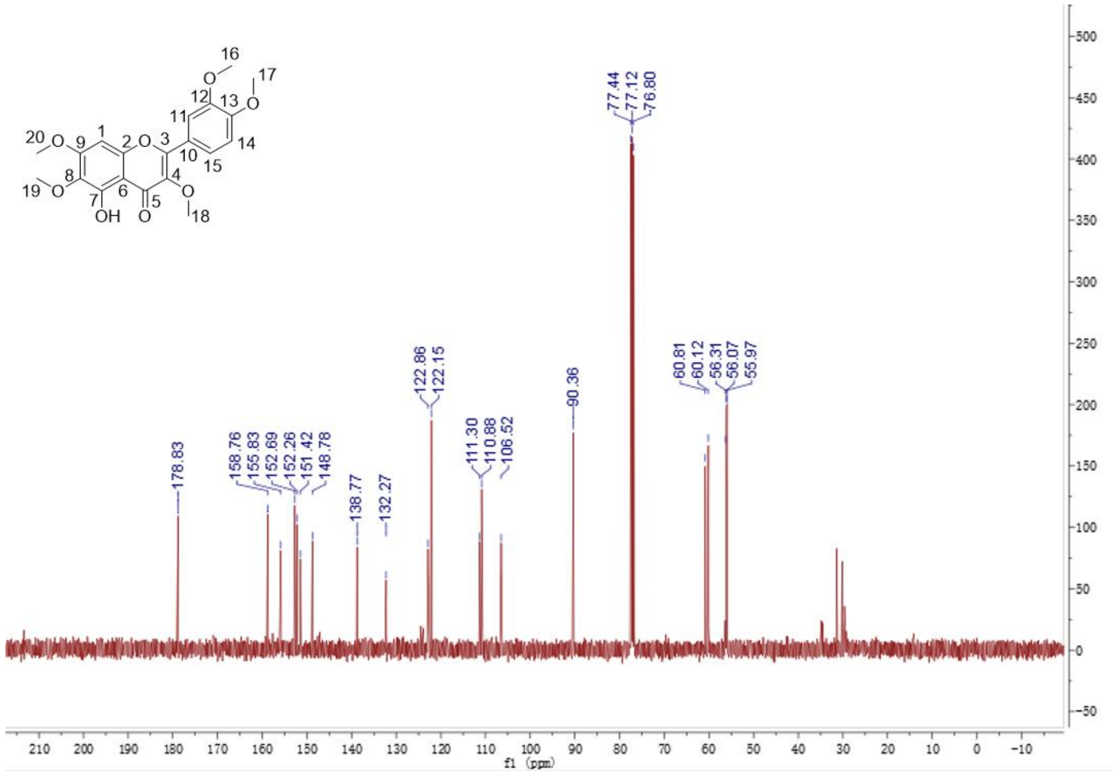

Figure S42 ^{13}C NMR spectra of compound **3a**

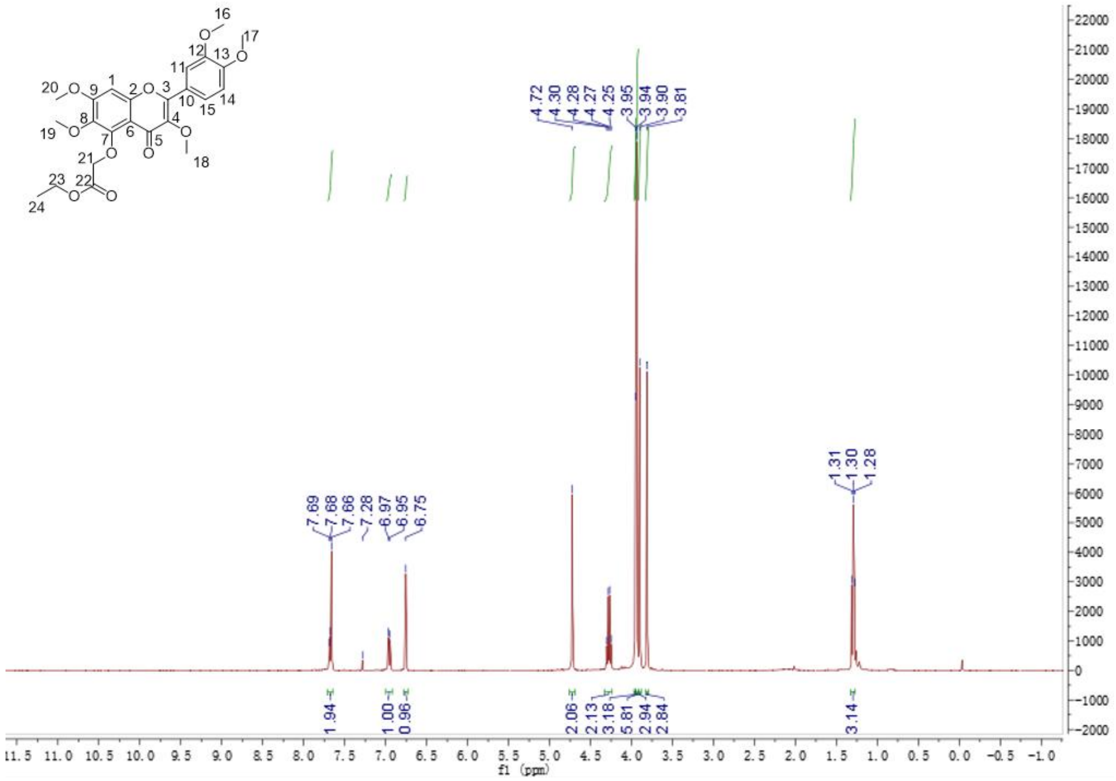

Figure S42 ^{1}H NMR spectra of compound **3b**

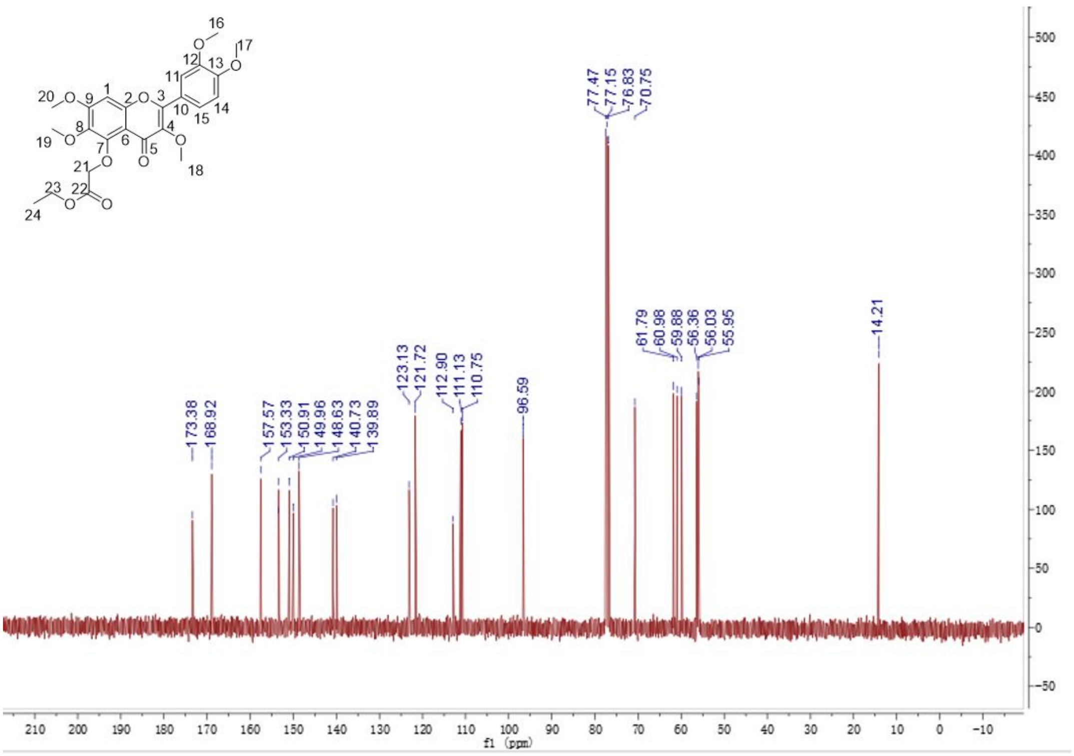

Figure S44 ^{13}C NMR spectra of compound **3b**

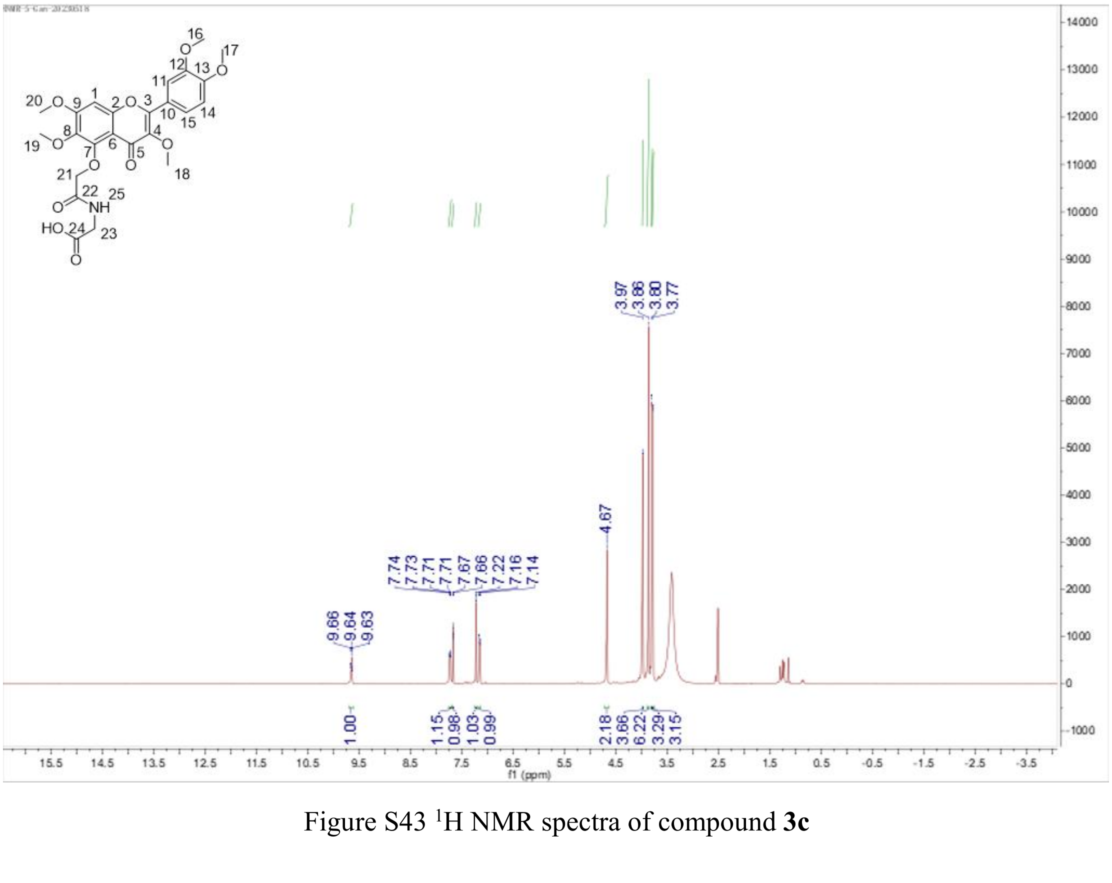

Figure S43 ^{1}H NMR spectra of compound **3c**

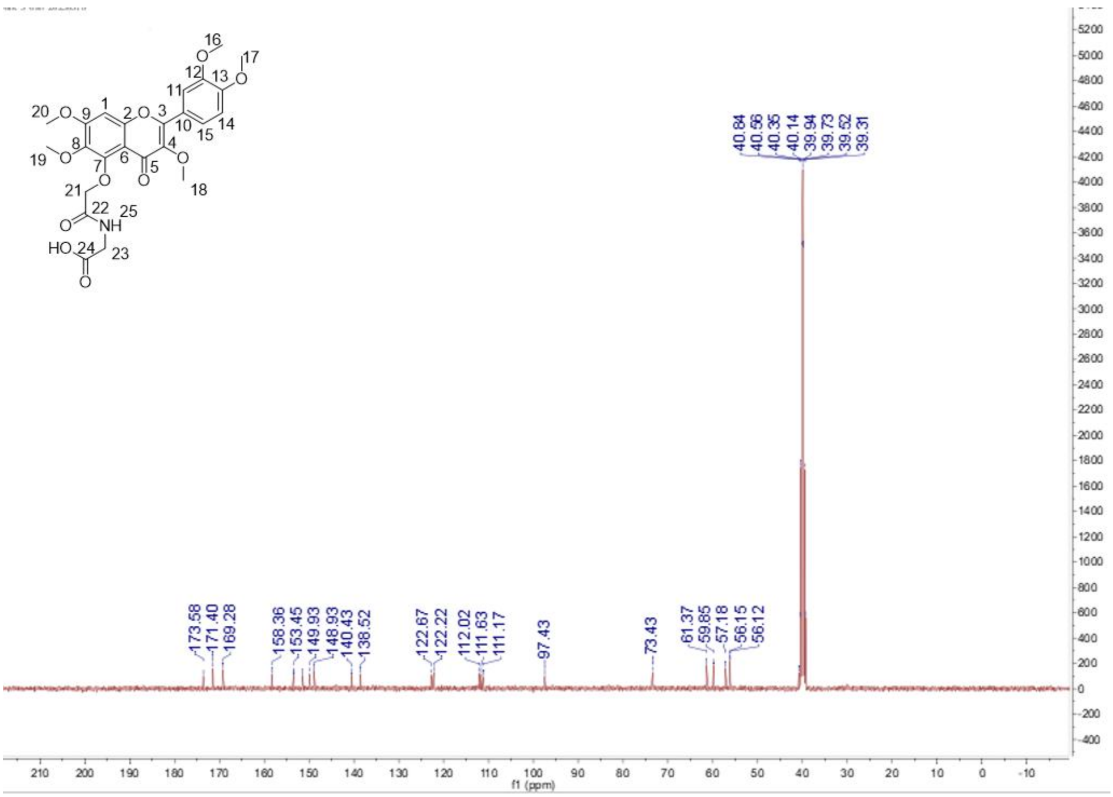

Figure S46 ^{13}C NMR spectra of compound **3c**

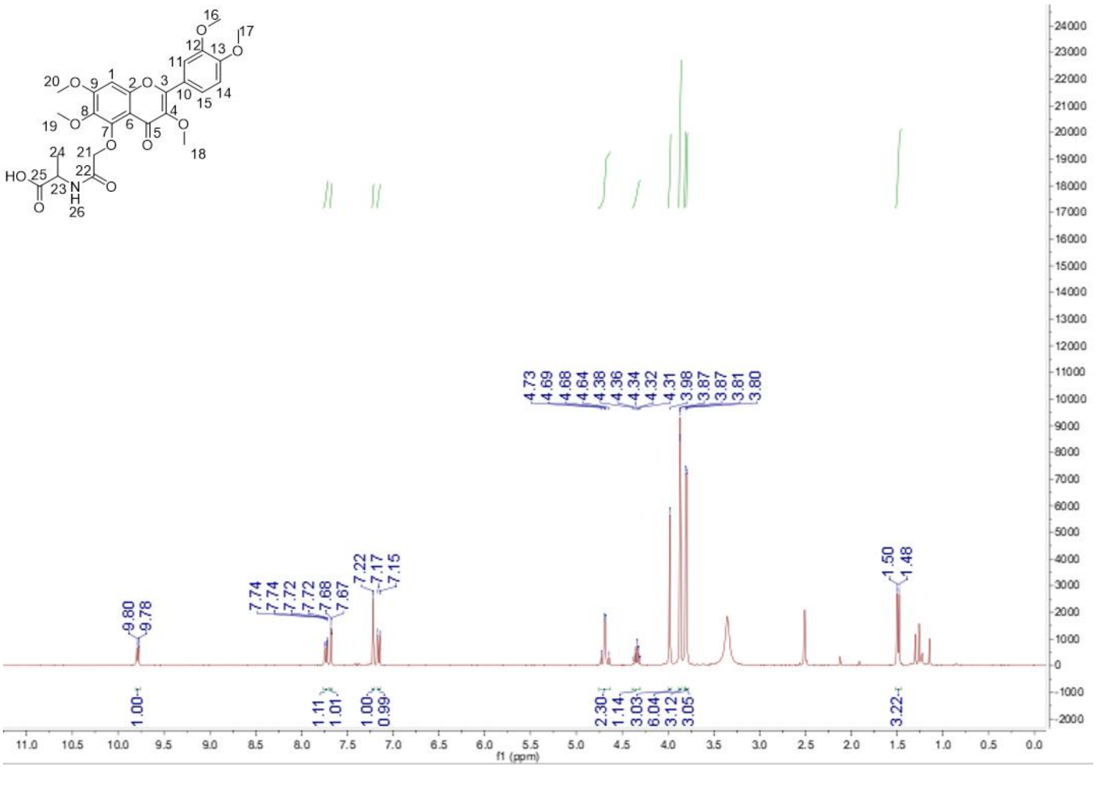

Figure S45 ^{1}H NMR spectra of compound **3d**

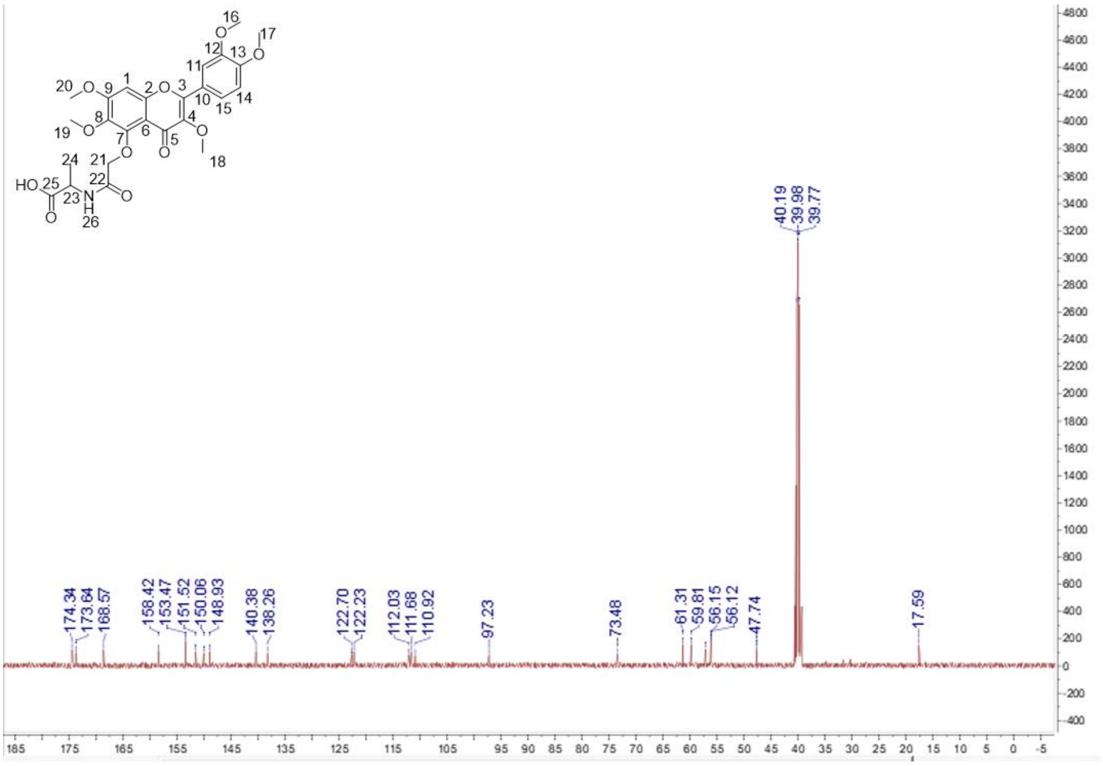

Figure S48 ^{13}C NMR spectra of compound **3d**

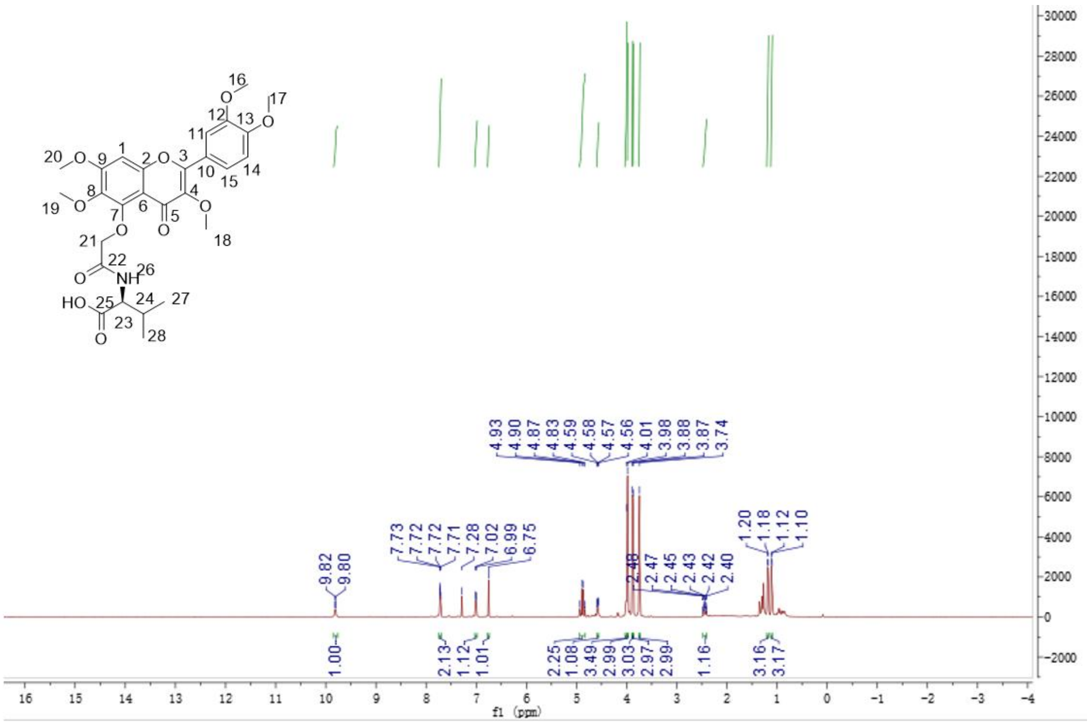

Figure S47 ^{1}H NMR spectra of compound **3e**

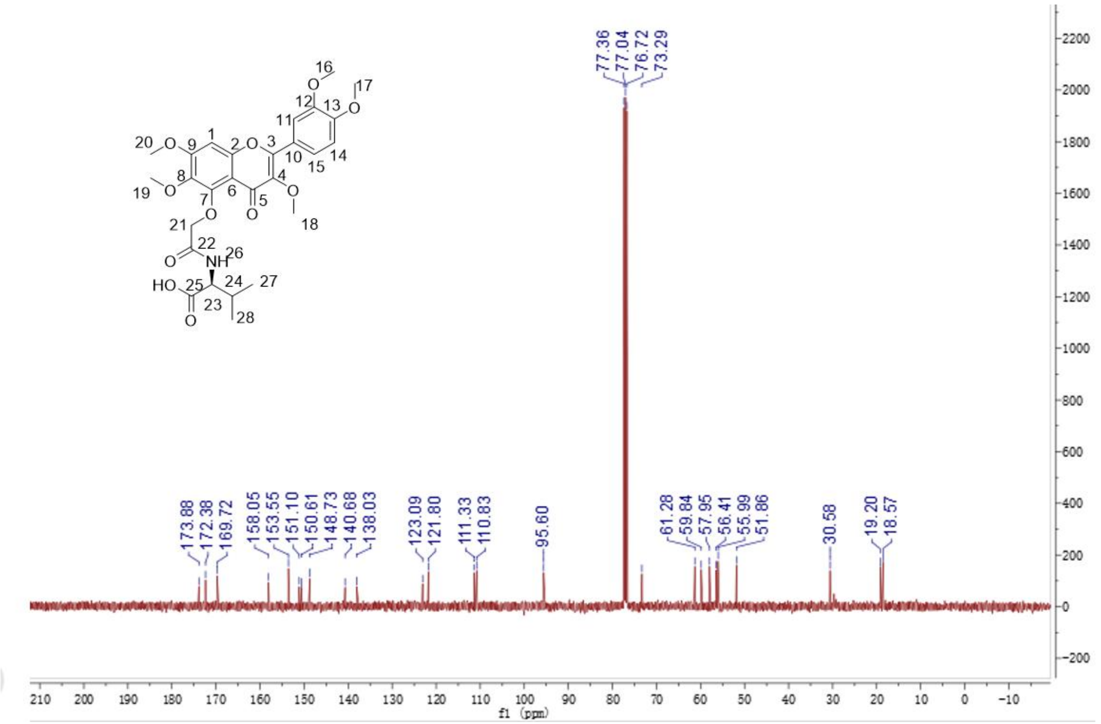

Figure S48 ^{13}C NMR spectra of compound **3e**

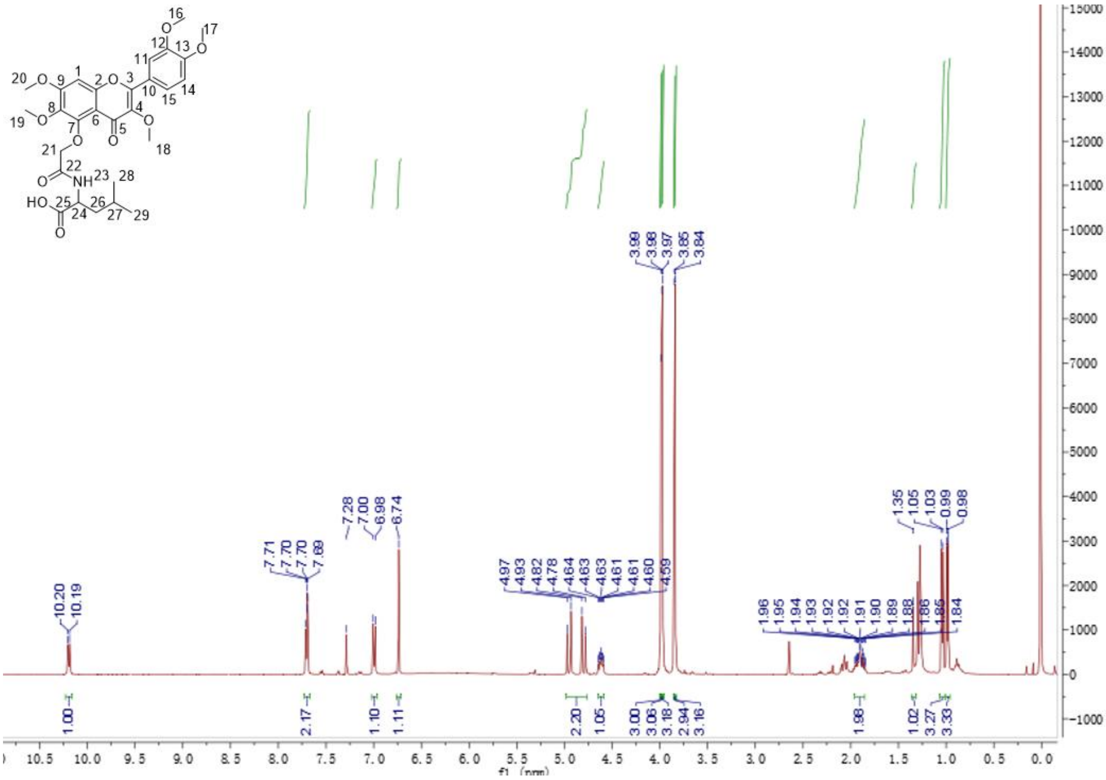

Figure S49 ^{1}H NMR spectra of compound **3f**

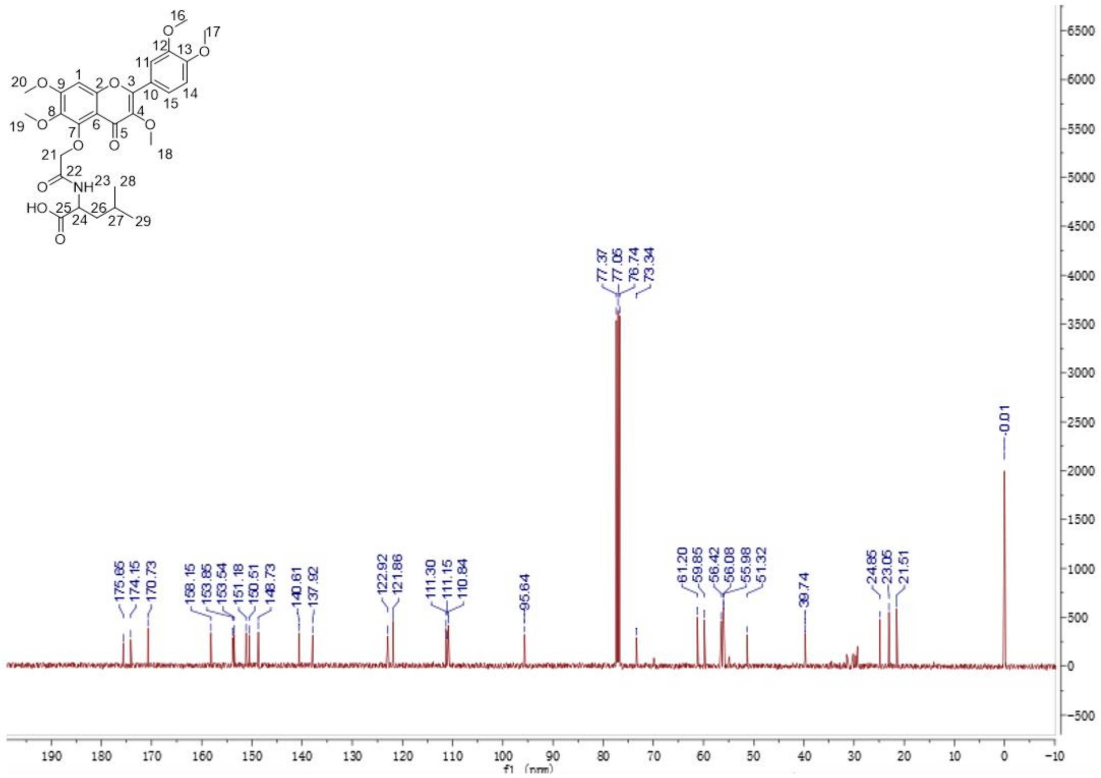

Figure S50 ^{13}C NMR spectra of compound **3f**

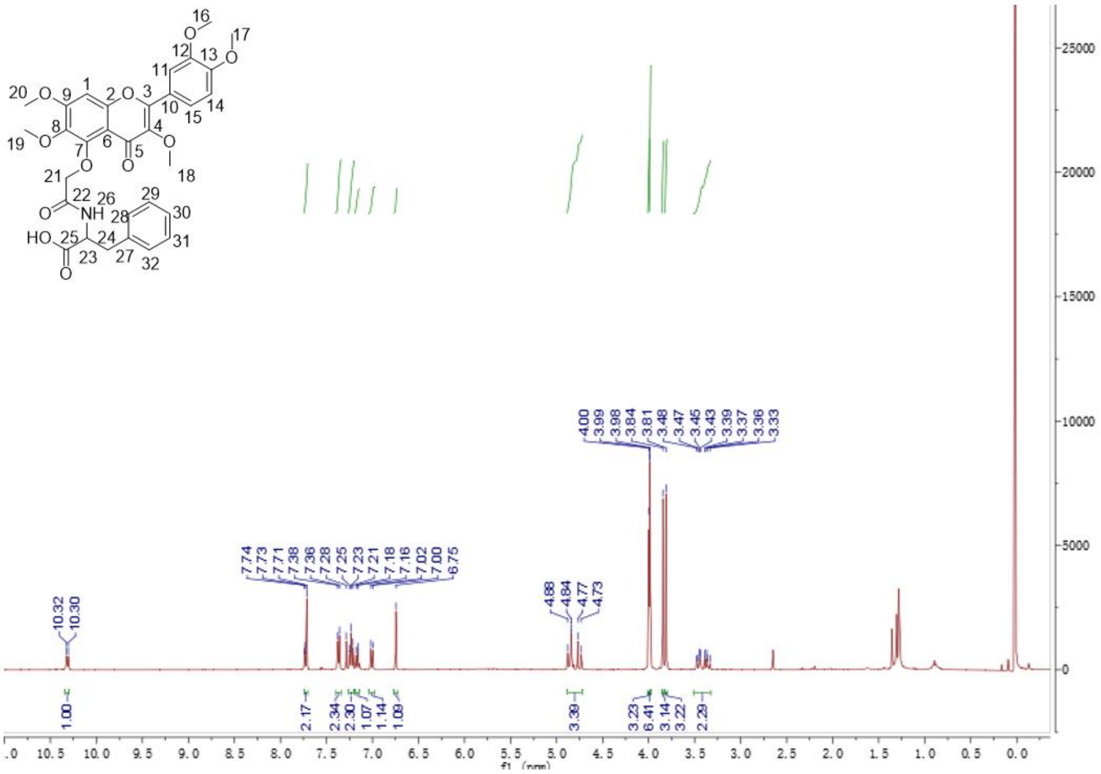

Figure S51 ^{1}H NMR spectra of compound **3g**

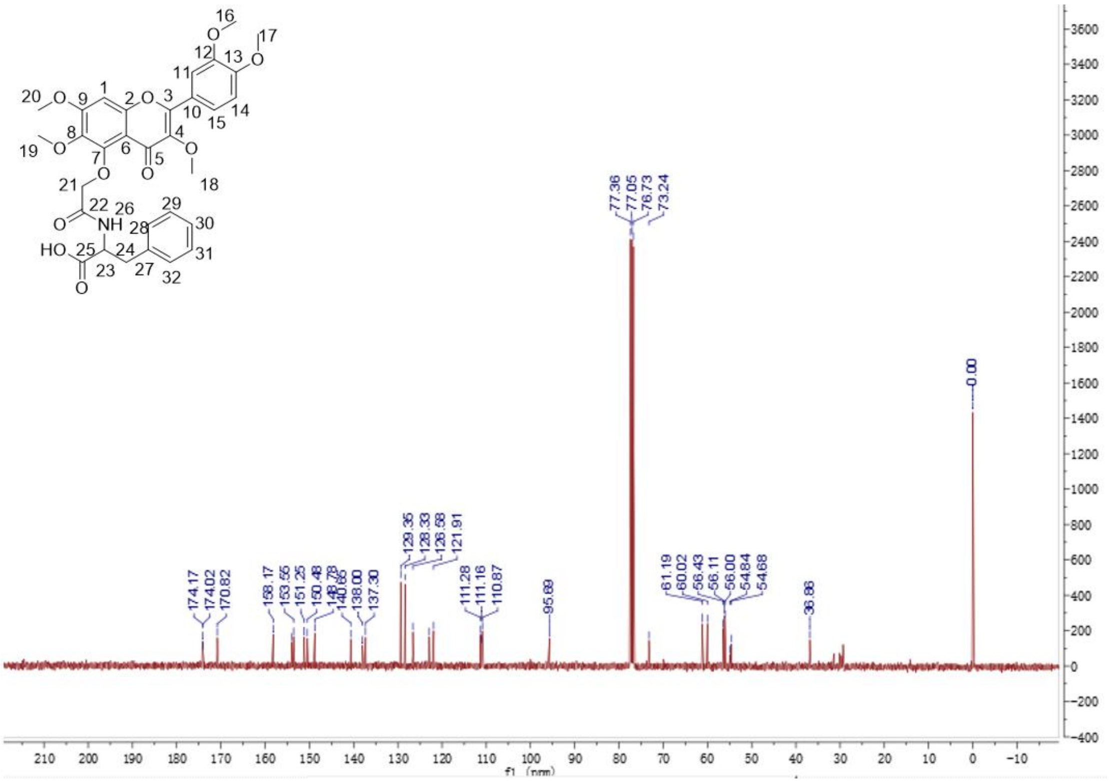

Figure S52 ^{13}C NMR spectra of compound **3g**

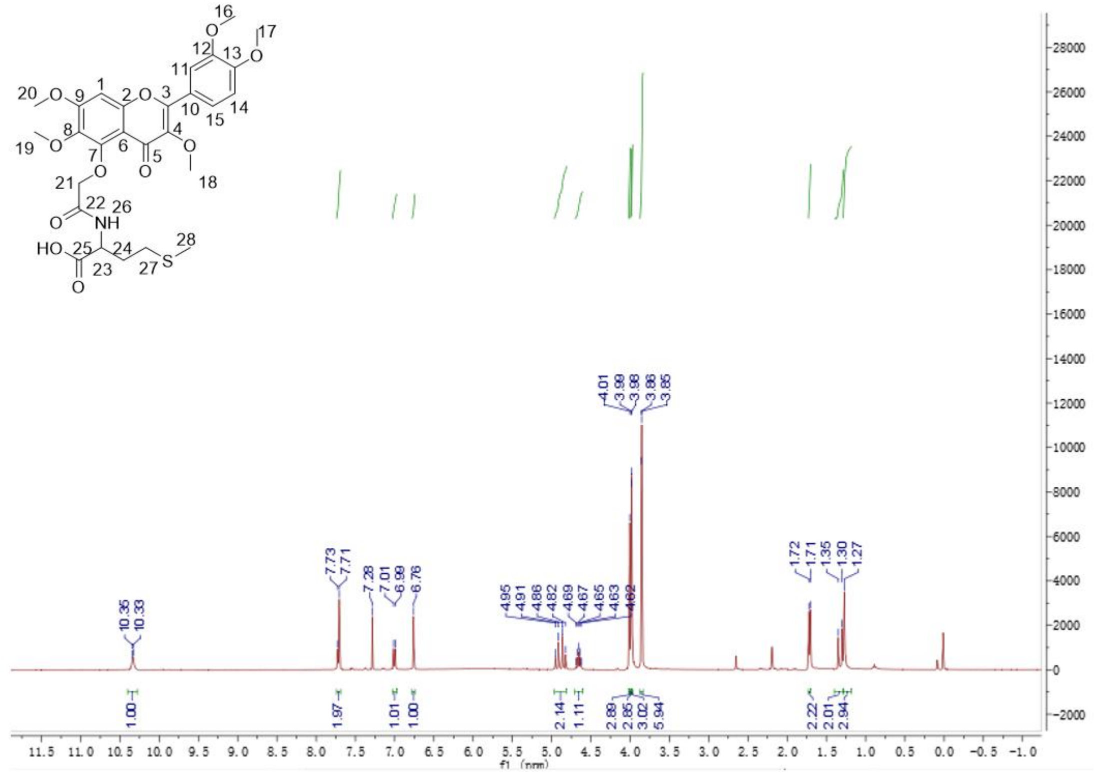

Figure S53 ^{1}H NMR spectra of compound **3h**

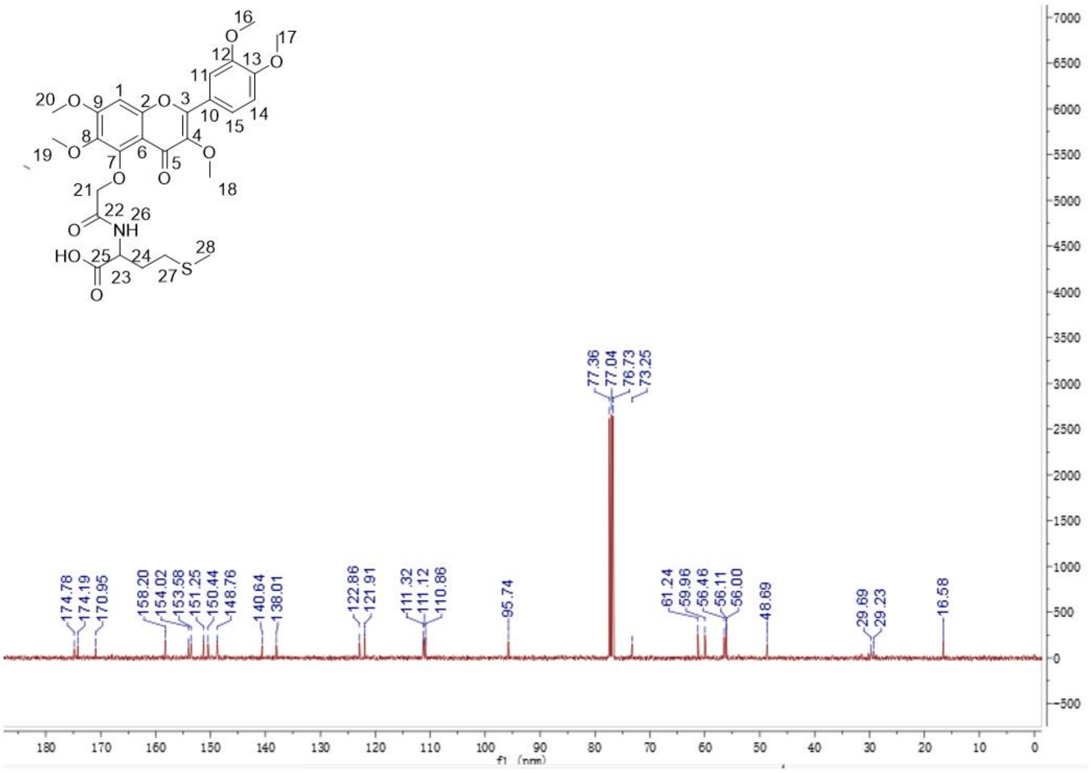

Figure S54 ^{13}C NMR spectra of compound **3h**

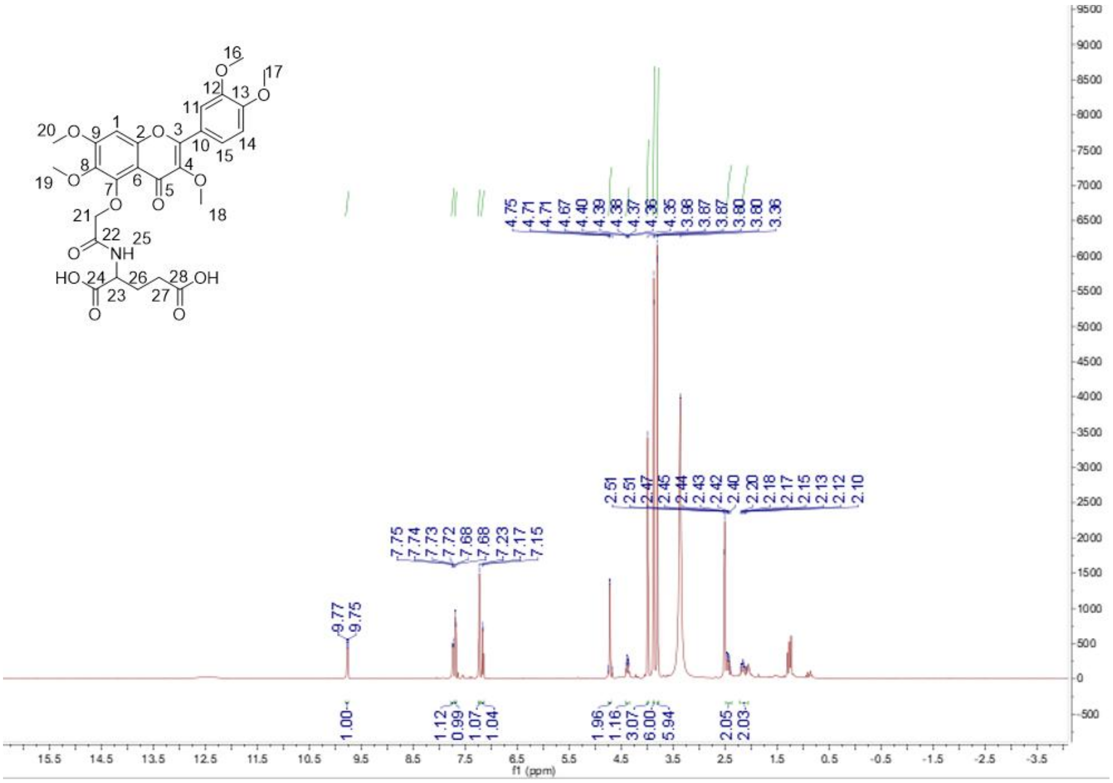

Figure S55 ^{1}H NMR spectra of compound **3i**

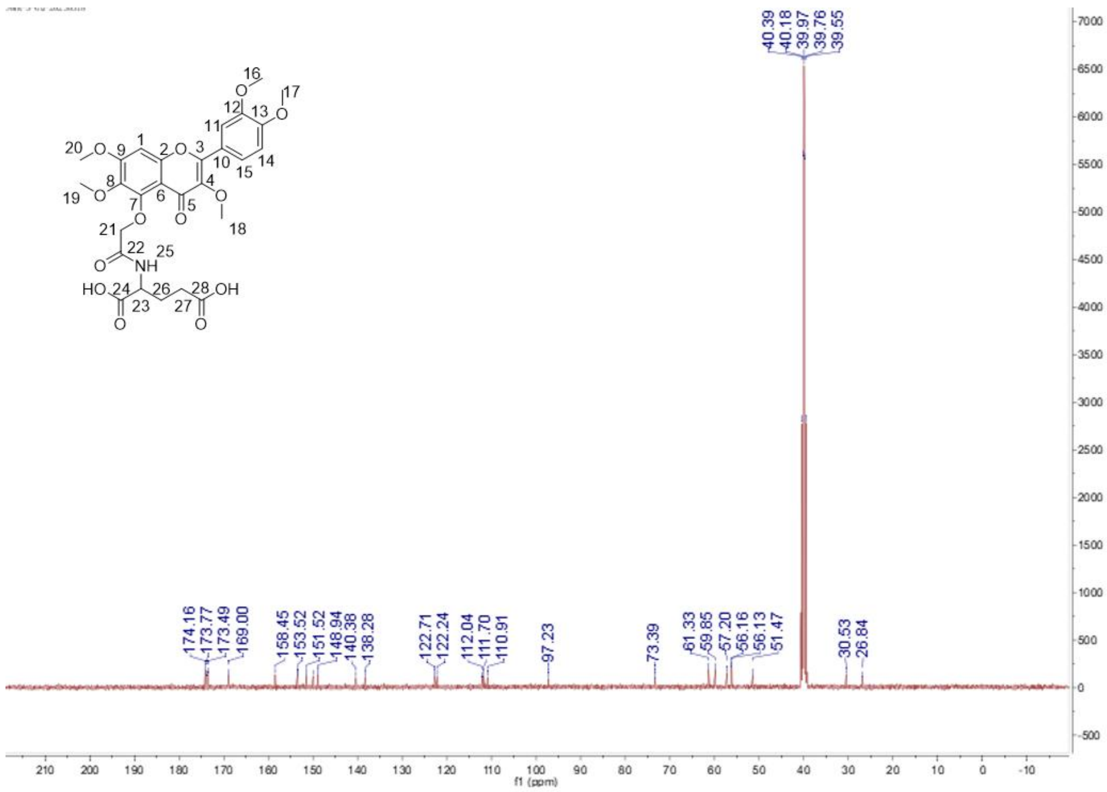

Figure S56 ^{13}C NMR spectra of compound **3i**

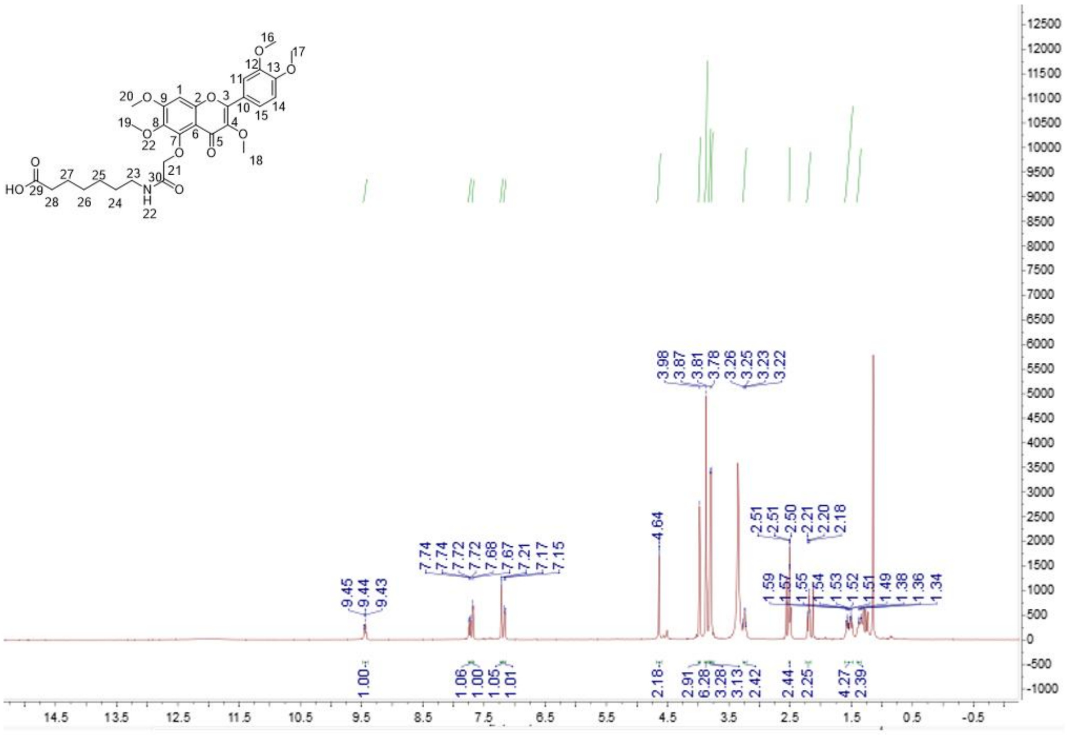

Figure S57 ^{1}H NMR spectra of compound **3j**

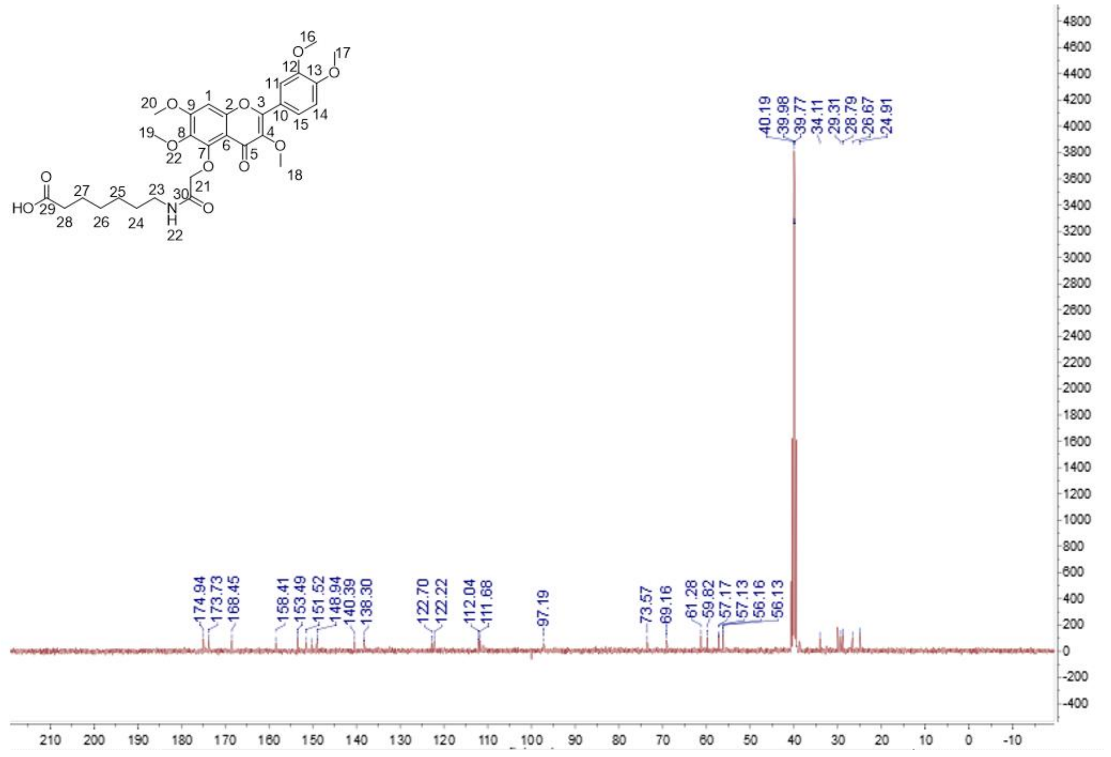

Figure S58 ^{13}C NMR spectra of compound **3j**

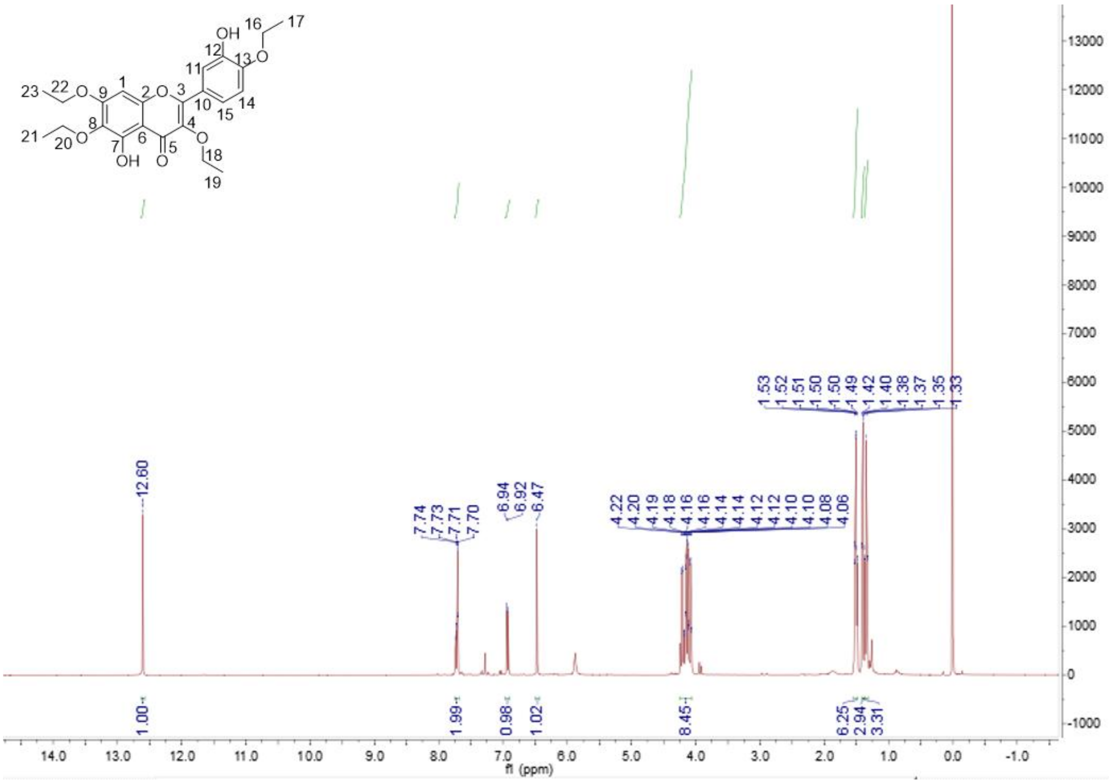

Figure S59 ^{1}H NMR spectra of compound **4a**

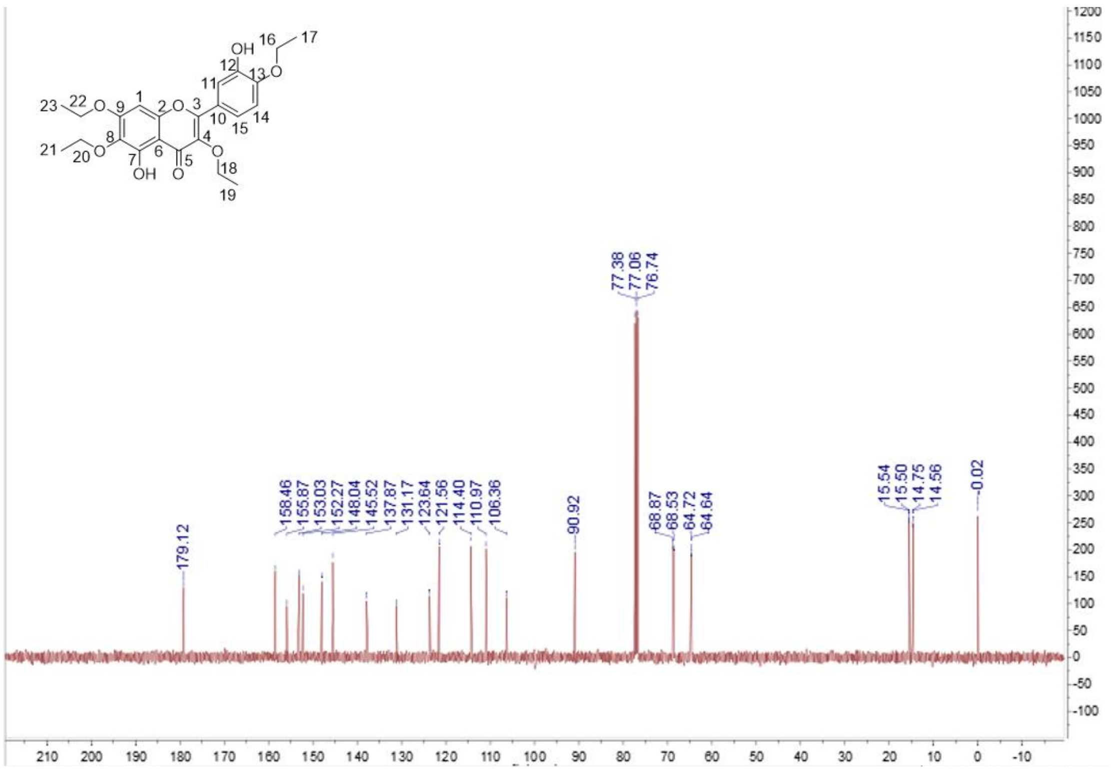

Figure S60 ^{13}C NMR spectra of compound **4a**

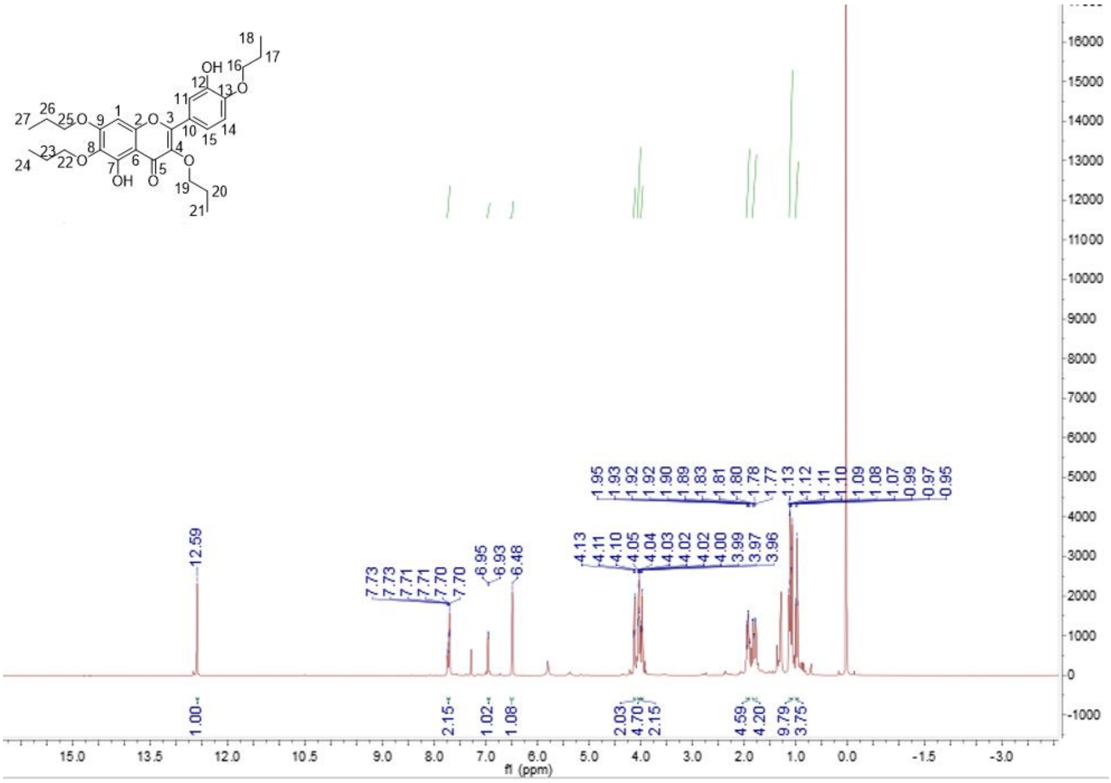

Figure S61 ^{1}H NMR spectra of compound **4b**

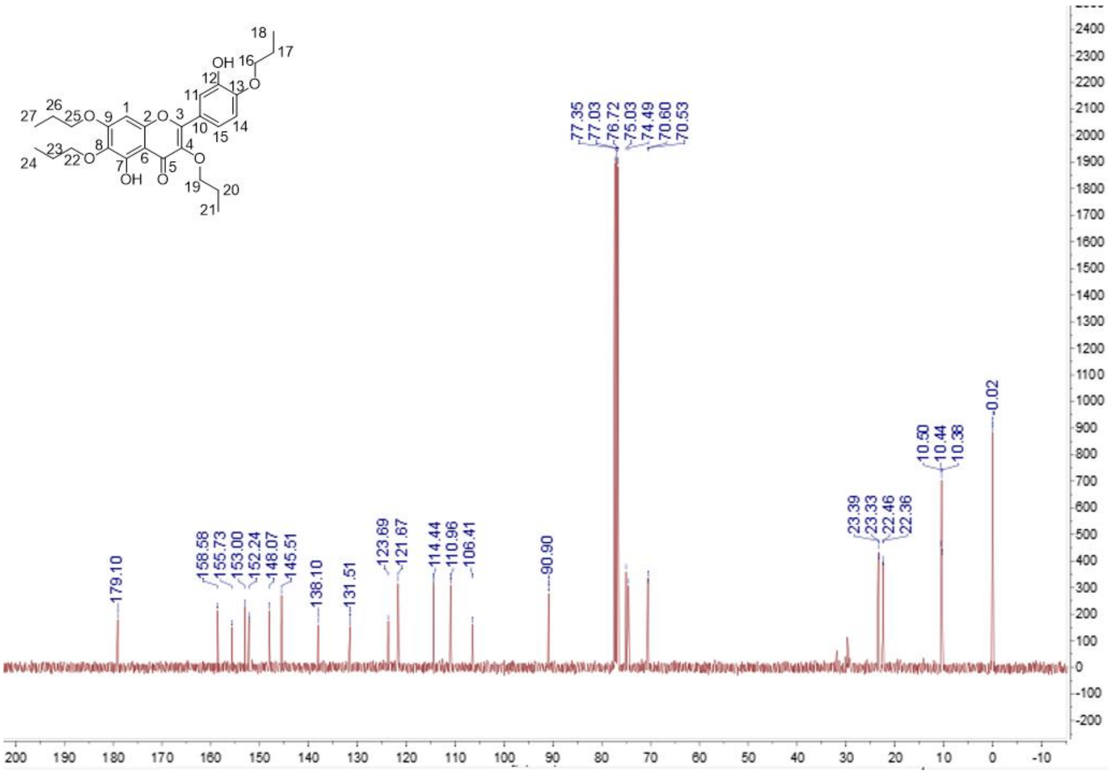

Figure S62 ^{13}C NMR spectra of compound **4b**

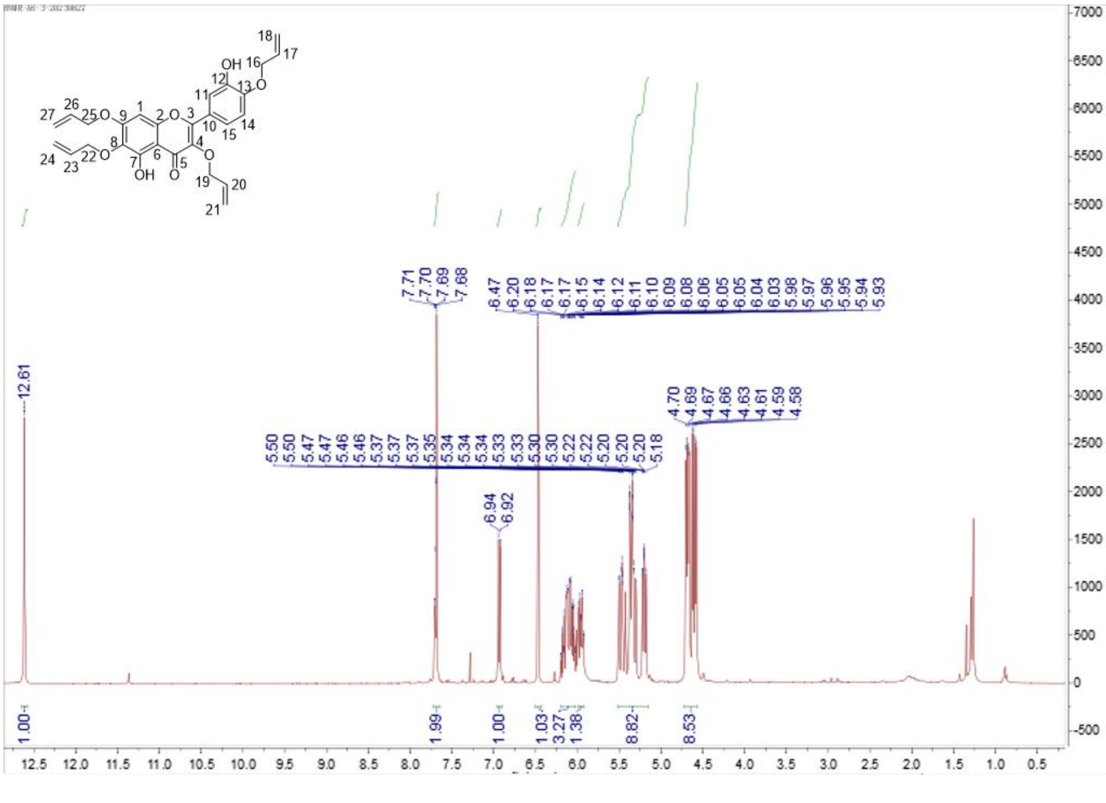

Figure S63 ^{1}H NMR spectra of compound **4c**

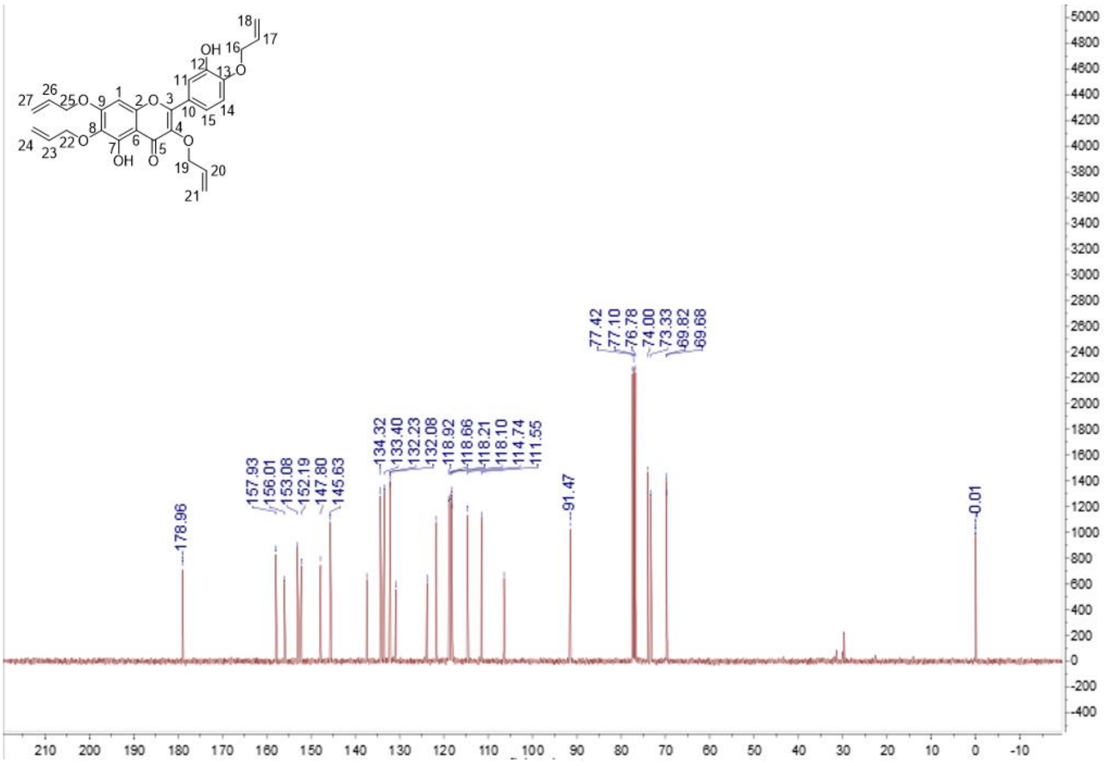

Figure S64 ^{13}C NMR spectra of compound **4c**

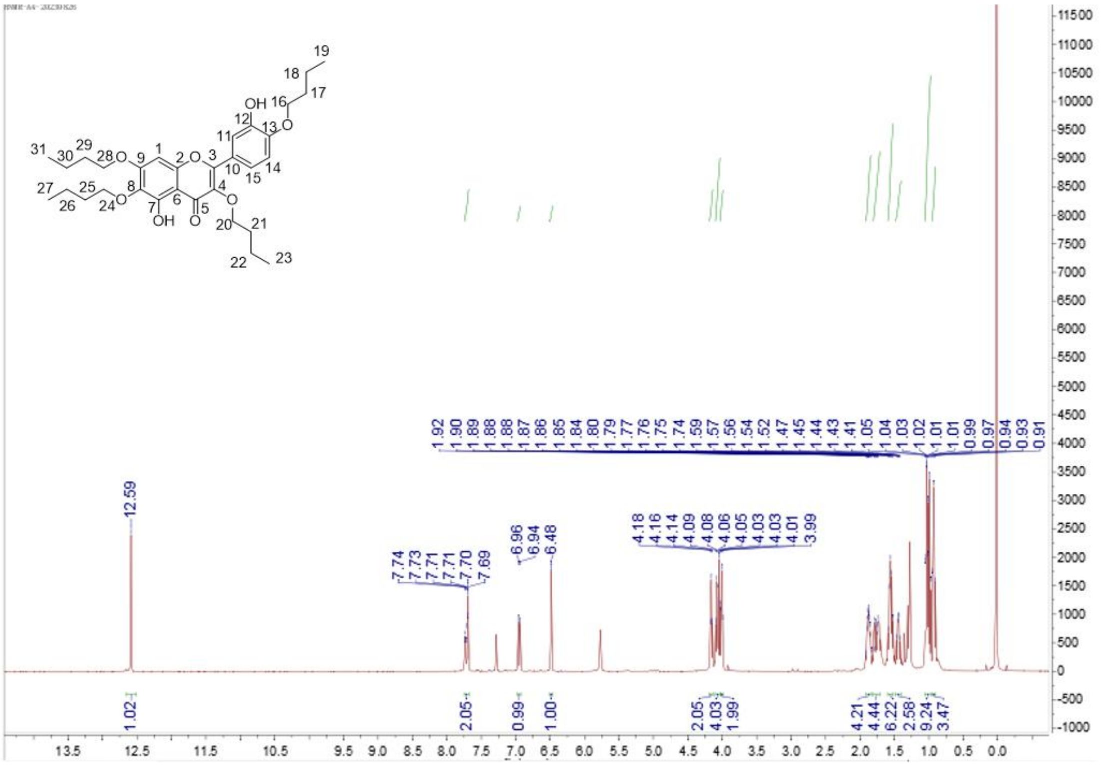

Figure S65 ^{1}H NMR spectra of compound **4d**

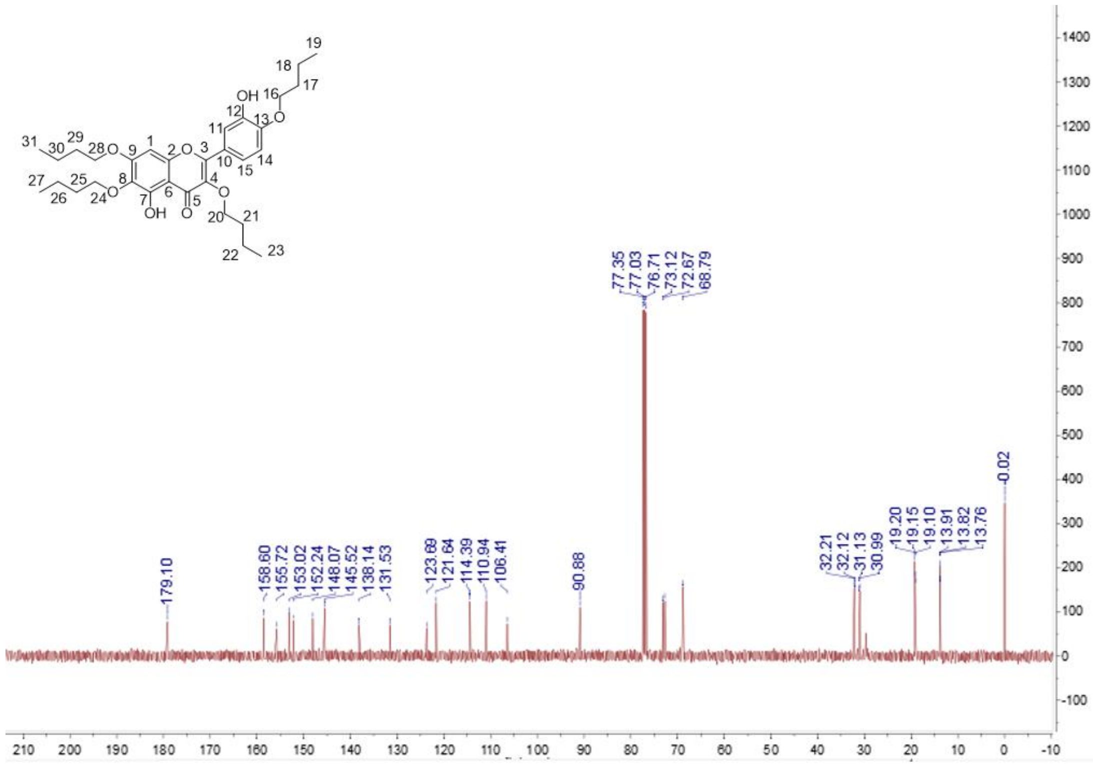

Figure S66 ^{13}C NMR spectra of compound **4d**

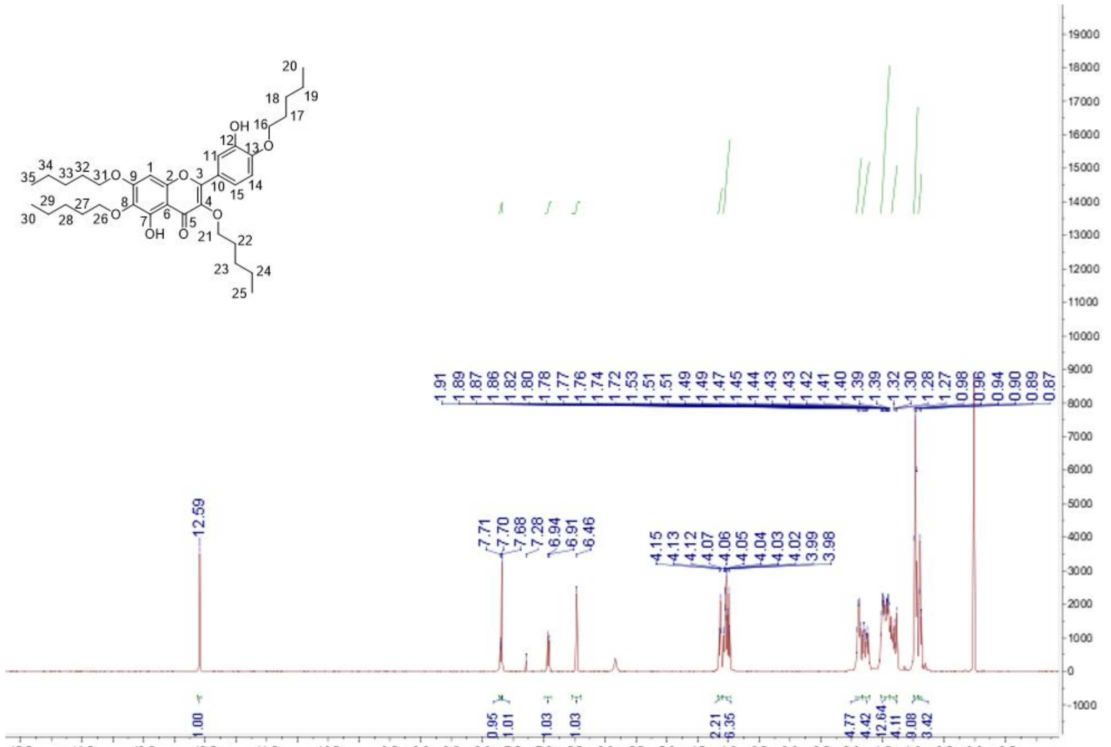

Figure S67 ^{1}H NMR spectra of compound **4e**

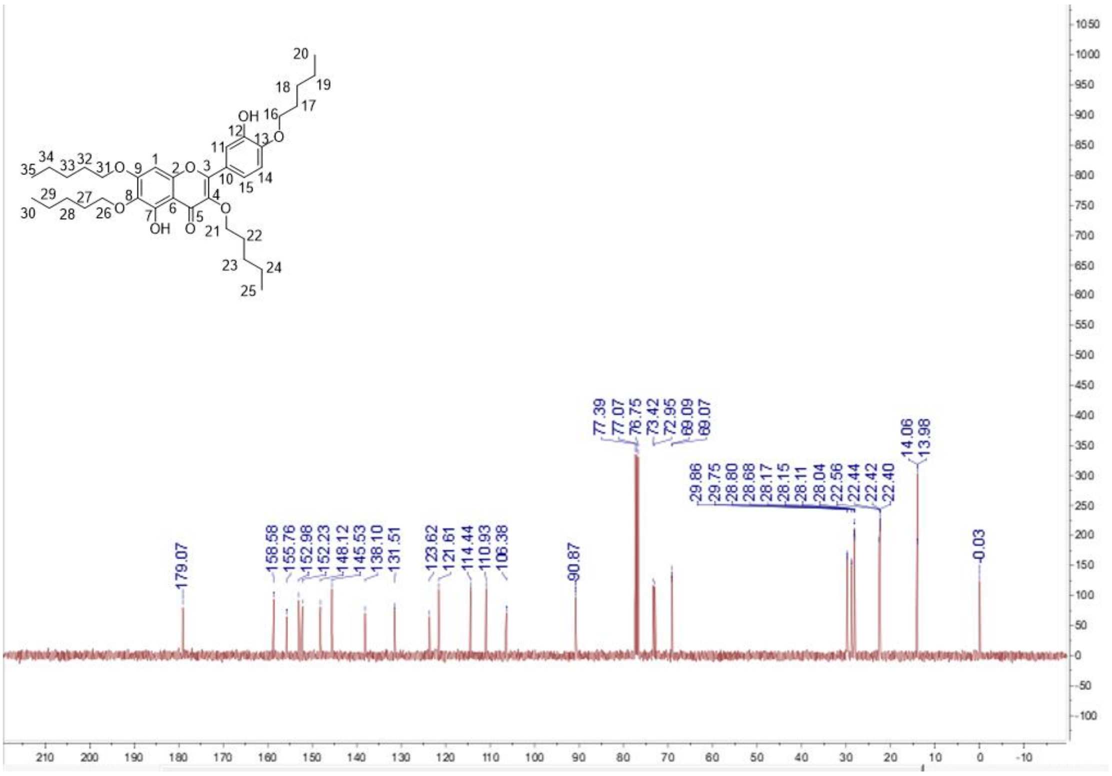

Figure S68 ^{13}C NMR spectra of compound **4e**

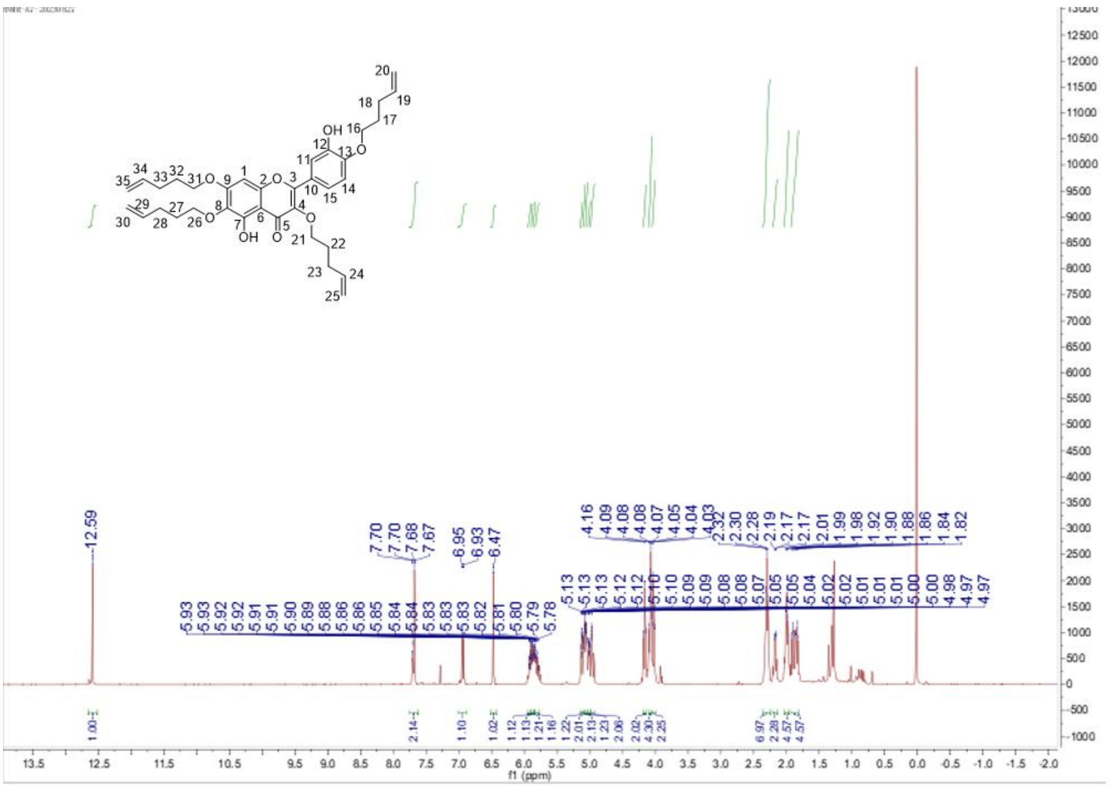

Figure S69 ^{1}H NMR spectra of compound **4f**

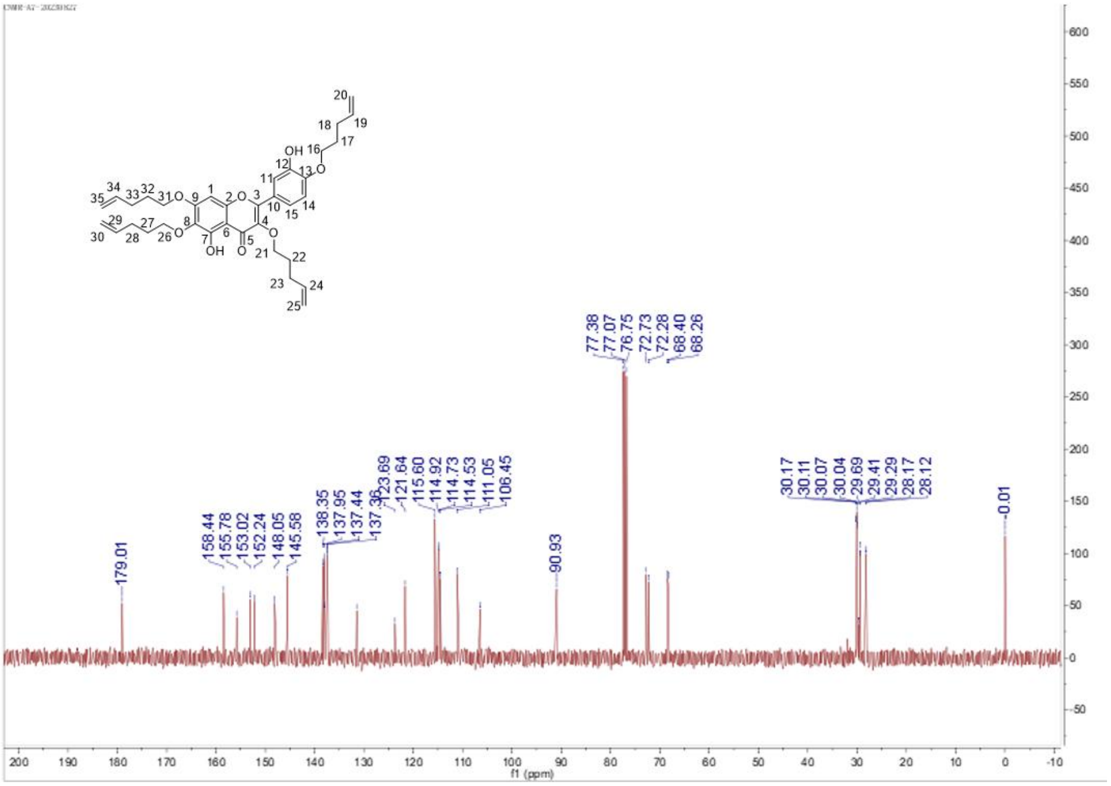

Figure S70 ^{13}C NMR spectra of compound **4f**

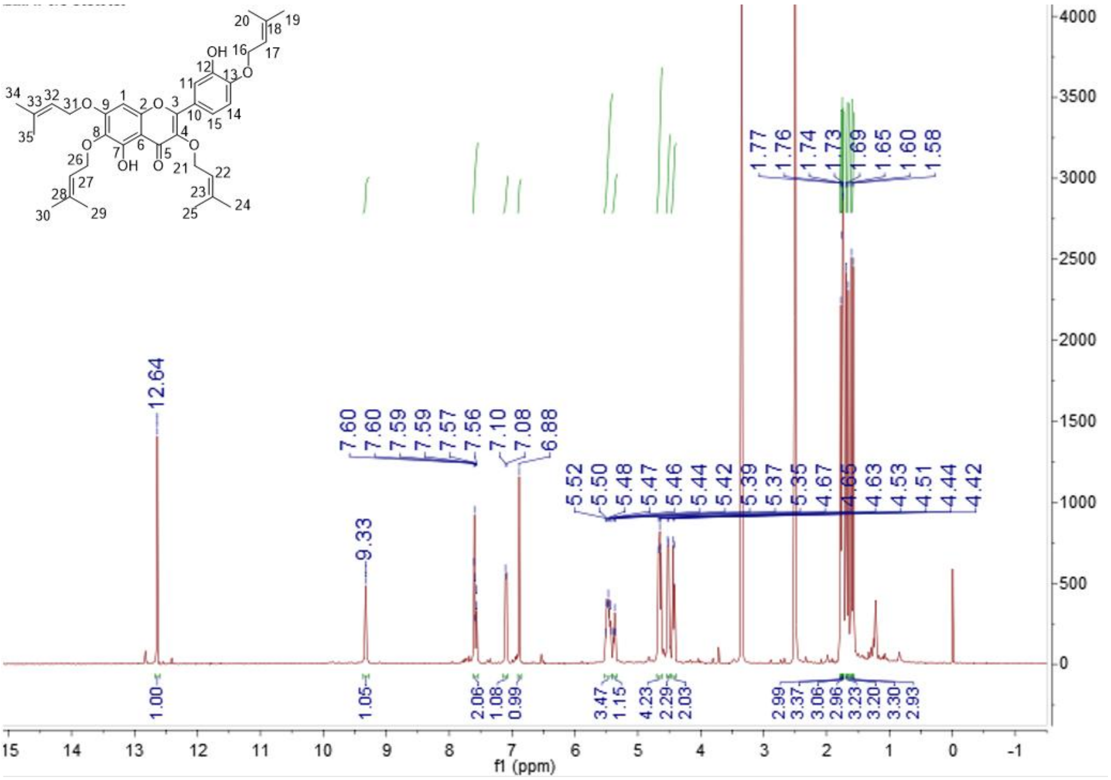

Figure S71 ^{1}H NMR spectra of compound **4g**

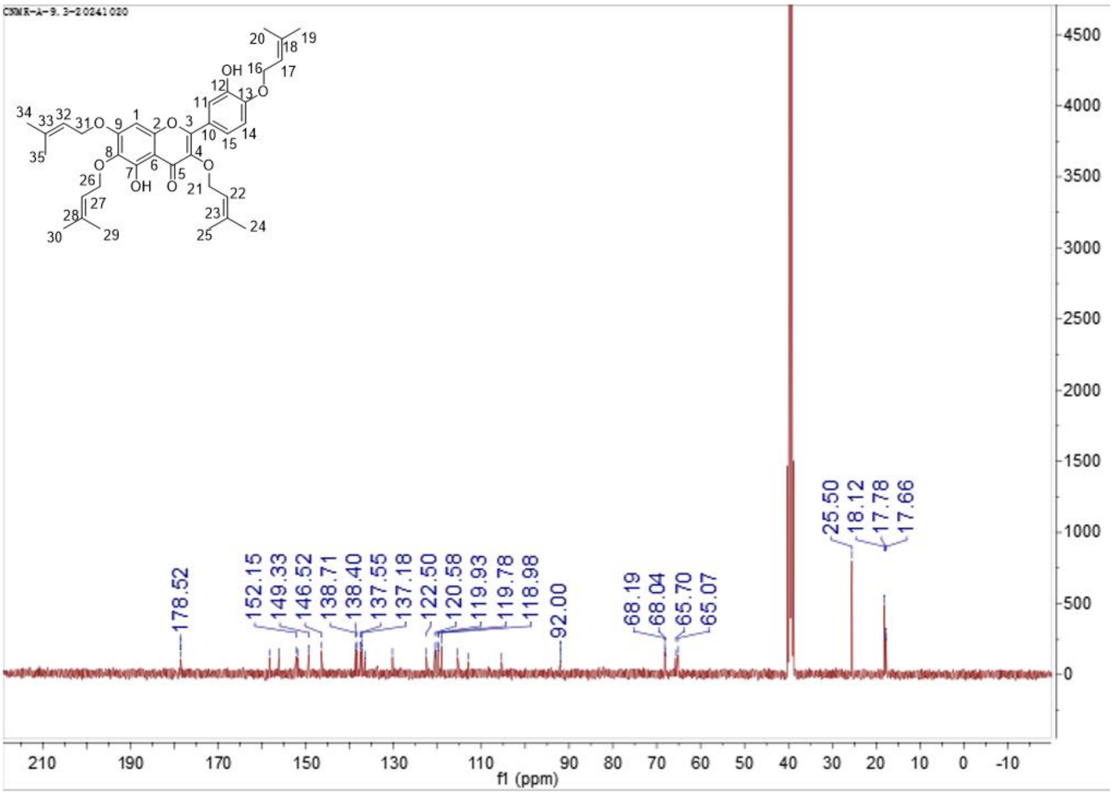

Figure S72 ^{13}C NMR spectra of compound **4g**

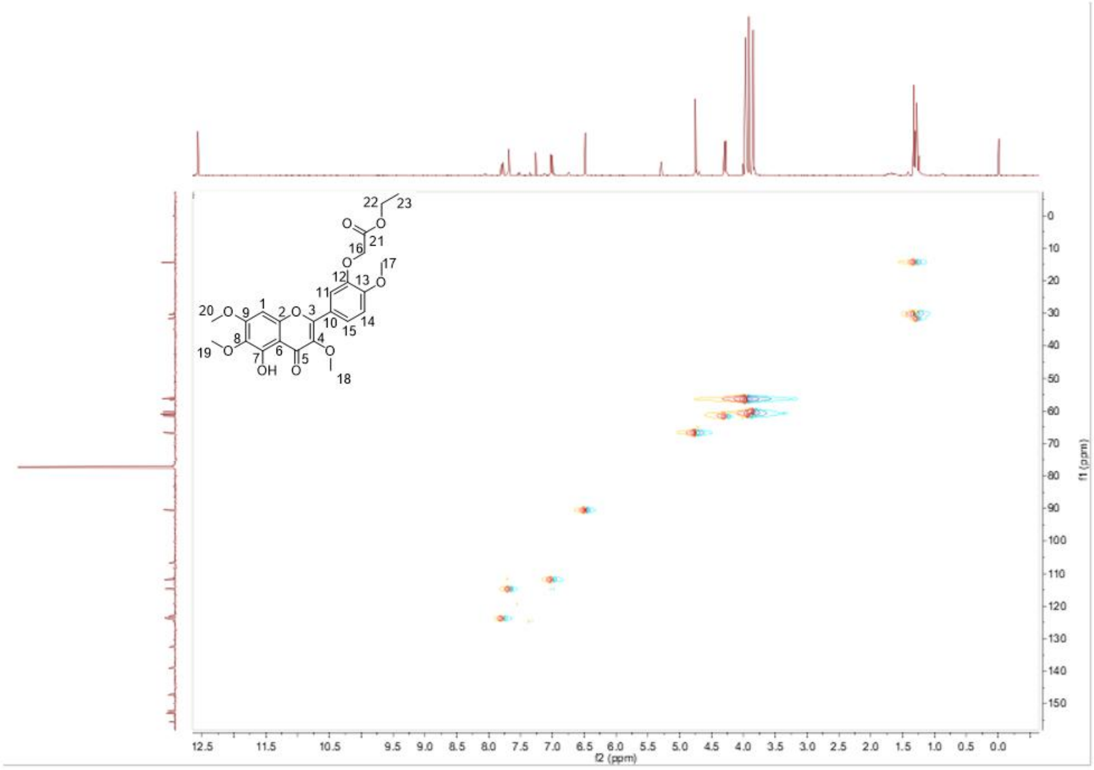

Figure S73 HSQC spectra of compound **2b**

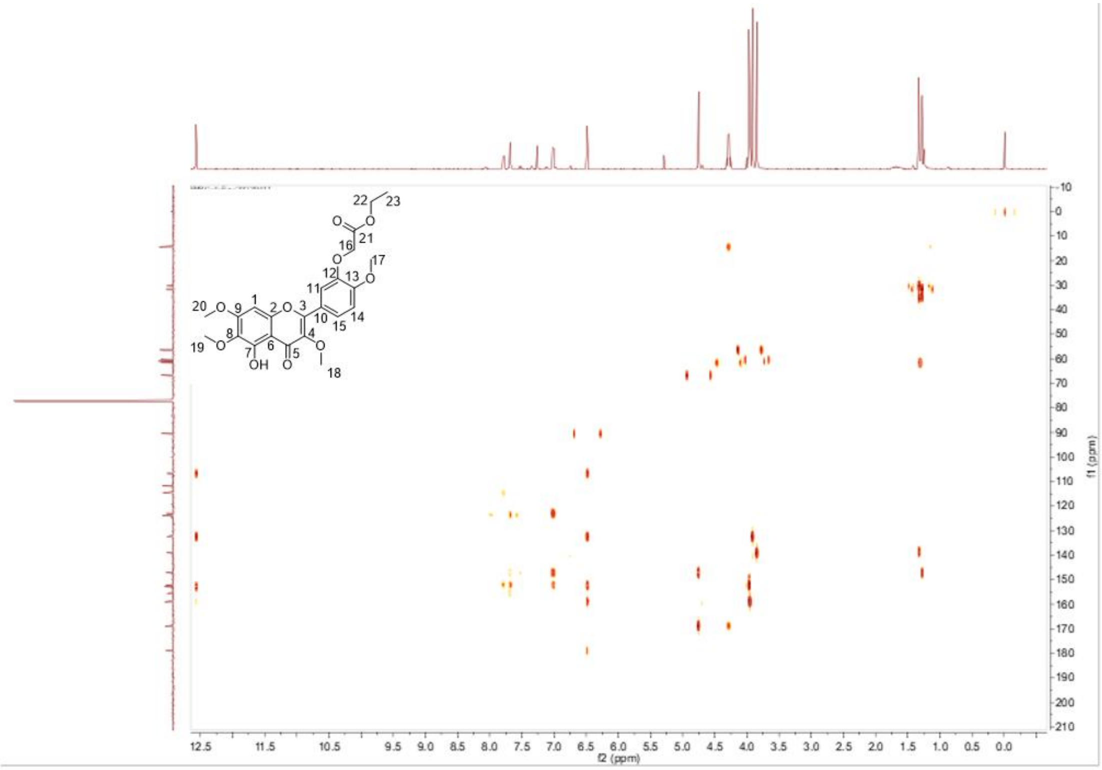

Figure S74 HMBC spectra of compound **2b**

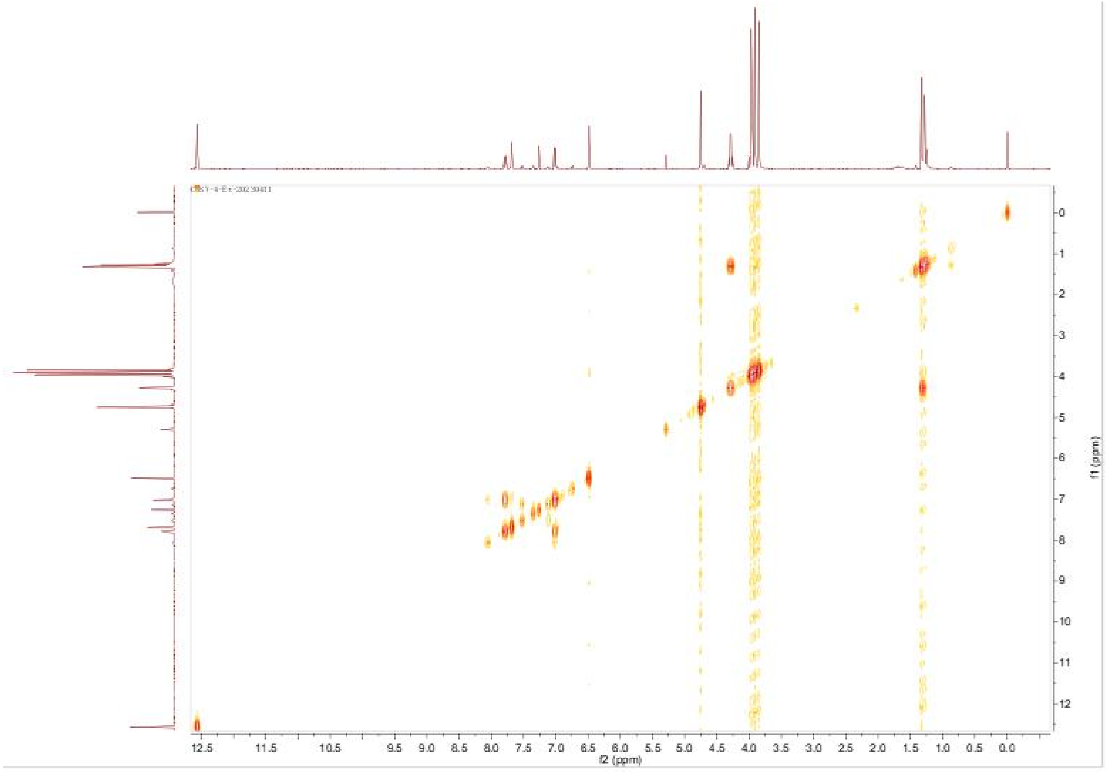

Figure S75 ^{1}H-^{1}H COSY spectra of compound **2b**

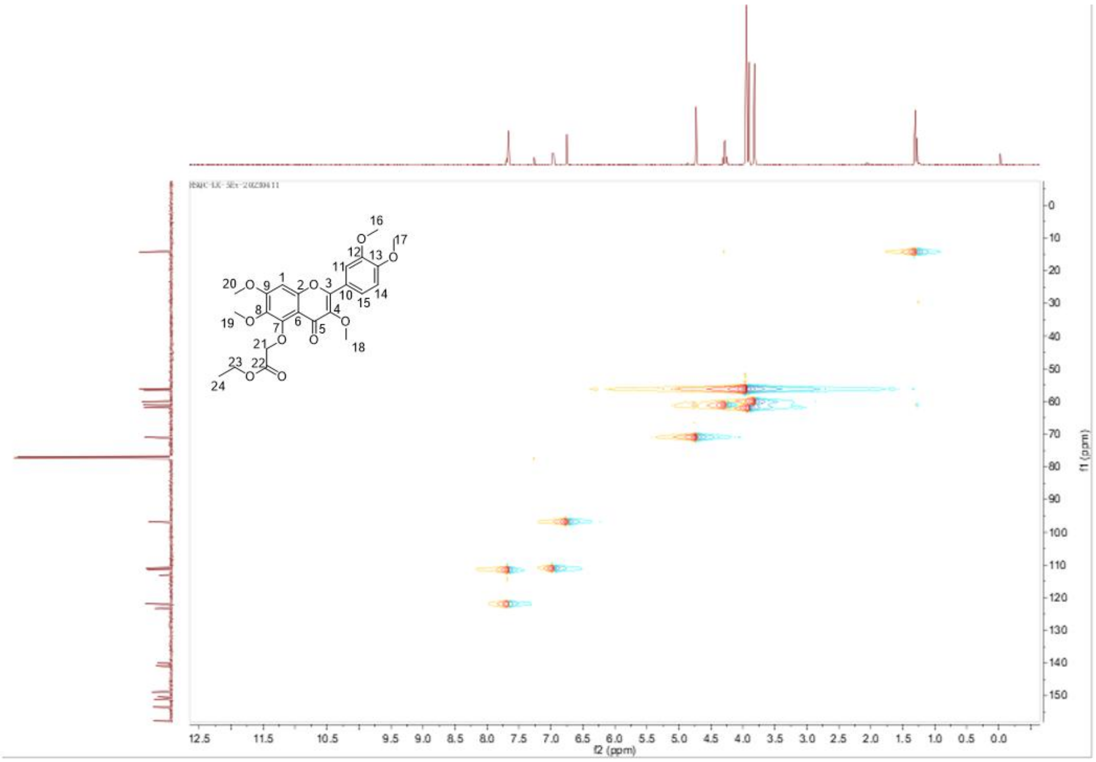

Figure S76 HSQC spectra of compound **3b**

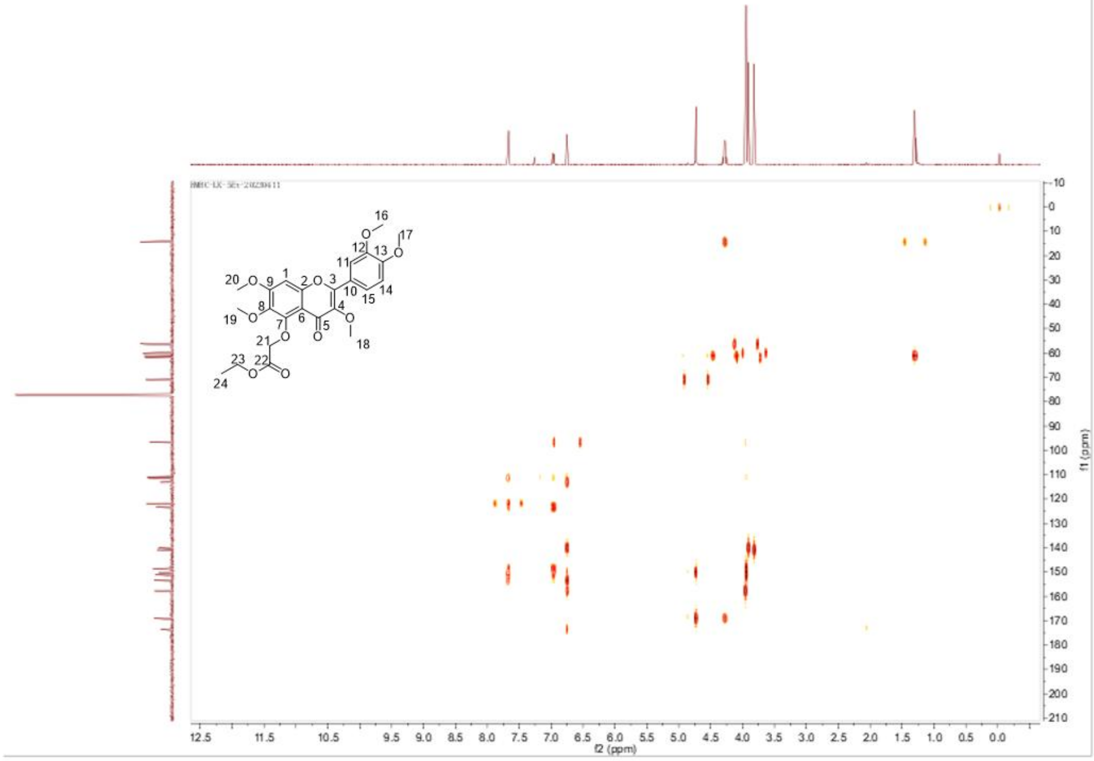

Figure S77 HMBC spectra of compound **3b**

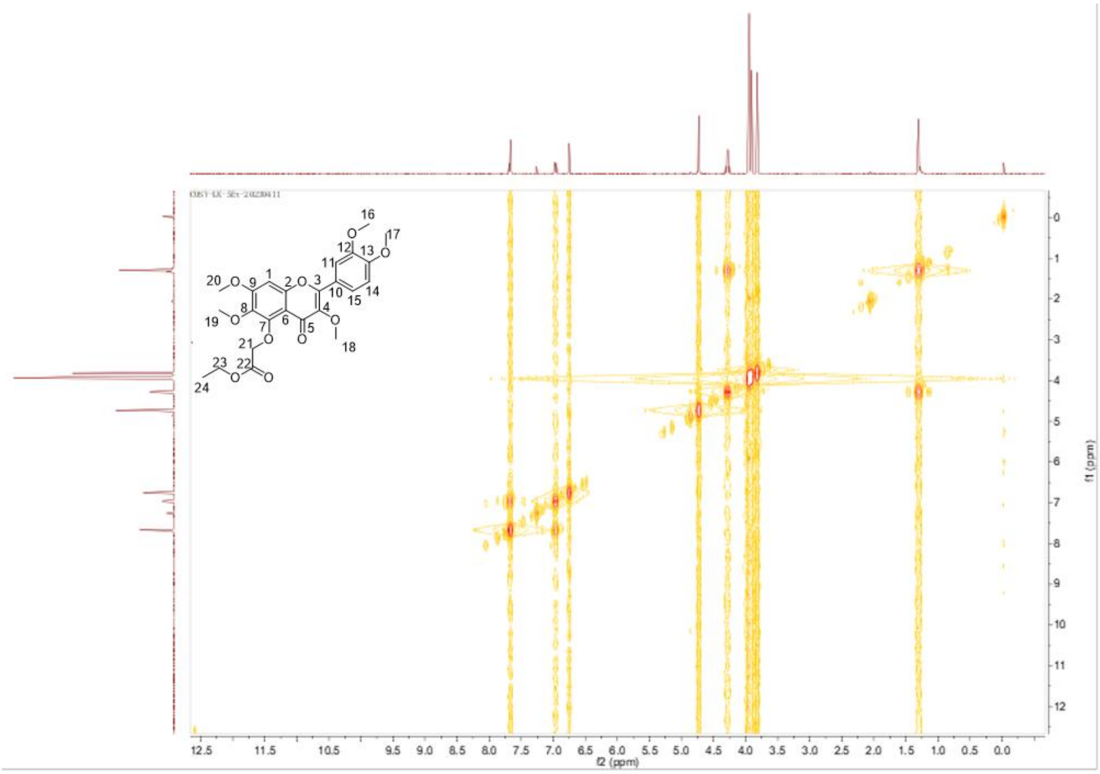

Figure S78 ^{1}H-^{1}H COSY spectra of compound **3b**
